# Inflammation-Related Genes Are Differentially Expressed in Lipopolysaccharide-Stimulated Peripheral Blood Mononuclear Cells after 3 Months of Resistance Training in Older Women

**DOI:** 10.3390/cells13171416

**Published:** 2024-08-25

**Authors:** Lene Salimans, Keliane Liberman, Wilfried Cools, Rose Njemini, Florence Debacq-Chainiaux, Louis Nuvagah Forti, Liza De Dobbeleer, Ron Kooijman, Ingo Beyer, Ivan Bautmans

**Affiliations:** 1 Frailty & Resilience in Ageing Research Unit (FRIA), Vitality Research Group, Vrije Universiteit Brussel (VUB), B-1090 Brussels, Belgium; 2 Gerontology Department, Vrije Universiteit Brussel (VUB), B-1090 Brussels, Belgium; 3 Biostatistics and Medical Informatics Department, Vrije Universiteit Brussel (VUB), B-1090 Brussels, Belgium; 4 URBC, NAmur Research Institute for LIfe Science (NARILIS), University of Namur, B-5000 Namur, Belgium; 5 Center for Neurosciences (C4N), Vrije Universiteit Brussel (VUB), B-1090 Brussels, Belgium; 6 Geriatrics Department, Universitair Ziekenhuis Brussel (UZB), B-1090 Brussels, Belgium; 7 Geriatric Physiotherapy Department, SOMT University of Physiotherapy, 3821 BN Amersfoort, The Netherlands

**Keywords:** older adults, exercise, LPS challenge, RNA sequence analysis, gene expression

## Abstract

Recently, we showed that three months of resistance exercise significantly alters 18 canonical pathways related to chronic inflammation in PBMCs of older adults. In this exploratory sub-study, the aim is to explore whether resistance exercise enhances the PBMCs stress response by mimicking an acute infection through in vitro LPS stimulation. Women (≥65 years) were randomly divided into intensive strength training (IST), strength endurance training (SET), or flexibility training (as control group, CON) groups. PBMCs were isolated and cultured with and without LPS for 24 h. Their RNA was analyzed via targeted RNA sequencing of 407 inflammation-related genes, with relevant fold-changes defined as ≤0.67 or ≥1.5 (3 months vs. baseline). A pathway analysis using ingenuity pathway analyses identified significant pathways among 407 genes with *p* < 0.05 and z-scores of ≤−2 or ≥2. Fourteen women were included in the analyses. A total of 151 genes with a significant fold-change were identified. In the CON group, a less-pronounced effect was observed. Strength training altered 23 pathways in the LPS-stimulated PBMCs, none of which overlapped between the IST and SET groups. A balanced exercise program that includes both IST and SET could beneficially adapt the immune responses in older adults by inducing alterations in the inflammatory stress response of PBMCs through different genes and pathways.

## 1. Introduction

Ageing is accompanied by a mildly elevated level of circulatory inflammatory cytokines, described as the chronic low-grade inflammatory profile (CLIP) [1]. One of the hallmarks of CLIP is the increased number of senescent T cells, which produce more inflammatory cytokines and reduce resistance to infections related to a deregulated inflammatory response [2,3].

Exercise is one of the most effective non-pharmacological interventions to counteract CLIP, and it has been shown to have anti-inflammatory effects [4]. Studies have shown that 6 to 12 weeks of resistance training can significantly reduce basal IL-6 levels, thus reflecting a decreased level of CLIP [5]. Exercise triggers an acute and brief release of myokines, which induce an anti-inflammatory effect by stimulating the production of anti-inflammatory cytokines by peripheral blood mononuclear cells (PBMCs). To date, IL-6 is the best-characterized myokine [6]. A single resistance training session has been shown to be sufficient to obtain an average increase of ~20% in circulating IL-6 in older adults (aged 60–80 years) [5].

One of the initial reactions to infection involves the binding of pathogen-associated molecular patterns (PAMPS) such as lipopolysaccharides (LPS) to pattern recognition receptors (PRRs) such as toll-like receptor 4 (TLR4). This interaction triggers the release of pro-inflammatory cytokines through the activation of the nuclear factor–kappa beta (NF-KB) transcription factor [7,8]. Exercise might also reduce the expression of TLR4, leading to lower LPS-induced cytokine release in the PBMCs of older adults [9]. Exercise is thus expected to diminish the basal inflammatory milieu while enhancing the stress response of PBMCs, reflecting improved immune functioning. It is hypothesized that senescent T cells may be particularly susceptible to exercise-induced apoptosis, creating a “vacant space” for the expansion of the naïve T lymphocyte population [10,11]. Repetitive physical exercise on a regular basis is therefore expected to reduce the presence of senescent immune cells and thus improve immunity at higher ages.

Various exercise interventions have been proposed to counteract CLIP in older adults, but no consensus yet exists regarding the most effective exercise modality. Several weeks of resistance training, either alone or combined with aerobic exercise, leads to a significant reduction in inflammation in older persons. Our recently published systematic review provides an overview of the beneficial effects of resistance exercise on immune cell function in young and older adults. Although changes in immune cell function occur after just one acute bout of resistance exercise, regular resistance exercise interventions are necessary to maintain these beneficial effects and promote better immunity and a lower CLIP in older adults. The aim of this exploratory sub-study was to investigate the alterations in the expression levels of inflammation-related genes in older women after a 3-month resistance training program. In this investigation, we utilized LPS stimulation of PBMCs isolated from whole blood cultures as a model to simulate an in vivo infection. By examining gene expression patterns following immune stimulation, this exploratory sub-study aims to uncover the initial effects of exercise on immune regulation. Moreover, it will provide valuable insights into the molecular mechanisms occurring in PBMCs following an infection.

## 2. Materials and Methods

### 2.1. Participants

**Figure 1:**
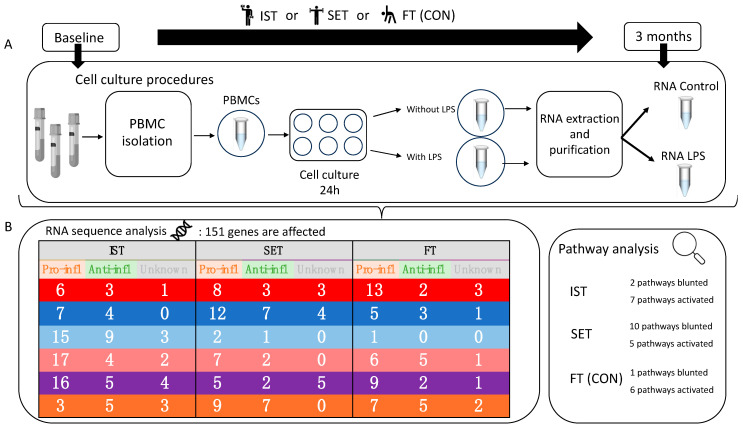
Overall overview of the exploratory sub-study design with results. (**A**) Older adults aged 65 years or more were randomized to one of the three intervention groups: intensive strength training (IST), strength endurance training (SET), or flexibility training (control group (CON)). Before and after three months of training, PBMCs were isolated and cultured (for 24 h) with and without LPS. RNA was collected from precultured, post-cultured, and LPS-stimulated PBMCs. (**B**) Summary of the results regarding the RNA sequence analysis and pathway analysis. An overview of the number of genes (pro-inflammatory, anti-inflammatory, and genes for which the exact immune-modulatory roles regarding exercise have not yet been described) showing a significant fold-change regarding the training-induced effects (FC ≤ 0.67 or FC ≥ 1.5). Dark red (UP 3 months > baseline): The number of genes showing a training-induced increase in upregulation following LPS stimulation. Pink (DOWN 3 months > baselineT0): The number of genes showing a training-induced increase in downregulation following LPS stimulation. Light blue (UP 3 months < baseline): The number of genes showing a training-induced decrease in upregulation following LPS stimulation. Dark blue (DOWN 3 months < baseline): The number of genes showing a training-induced decrease in downregulation following LPS stimulation. Purple (UP to DOWN): The number of genes showing a training-induced change in gene expression from upregulation to downregulation following LPS stimulation. Orange (DOWN to UP): The number of genes showing a training-induced change in gene expression from downregulation to upregulation following LPS stimulation. An overview of the affected pathways is also presented.

This is an explorative sub-study of the Senior Project Intensive Training (SPRINT) trial [12], which is an ongoing randomized controlled trial investigating the effects of exercise intervention on the immune system in independent community-dwelling older adults, aged 65 years and older. The SPRINT trial is a large intervention study evaluating the short-term (6 weeks), middle-term (12 weeks) [12], and long-term (6 months exercise and a follow-up period of 6 months detraining) effects of exercise on the immune systems of older adults. The participants engage in the exercise program for a minimum of 3 months and a maximum of 6 months. The protocol of the SPRINT study was approved by the local ethics committee in accordance with the Declaration of Helsinki (RIB approval codes: 2005/104 and 2011/257). The protocol of the SPRINT study was registered at clinicaltrials.gov (NCT04534049). Figure 1 shows an overview of the study design of this exploratory sub-study, which is focused on the effects after the 3-month training intervention.

In brief, each participant gave written informed consent prior to the medical examination. The exclusion criteria were as follows: using immunosuppressive or anti-inflammatory medication, unable to understand or execute the exercise instructions due to cognitive impairment (Mini Mental State Examination (MMSE) < 24/30 [13]), and performing regular physical exercise at higher intensities than habitual daily activity (e.g., fitness classes, cycling club, …) currently or within the past 6 months. The Charlson Comorbidity Index was used as a scoring system to quantify the severity of comorbidities at baseline. Comorbidities were not an exclusion criteria per se, but persons showing uncontrolled conditions and/or acute inflammation (C-reactive protein (CRP) > 10 mg/L) were excluded. The included participants were randomly assigned to either intensive strength training (IST), strength endurance training (SET), or the control group (CON) in which flexibility exercises were performed. The groups were randomized using a computer, analyzed by a researcher who was blinded to the study outcomes, and stratified according to sex (male/female), age (65–74 years, ≥75 years), and health category (based on a modified SENIEUR’s protocol, described by our research group [12]).

This exploratory sub-study is based on the same sub-sample of older female participants described in the paper by Liberman et al. [12]. Only women were included, since the target sample size for female participants had been reached [12]. Women who completed the 3-month exercise intervention without falling ill during the training period and had not altered their medication regimen for at least six weeks prior to blood sampling were eligible for further analysis. Additionally, RNA samples with an RNA quality number (RQN) greater than 7 and a yield exceeding 8 ng/µL were selected for further analysis.

### 2.2. Intervention

The exercise interventions were previously described by our research group [12]. In brief, each training session started with a warm-up consisting of 10 exercises, including mobility and activating exercises for both upper and lower limbs, to prepare the muscles and joints for the upcoming exercises. No external resistance was used.

The two resistance exercise interventions (IST and SET) consisted of chest press, leg press, hip abduction, hip adduction, low row, and vertical traction exercises, all performed seated. Both protocols were designed to be approximately equal in volume and based on the participants’ repetitions maximum (RM). One repetition maximum (1 RM) is the maximum load that can be moved once over the whole range of movement. The IST group performed 3 sets of 10 consecutive repetitions at 80% of 1 RM, in contrast to the SET group, in which 2 sets of 30 consecutive repetitions at 40% of 1 RM were performed. Between two sets, a minimum 1 min rest period was inserted.

The CON group performed flexibility training consisting of 3 sets of 13 sustained (30 s) passive, static stretching exercises of the large muscle groups. Flexibility exercises were chosen as control intervention because they mainly induce a passive load on the muscles and tendons without muscle contraction or cardiovascular impacts.

The participants were asked to perform the exercises 2 to 3 times per week. All the exercise programs (IST, SET, and CON) included an initial accommodation period of 2 weeks in which the target exercise intensities were progressively reached.

The individuals’ 1 RM was determined every six training sessions. A minimum of 24 h rest (no exercise) was scheduled before and after the 1 RM, and exercise loads were adapted accordingly. The 1 RM determination started with a warm-up consisting of 20 repetitions at 30% of 1 RM (estimated or previously assessed). After the warm-up, the participants started to perform one repetition at 70% of 1 RM (estimated or previously assessed). This step was repeated to reach the maximum load that could be moved once correctly in full range of motion. The aim was to reach the 1 RM in a maximum of 4 to 5 steps.

All the training sessions and 1 RM determinations took place at the exercise facilities of the Brussels Health Campus of the Vrije Universiteit Brussel on Technogym™ (Technogym, Gambettola, Italy) and Matrix (Matrix, WI, USA) single station cable-type devices (for detailed specifications see manufacturers’ websites: https://www.technogym.com/be/fr/ (accessed on 19 August 2024) and https://www.matrixfitness.com/nl/dut (accessed on 19 August 2024)).

At baseline and after 3 months of training, the muscle strength index (MSI) was determined by averaging the 1 RM values obtained from the 6 strength training exercises. The participants were instructed to train three times a week for three months, with a target total of 36 sessions over 3 months. The number of training sessions that were effectively performed was recorded and expressed as a percentage of the target number. The Yale Physical Activity Survey (YPAS) was used to assess the physical activity of the older adults [14].

### 2.3. PBMC Isolation, Culture, and RNA Extraction

The PBMC isolation, culture, and RNA extraction followed the same procedure as those previously published by Liberman et al. [12]. Whole blood samples were collected at baseline and following the 3-month exercise intervention, with each sample being drawn at least 24 h after the final training session. PBMCs were subsequently isolated using density gradient centrifugation using lymphoprep (density gradient: 1.077 ± 0.001 g/mL, Axis-Shield, Oslo, Norway). The isolated PBMCs were cultured in RPMI 1640 medium (Sigma, Saint Quentin Fallavier, France), which was supplemented with 10% fetal bovine serum (FBS) (Thermo Fisher Scientific, Waltham, MA, USA) and an antibiotic–antimycotic solution (10,000 units/mL of penicillin, 10,000 µg/mL of streptomycin (Thermo Fisher Scientific, Waltham, MA, USA), and 25 µg/mL of Gibco Amphotericin B (Thermo Fisher Scientific, Waltham, MA, USA). The cultures were prepared both with and without LPS, with a final concentration of 0.025 mg/mL (S. enterica, Sigma Aldrich, St Louis, MO, USA). The RNA was extracted from the PBMCs prior to culturing and after a 24 h incubation period under conditions of 37 °C and 5% CO_2_, both in the presence and absence of LPS.

The RNA was purified using the GeneJet RNA purification kit (Thermo Fisher Scientific, Waltham, MA, USA) following the manufacturer’s protocol. The purified RNA was immediately stored at −80 °C until simultaneously assayed.

### 2.4. RNA Quality Assessment and RNA Sequencing

The RNA quality assessment and RNA sequencing were previous described in Liberman et al. [12]. The quality of the total RNA samples was assessed using an AATI Fragment Analyzer (Agilent Technologies Inc., Santa Clara, CA, USA), using the DNF-472 high-sensitivity RNA analysis kit. The women who completed the 3-month exercise intervention without falling ill during the training period and had not altered their mediation regimen for at least six weeks prior to blood sampling were eligible for further analysis. RNA samples with an RNA quality number (RQN) greater than 7 and a yield exceeding 8 ng/µL were selected for further analysis.

RNA libraries were created from 150 ng of total RNA according to the manufacturer’s instructions (KAPA RNA HyperPrep Kit with RiboErase kit (Roche Diagnostics, Vilvoorde, Belgium)). In summary, after ribodepletion and DNase digestion, the RNA was fragmented, incubating the samples for 6 min at 94 °C (final average size: 200–300 bp). After first strand synthesis, second strand synthesis, and adapter ligation, the libraries were amplified using 12 PCR cycles. Subsequently, 12 libraries were pooled by equal mass (83.3 ng each) for a total of 1 µg of cDNA per library and captured according to the Roche SeqCap RNA Enrichment System User’s Guide v1.1 (Roche Diagnostics, Vilvoorde, Belgium), with 2 modifications: (1) the usage of xGen Universal Blockers TS Mix (Integrated DNA Technologies, Coralville, IA, USA) in the capturing reaction and (2) limiting the PCR to 12 PCR cycles.

The final libraries were using an AATI Fragment Analyzer (Agilent Technologies Inc., Santa Clara, CA, USA), using the DNF-474 high-sensitivity NGS fragment analysis kit and quantified using a Qubit 2.0 using a Qubit dsDNA HS assay hit (Life Technologies, Carlsbad, CA, USA).

Each sample was sequenced to generate 25 million 2 × 100 bp reads were generated using an Illumina NovaSeq 6000 system (Illumina Inc., San Diego, CA, USA) utilizing the NovaSeq 6000 S2 reagent kit (200 cycles). The libraries were denatured to a concentration of 1.9 nM following the manufacturer’s instructions.

For the data analysis, STAR (version 1.5) was used for alignment, and htseq-count (version 0.11.0) was employed to obtain the counts.

### 2.5. Statistical Analyses and Pathway Analyses

The baseline characteristics of the three intervention groups were analyzed using a one-way ANOVA with LSD post hoc analyses for normally distributed data. A repeated measures ANOVA was used to determine the changes in MSI, with time (baseline—3 months) as the within-subject factor and the intervention groups as the between-subjects factors. For non-normally distributed data, the Kruskal–Wallis test was used to determine the baseline differences between the groups. To determine the changes after the intervention, the Wilcoxon-signed ranked test was used. All of above-mentioned analyses were performed using IBM SPSS^®^ version 25.0.

The sample size power calculation using G-Power^®^ version 3.1 [15] revealed that 4 participants per group allowed an exercise-induced gene expression fold change of at least 1.5 (power = 0.82, alpha = 0.017).

Differentially expressed genes were identified using DESeq2 version 1.22.2 [16] in Rstudio version 1.1.463 (Boston, MA). After normalization of the count files, the log_2_ fold change (FC) was calculated for each group separately. The log_2_FC was then back-transformed to obtain an absolute FC for all the genes. For the LPS-stimulated PBMCs, to eliminate the effects caused by the culture itself, first, the differences between the post-culture PBMCs without LPS stimulation (post-culture condition) and the LPS-stimulated PBMCs (LPS condition) at baseline and after 3 months were computed. Next, to identify the proportional change over time, the difference in LPS vs. post-culture conditions between the two time points was calculated (FC_(LPS/post-culture)3months_/FC_(LPS/post-culture)baseline_). All the genes with an absolute FC of ≤0.67 or ≥1.5 were considered clinically relevant. All the genes occurring with a clinically relevant FC in at least one of the intervention groups were pooled and compared with the corresponding FC values of the other intervention groups.

These genes were classified into pro-inflammatory genes, anti-inflammatory genes, or genes whose functions in inflammation were not yet fully determined, according to the known function in gene databases from the National Center for Biotechnology Information (NCBI) (Available from: https://www.ncbi.nlm.nih.gov/gene/ (accessed on 19 August 2024)) and previously published literature on these genes. Moreover, the genes were classified according to their changes in expression patterns, and six groups were identified.

1. **Exercise-induced increase in upregulation following LPS stimulation**, which means that the LPS stimulation upregulates the gene at baseline and after three months, and the expression is higher after the three-month exercise intervention.
  (Effects after three months exercise intervention > Effects baseline UP)
2. **Exercise-induced increase in downregulation following LPS stimulation**, which means that the LPS stimulation downregulates the gene at baseline and after three months, and the expression is lower after the three-month exercise intervention.
  (Effects after three months exercise intervention > effects baseline DOWN)
3. **Exercise-induced decrease in upregulation following LPS stimulation**, which means that the LPS stimulation upregulates the gene at baseline and after three months, and the expression is lower after the three-month exercise intervention.
  (Effects after three months exercise intervention < effects baseline UP)
4. **Exercise-induced decrease in downregulation following LPS stimulation**, which means that the LPS stimulation downregulates the gene at baseline and after three months, and the expression is lower after the three-month exercise intervention.
  (Effects after three months exercise intervention < effects baseline DOWN)
5. **Exercise-induced change from upregulation to downregulation following LPS stimulation**, which means that the LPS stimulation upregulates the gene at baseline and downregulates it after three months.
  (UP to DOWN)
6. **Exercise-induced change from downregulation to upregulation following LPS stimulation**, which means that the LPS stimulation downregulates the gene at baseline and upregulates it after three months.
  (DOWN to UP)

An ingenuity pathway analysis (IPA) was performed on all the genes obtained from this RNAseq experiment. Only immune cells and immune cell lines were included as tissue types, excluding cancer-related and xenobiotic pathways. Pathways with a Benjamini–Hochberg-adjusted *p*-value of <0.05 and a z-score of ≤−2 or ≥2 were considered significant. Z-scores were determined based on the IPA, considering gene activation or inactivation based on fold-changes and their causal relationships. Irrelevant pathways, including those related to sperm mobility, endometrial cancer signaling, and acute myeloid leukemia signaling, were excluded from the analysis.

## 3. Results

### 3.1. Baseline

The characteristics of the participants were previously described by Liberman et al. (2022) [12]. In brief, out of the fourteen women, four were randomized into the IST group, five were in the SET group, and five were in the CON group. There were no differences between the three intervention groups at baseline. However, it is noteworthy that the percentage of monocytes was slightly higher in the IST group compared to SET, with this difference being statistically significant (Table 1). There were no significant changes in the total counts of white blood cells (WBC), neutrophils, monocytes, eosinophils, basophils, or lymphocytes, nor in the proportions of CD8^+/−^ cells, senescent-prone (CD8^+/−^CD28^+/−^CD57^+^ and CD8^+/−^CD57^+^), memory (CD8^+/−^CD28^−^CD57^+^), or naïve (CD8^+/−^CD28^+^CD57^+^) cells following the three-month exercise intervention [12].

As reported previously [12], muscle strength increased significantly after 3 months IST and SET (Table 2).

### 3.2. Exercise-Induced Gene Expression Changes in Post-Cultured LPS-Stimulated PBMCs

**Figure 2:**
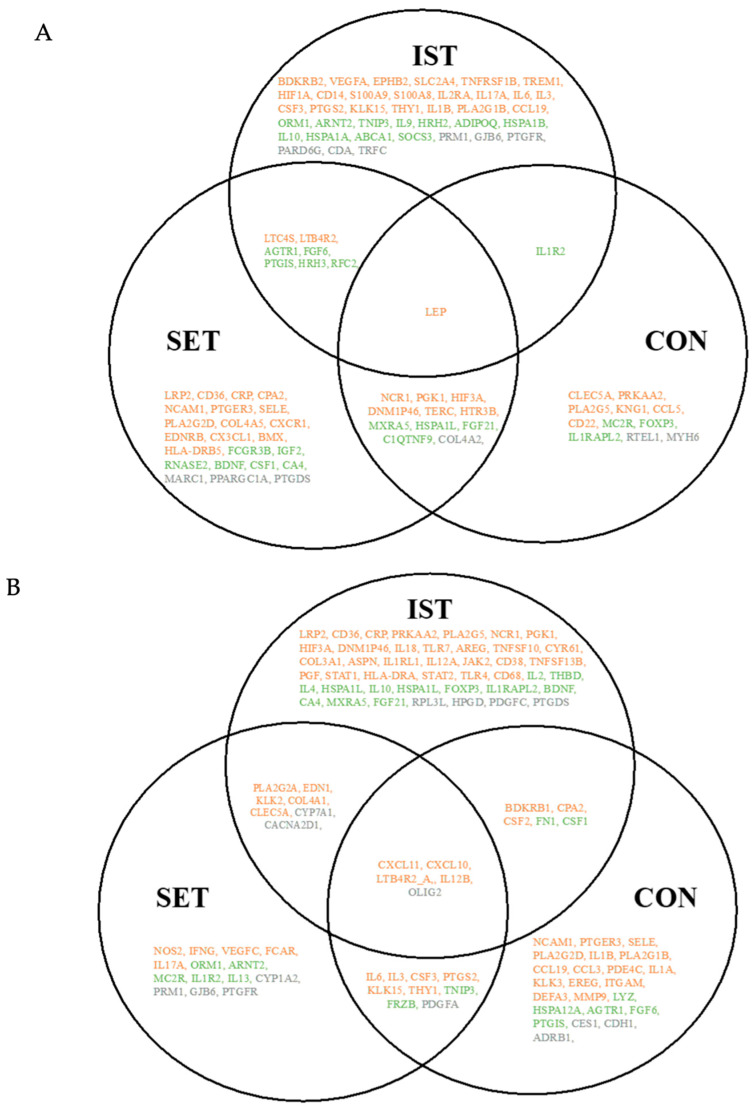
Venn diagram illustrating the overlap between the exercise intervention groups. In total, 151 genes were significantly expressed after the 3-month intervention, including 90 pro-inflammatory genes (orange), 38 anti-inflammatory genes (green), and 23 genes whose roles in inflammation have not yet been fully determined (grey). The full gene abbreviations can be found in Supplementary Table S1. (**A**) Downregulated genes with a fold-change between 0.67 and 1.5 (cut-off +/−2). (**B**) Upregulated genes with a fold-change between 0.67 and 1.5 (cut-off +/−2). Note: Some genes exhibited different expression patterns across the various intervention groups and are therefore duplicated in figure (**A**,**B**), IST: intensive strength training, SET: strength endurance training, CON: flexibility training (control group).

A total of 151 genes exhibited a significant fold-change in gene expression level in the LPS-stimulated PBMCs as a result of the exercises (Figure 2). Among these, 90 genes were identified as pro-inflammatory genes, 38 were identified as anti-inflammatory genes, and 23 genes played roles in inflammation that are not yet fully elucidated (Figure 2 and Supplementary Table S1). Different expression patterns were noticed across the three intervention groups (IST, SET, and CON). The majority of the pro-inflammatory genes showed significant fold-changes after the exercise intervention following LPS stimulation (IST: 64 genes, SET: 43 genes, CON: 41 genes) (Supplementary Table S1).

On the other hand, the majority of the anti-inflammatory genes showed a clinically relevant absolute FC in the IST group (n = 30 genes), whereas the SET and CON groups showed fewer genes with significant fold-changes (n = 22 genes and n = 17 genes, respectively) (Supplementary Table S1). The number of genes with unknown roles in inflammation that showed significant fold-changes were comparable between the IST and SET groups (13 genes and 12 genes, respectively), whereas the CON group showed fewer genes with significant fold-changes (8 genes) (Supplementary Table S1).

Supplementary Table S2 shows a more detailed overview of the exercise-induced changes in gene expression post-LPS-stimulated PBMCs subdivided into the six categories presented in the methods section.

#### 3.2.1. Exercise-Induced Increase in Upregulation or Downregulation (Enhanced Effects)

**Figure 3:**
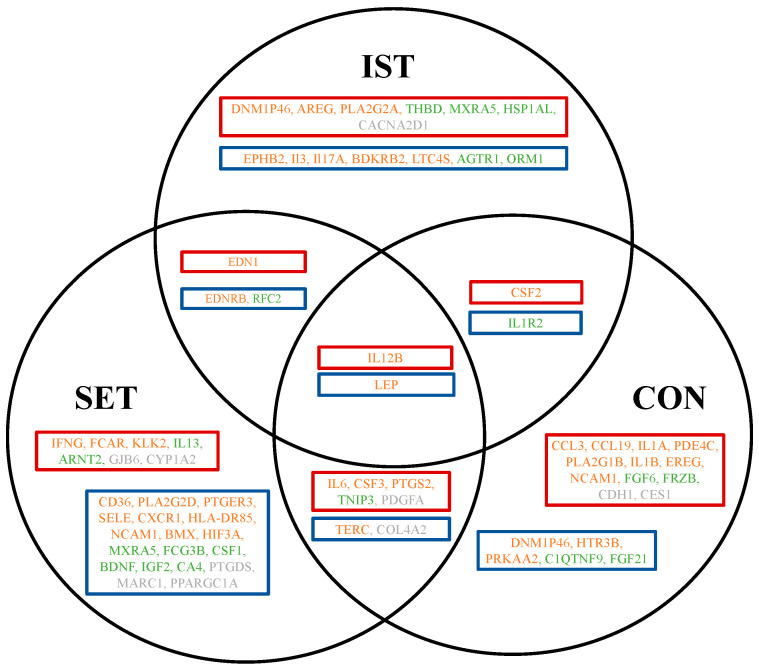
Venn diagram presenting the overlap of the exercise-affected genes following LPS stimulation. Red boxes represent genes showing an exercise-induced increase in upregulation following LPS stimulation; blue boxes represent exercise-induced decrease in downregulation following LPS stimulation. Only significantly affected genes are shown. Downregulated genes with a fold-change between 0.67 and 1.5 (cut-off +/−2) and upregulated genes with a fold-change between 0.67 and 1.5 (cut-off +/−2). In total, 86 genes were significantly expressed after the 3-month intervention, with 43 genes showing an exercise-induced enhanced effect and 43 genes presenting an exercise-induced attenuated effect following LPS stimulation. The genes are categorized as follows: 52 pro-inflammatory genes (orange), 22 anti-inflammatory genes (green), and 12 genes with undetermined roles in inflammation (grey). The full gene abbreviations are presented in Supplementary Table S1. IST: intensive strength training, SET: strength endurance training, CON: flexibility training (control group).

The genes that exhibited significant downregulation or upregulation after the exercise intervention following LPS stimulation showed a more pronounced decrease or increase in expression compared to the baseline following LPS stimulation. These genes, demonstrating a significantly enhanced exercise-induced effect in upregulation or downregulation following LPS stimulation, are depicted in Figure 3 and Supplementary Table S1.

The majority of genes showing an enhanced exercise-induced effect following LPS stimulation were in the SET group (n = 37), including 20 pro-inflammatory genes. In the CON group, 28 genes showed an exercise-induced effect, with 19 genes being more upregulated and nine genes being more downregulated post-intervention following LPS stimulation. Only 21 genes showed an exercise-induced enhanced effect following LPS stimulation in the IST group, comprising 13 pro-inflammatory genes, 7 anti-inflammatory genes, and 1 gene with an undetermined role in inflammation.

#### 3.2.2. Exercise-Induced Decrease in Upregulation and Downregulation (Attenuated Effects)

**Figure 4:**
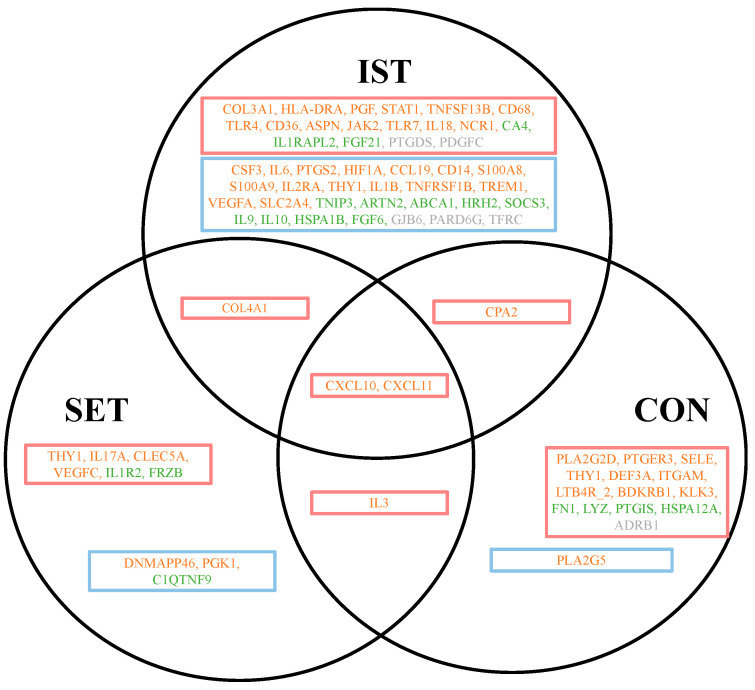
Venn diagram presenting the overlap of exercise-affected genes following LPS stimulation. Pink boxes represent genes showing an exercise-induced decrease in downregulation following LPS stimulation; light-blue boxes represent exercise-induced increases in upregulation following LPS stimulation. Only significantly affected genes are shown, including downregulated genes with a-fold change of between 0.67 and 1.5 (cut-off +/−2) and upregulated genes with a fold change of between 0.67 and 1.5 (cut-off +/−2). In total, 80 genes were significantly expressed after the 3-month intervention, with 31 genes showing an exercise-induced attenuated effect in upregulation following LPS stimulation and 49 genes representing an exercise-induced attenuated effect in downregulation following LPS stimulation. Among these, 56 were pro-inflammatory genes (orange), 18 were anti-inflammatory genes (green), and 6 genes had undetermined roles in inflammation (grey). The full gene abbreviations are presented in Supplementary Table S1. IST: intensive strength training, SET: strength endurance training, CON: flexibility training (control group).

The genes exhibiting significant downregulation or upregulation after the exercise intervention following LPS stimulation showed a less-pronounced decrease or increase in expression compared to the baseline following LPS stimulation. These genes, demonstrating significantly attenuated exercise-induced effects in upregulation or downregulation following LPS stimulation, are depicted in Figure 4 and Supplementary Table S1.

The exercise-induced upregulation of 31 genes and downregulation of 44 genes was attenuated following LPS stimulation. Among these, the majority were pro-inflammatory genes (n = 48), followed by anti-inflammatory genes (n = 21) and genes with undetermined roles in inflammation (n = 6). In the IST group, 50 genes exhibited an exercise-induced attenuated effect, with 32 pro-inflammatory genes affected (15 genes showed less upregulation and 17 genes showed less downregulation after the exercise intervention). In contrast, the SET and CON groups exhibited fewer genes affected by the exercise-induced attenuation effect, with 12 and 13 genes, respectively, compared to the IST group.

Forty-eight genes showed an exercise-induced attenuation effect following LPS stimulation after IST, comprising 31 pro-inflammatory genes, 12 anti-inflammatory genes, and 5 genes with undetermined roles in inflammation. In the SET group, 13 genes exhibited an exercise-induced attenuation effect following LPS stimulation, of which were three were anti-inflammatory genes (IL1R2, FRZB, and C1QTNF9). Three genes (DNMAPP46, PGK1, and C1QTNF9) were less upregulated after three months of training. In the CON group, 19 genes showed an exercise-induced attenuation effect following LPS stimulation. Among these, one pro-inflammatory gene, PLA2G5, was less upregulated after three months of training.

#### 3.2.3. Exercise-Induced Changes in Down- or Upregulation (Changed Effects)

**Figure 5:**
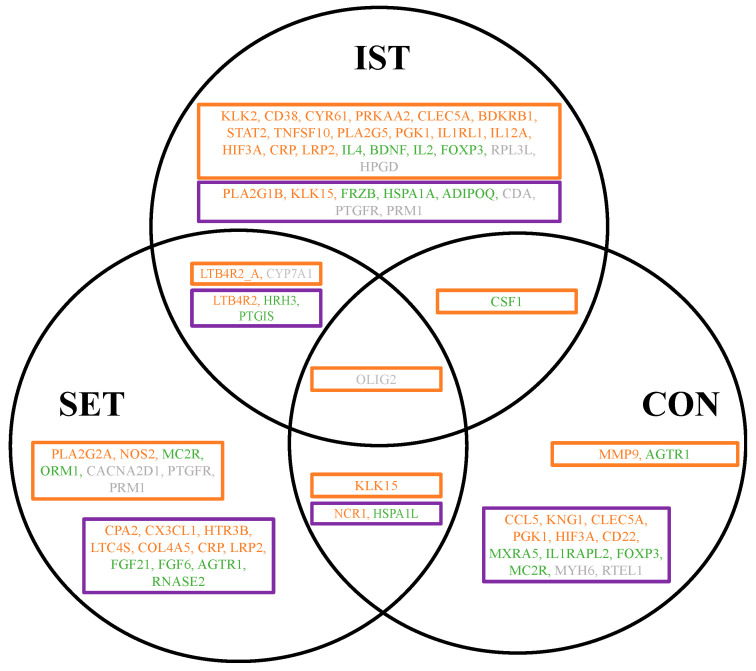
Venn diagram presenting the overlap of the exercise-affected genes following LPS stimulation. Orange boxes represent genes showing an exercise-induced change from downregulation to upregulation following LPS stimulation, purple boxes represent exercise-induced change from upregulation to downregulation following LPS stimulation. Only significantly affected genes are shown, including downregulated genes with a fold-change of between 0.67 and 1.5 (cut-off +/−2) and upregulated genes with a fold change of between 0.67 and 1.5 (cut-off +/−2). In total, 81 genes were significantly changed after the 3-month intervention, with 41 genes showing an exercise-induced change from downregulation to upregulation following LPS stimulation and 40 genes presenting an exercise-induced changed from upregulation to downregulation following LPS stimulation. Among these genes, 40 were pro-inflammatory genes (orange), 26 were anti-inflammatory genes (green), and 15 had undetermined roles in inflammation (grey). The full gene abbreviations are presented in Supplementary Table S1. IST: intensive strength training, SET: strength endurance training, CON: flexibility training (control group).

Genes exhibiting significantly altered gene expression after the exercise intervention following LPS stimulation showed a transition from up- to downregulation or down- to upregulation compared to the baseline following LPS stimulation. These genes, demonstrating a significantly changed exercise-induced effect in upregulation or downregulation following LPS stimulation, are depicted in Figure 5 and Supplementary Table S1.

A total of 90 genes exhibited significantly altered exercise-induced effects, with 49 genes transitioning from downregulation to upregulation and 41 genes transitioning from upregulation to downregulation. Among these genes, 49 were pro-inflammatory genes, 26 were anti-inflammatory genes, and 15 had undetermined roles in inflammation. In the IST group (n = 36), the majority of the genes were significantly affected by exercise-induced effects compared to the SET (n = 28) and FT (n = 26) groups. Most pro-inflammatory genes (n = 16) changed from downregulation to upregulation following LPS stimulation, with a significant fold-change in the IST group. Thirty-six genes exhibited an exercise-induced changed following LPS stimulation after IST, comprising 19 pro-inflammatory genes, 10 anti-inflammatory genes, and 7 genes with undetermined roles in inflammation. In the SET group, 27 genes showed exercise-induced changes following LPS stimulation. In the CON group, 19 genes showed exercise-induced changes following LPS stimulation.

#### 3.2.4. Exercise-Induced Upregulated Effects vs. Exercise-Induced Downregulated Effects

Figure 5 provides an overview of the genes exhibiting significant fold-changes after exercise with LPS stimulation, sorted based on their exercise-induced effects in each intervention group. This section primarily focuses on the genes showing different expression patterns among the three intervention groups. Overall, in the IST group, there was a higher upregulation of pro-inflammatory genes, anti-inflammatory genes, and genes whose role in inflammation have not yet been determined, compared to both the SET and CON groups. Additionally, higher fold-changes were observed for the genes showing exercise-induced upregulation following LPS stimulation compared to the genes showing an exercise-induced downregulation following LPS stimulation.

### 3.3. Pathway Analyses

In total, 29 canonical pathways were significantly altered after 3 months of RE in LPS-stimulated PBMCs in the trained older adults (Table 3). The affected pathways with the involved genes are presented in Supplementary Figure S1.

Strength training, both IST and SET, altered 23 pathways in the LPS-stimulated PBMCs. Seven pathways were upregulated by IST, in contrast to two pathways that were downregulated. More of the pathways were downregulated in the SET compared to upregulated pathways: ten and five, respectively. Moreover, no overlap was observed in the pathways between IST and SET except for one pathway, the cytotoxic T lymphocyte-mediated apoptosis of target cells, which was oppositely affected in IST and SET: downregulated and upregulated, respectively. Eight pathways were altered in the CON, of which one pathway, the apelin cardiac fibroblast signaling pathway, was downregulated.

## 4. Discussion

In this article, we present the results of an exploratory sub-study of a three-month exercise intervention study comparing high-load (IST) and moderate-load (SET) resistance exercise with an active control group performing flexibility exercises (CON) (Figure 1). To the best of our knowledge, this is the first study employing this complex approach involving LPS-induced in vitro manipulation of PBMCs to investigate inflammation-related gene expression changes in PBMCs after two different resistance exercise programs (moderate versus high intensity) over a three-month period in older adults. While Abbasi et al. [8] employed a comparable study design, they focused on investigating the acute effects of an aerobic exercise intervention on LPS-stimulated whole blood cultures.

The participant characteristics showed that the resistance exercise elicited significant physiological adaptations. The overall muscle strength improved significantly during the three-month intervention in both the IST and SET groups: +35% and +33%, respectively [12]. In the gene expression analysis, 151 genes were identified, with a significant fold-change after the exercise intervention in PBMCs with LPS stimulation. Among these, 148 genes were affected by one or both of the resistance exercise interventions (IST and/or SET), while thirteen genes showed changes only in the CON group. Notably, pro-inflammatory genes (n = 90 genes) were most affected in the LPS-stimulated PBMCs after an exercise intervention, with the highest number of genes (n = 29 genes) showing altered expression patterns after the IST program. Furthermore, significant differences in gene expression were observed between the IST and SET groups in the LPS-stimulated PBMCs. Compared to the findings reported by Liberman et al. [12] our exploratory sub-study identified a greater number of genes affected by exercise in in vitro LPS-stimulated PBMCs, including both pro-inflammatory and anti-inflammatory genes, as well as genes with roles in inflammation that have yet to be fully determined. Moreover, the gene expression patterns differed following an in vitro LPS stimulation after a three-month resistance exercise intervention in older women.

### 4.1. Exercise-Induced Effects

The higher fold-changes observed after exercise-induced upregulation following LPS stimulation suggest that the upregulation reactions are more pronounced compared to the downregulation reactions, which generally exhibited lower fold-changes. The IST group showed a greater overall number of affected genes after the intervention. Additionally, both pro- and anti-inflammatory genes were more affected, which can reflect a heightened response to infection and clearance in this group.

#### 4.1.1. Pattern Recognition Receptors (PRRs)

Exercise, especially resistance exercise, has anti-inflammatory effects, which reduces TLR4-mediated inflammation through various mechanisms. Exercise leads to a decrease in TLR4 ligands and a higher release of intracellular inhibitors of the TLR4 cascade, as well as an increase in anti-inflammatory cytokines. Regular exercise training reduces the TLR4 response in PBMCs [9]. The results of this current exploratory sub-study show that TLR4 and TLR7 were both upregulated in the IST group and exhibited an attenuated effect after the intervention in response to in vitro LPS stimulation. Additionally, the RLR pathway was upregulated in the IST group. TLR4, TLR7, and CD14 showed no significant expression in the unstimulated condition [12], indicating a better immune response in the IST group. Olesen et al., 2015 [17] investigated the effects of training status on acute inflammation induced by LPS in young adults. However, no TLR4 mRNA expression could be detected in the PBMCs of the trained or untrained participants, which is consistent with the findings of Liberman et al. [12]. Fernandez-Gonzalo et al. [18] investigated the acute effects of a six-week resistance exercise program on the TLR4 signaling pathways in the PBMCs of young adults. In the trained condition, acute exercise elicited increased TLR4 gene expression in the control group, whereas no effect was observed in the trained group. These results also align with those of Liberman et al. [12], although the latter investigated basal effects in older adults, contrasting with the acute effects in young adults studied by Fernandez-Gonzalo et al. [18].

#### 4.1.2. Interleukins and Interferons

The pleiotropic interleukin IL6 was upregulated only in the CON group in the unstimulated condition [12], in contrast to an exercise-induced upregulation in all three intervention groups after LPS stimulation. Moreover, the exercise-induced effects of the IST group were attenuated, in contrast to an enhanced effect induced by the SET and CON groups. IL6R showed no significant changes in any of the three groups in the stimulated condition. We hypothesize that a negative feedback loop occurs in the IST group, where PBMCs produce less IL6 due to the higher overall concentration of IL6 in the circulation induced by the anti-inflammatory effects of high-intensity exercise. These results indicate that IL6 levels are influenced by LPS, reflecting improved immune regulation.

An exercise-induced attenuated effect regarding upregulation was observed for the anti-inflammatory gene IL10 in the IST group. In the unstimulated condition, no significant changes in IL10 gene expression were observed [12]. In contrast to Abbasi et al. [19], the current study utilized an LPS incubation period of 24 h to allow for the detection of almost all cytokines related to the immune response. IL10 production started three to five hours after exposure to LPS and was secondary to the pro-inflammatory release [19].

Various pro-inflammatory interleukins (IL1A, IL1B, IL3, and IL17A) and anti-inflammatory interleukins (IL2, IL1R2, IL9, and IL13) exhibited significant changes in gene expression patterns in both the stimulated (as presented in our study) and unstimulated conditions [12]. Different exercise-induced changes were observed in the pro-inflammatory interleukins across the three intervention groups, indicating that varying intensities of resistance exercise significantly affect the expression of inflammation-related genes. LPS-induced IL17A gene expression showed contradictory results compared to the unstimulated condition [12], likely due to the response to infection. In the IST group, a change in exercise-induced gene expression from down to upregulation was observed for IL2, leading to IL2 activation and contributing to the stimulation and proliferation of T cells. These findings indicate that in trained older adults undergoing an intensive strength training program, an immune response is elicited in vitro to LPS, unlike in the stretching CON group. LPS-induced IL13 gene expression showed an exercise-induced enhanced effect in upregulation in the SET group, contrasting with downregulation in the unstimulated condition in the SET and CON groups [12]. These findings suggest that IL13 changes in reaction to in vitro LPS stimulation; however, future research is necessary to unravel the underlying mechanisms. LPS-induced IFNg gene expression significantly increased in the SET group after the exercise intervention. Ulven et al. [20] showed no change in IL18 and IFNg gene expression after an acute aerobic exercise bout, which is comparable to the results of Liberman et al. [12] after a resistance exercise intervention. These findings highlight the complexity of the interleukin and interferon pathways in response to exercise and immune challenges.

#### 4.1.3. Chemokines and Tumor Necrosis Family

The gene expression patterns of several chemokines, including CCL19, CCL3, CX3CL1, CXCL10, CXCL11, and CXCR1, showed significant exercise-induced changes in gene expression in the stimulated condition. Chemokines are cytokines involved in cell migration [21]. This exercise-induced downregulation of CX3CL1 can reflect the inflammatory response, as CX3CL1 is involved in monocyte attraction and migration to inflammatory sites [22]. LPS-induced CCL3 gene expression showed an exercise-induced enhanced effect in upregulation only in the CON group. In the unstimulated condition, CCL3 was downregulated in the IST group [12]. With age, CCL3 or MIP1A can be reduced by NK cells [7]. LPS-induced downregulation of CXCL10 was significantly increased after the exercise intervention in all three groups, whereas in the unstimulated condition, only the CON and IST groups showed upregulation of CXCL10 gene expression [12]. These results are similar to those of Abbasi et al. [8], which showed that CXCL10 gene expression was strongly downregulated in LPS-stimulated whole blood cultures after exhaustive aerobic exercise. The LPS-induced change in CCL5 gene expression from up to downregulation was observed in the FT group, potentially reflecting lower egress of lymphocytes from blood to tissue and consequently a lower immune response of this group to infection due to ageing. The altered gene expression patterns after exercise intervention and stimulation with LPS across the three intervention groups demonstrate that different intensities of resistance exercise significantly influence the expression of genes associated with inflammation.

#### 4.1.4. Genes Related to the NF-KB Pathway

The results of our recent review by Salimans et al. showed that the NF-kB pathway is activated after a single acute bout of resistance exercise. The NF-kB pathway can be triggered by several stimuli, such as NOS2, TLRs, HSP, and cytokines such as IL1B and TNF. The expression of NOS2 and NOS3, related to the antioxidant defense system, is crucial in regulating inflammation [23].

This current exploratory sub-study revealed that NOS2 exhibited a significant exercise-induced change from down to upregulation in the stimulated condition, although only in the SET group. NOS2 is involved in the immune response and plays an important role in cellular signaling [23]. Additionally, TLR4 and TLR7 were both upregulated in the IST group and showed an attenuated effect after the intervention in response to in vitro LPS stimulation. This suggests a potential modulatory role of exercise on the TLR-mediated signaling pathways. Several HSPs showed exercise-induced effects after LPS stimulation. HSPs functions as molecular chaperones, produced in response to stressful situations to assist in stress resistance [23]. Specifically, HSPA1L demonstrated an exercise-induced enhanced effect on upregulation in the IST, while HSPA12A showed an exercise-induced enhanced effect on downregulation in the FT group. HSPA1A changed from being upregulated to downregulated in the IST after the exercise intervention following LPS stimulation, and HSPA1B showed an exercise-induced attenuated effect in upregulation in the IST. This contrasts with the findings in the unstimulated condition, in which HSPA1L, HSPA1A, and HSPA1B were upregulated in the SET group, with HSPA1A also being upregulated in the IST group [12]. Furthermore, several TNFs, as well as IL1B, showed exercise-induced changes after LPS stimulation, indicating that exercise modulates cytokine responses as well. These findings highlight the complexity of NF-kB pathway regulation in response to exercise and immune challenges.

Thus, the results of this explorative sub-study revealed that several genes that have the ability to trigger the NF-kB pathway exhibit exercise-induced effects after LPS stimulation. This suggests that exercise can modulate key inflammatory and stress response pathways, highlighting its mitigated effects on CLIP in older adults.

#### 4.1.5. Other Inflammation-Related Genes

CRP was only significantly changed in the stimulated condition. In both the IST and SET groups, CRP gene expression showed an exercise-induced alteration. However, in the IST group, CRP gene expression shifted from downregulation to upregulation, while in the SET group, it changed from upregulation to downregulation after in vitro LPS stimulation. Higher serum levels of CRP (between 5 and 10 mg/L) are associated with CLIP [1,24]. The exercise-induced alterations in CRP after LPS stimulation show that exercise elicits effects that mitigate the effects of CLIP.

The brain-related anti-inflammatory gene BDNF, a neurotrophic growth factor family member, showed an exercise-induced change from downregulation to upregulation in the IST group and an exercise-induced attenuated effect on downregulation in the SET group. No significant changes in gene expression were observed in the unstimulated condition [12]. The AMPK pathway was also downregulated in the SET group. BDNF gene expression typically occurs during inflammation and can elicit pro- and/or- anti-inflammatory effects [25]. Resistance exercise has been shown to enhance the circulatory levels of BDNF [26], and regular resistance exercise can increase BDNF levels [27]. Another brain-related gene, OLIG2, showed an exercise-induced change from downregulation to upregulation in all three groups after LPS stimulation. These results are in contrast with those of Abbasi et al. [8], which showed a downregulation of OLIG2, a regulator of oligodendrocytes. This suggest that OLIG2 gene expression is not restricted to the neural tissues [8].

### 4.2. Pathway Analyses Findings

Seven canonical pathways were upregulated in the IST group. Conversely, only one pathway related to inflammation, leukocyte extravasation signaling, was downregulated after the IST intervention. In the SET group, three pathways promoting inflammation—IL9, IL2, and JAK/Stat signaling—were upregulated, while five pathways were downregulated after the SET intervention. The most activated pathways in the LPS-stimulated cultures following three months of resistance exercise were associated with the T cell receptor signaling pathway, angiogenesis, and JAK-STAT signaling. Only the JAK/STAT signaling pathways aligned with the findings of Abbasi et al. [8] in LPS-stimulated whole blood cultures in response to an exhaustive aerobic exercise intervention. In the study by Liberman et al. [12], five pathways were comparable with the findings of our exploratory sub-study: TREM1 signaling, cytotoxic T lymphocyte-mediated apoptosis of target cells, IL2 signaling, VDR/RXR activation, and dopamine–DARPP32 feedback in cAMP signaling. Most of these pathways were affected in the CON in the unstimulated condition [12], in contrast to a mixed reaction in the LPS-stimulated condition in our exploratory sub-study. These mixed results suggest that the immune system targeted alternative pathways to mount an appropriate immune response to infection.

### 4.3. Strengths and Limitations

One limitation of the current exploratory sub-study is the inclusion of only older-aged women, which precluded the determination of sex-related differences in gene expression patterns observed by others [19,28]. Indeed, Abbasi et al. [19] showed sex-related differences between mRNA expression levels of IL10, IL1RA, and IL6, which could be linked to hormonal differences or sex-specific epigenetic landscapes that impact immune gene expression. The immune responses between men and women are different, in which men have a stronger monocyte-derived cytokine response than women [28]. A second limitation is that several intrinsic and extrinsic factors, such as genetic variability, medication use, and disease, can influence the expression of inflammation and immunosenescence-related genes in PBMCs. While efforts were made to carefully control for confounding factors such as medication intake and disease, individual genetic variability, lifestyle factors, and latent viral exposure (e.g., herpes and cytomegalovirus (CMV)) could still affect immune reaction and gene expression. In this explorative sub-study, an in vitro LPS stimulation is used. By challenging PBMCs derived from whole-blood cultures with the endotoxin LPS, the exercise-induced effects on cytokine production due to cellular components can be determined [8]. An in vitro challenge provides a good simulation; however, it only mimics an acute in vivo infection, lacking the whole-organism context, systemic interactions, and natural physiological conditions present in a living organism. Moreover, an active control group was used to correct for non-exercise intervention-related effects, such as environmental factors and factors related to travelling to the exercise facility. Despite these limitations, our explorative sub-study has several strengths. Firstly, the use of a randomized controlled trial design lends robustness to the findings. Secondly, the set of 407 genes related to inflammation, exercise, or ageing was carefully compiled through an extensive literature review. Thirdly, a sample size calculation was performed, ensuring adequate statistical power to detect exercise-induced gene expression changes. Moreover, RNA sequencing was employed to investigate the expression of the entire gene set (n = 407); a more comprehensive approach compared to most studies using reverse transcription polymerase chain reaction (RT-PCR) to investigate a limited number of genes. Furthermore, the participants included in the intervention program had similar physical activity levels and were not engaged in regular high-intensity exercise in the 6 months preceding the study, minimizing potential confounding effects of varying physical activity levels at baseline.

### 4.4. Future Perspectives

The results of this exploratory sub-study are very promising regarding the anti-inflammatory effects of resistance exercise. However, verification based on a confirmatory analysis involving also male participants is recommended.

Furthermore, the effects on protein expression need to be investigated. Post-transcriptional and post-translational modifications also influence the immune response, and these processes should be examined to provide a more comprehensive understanding of the exercise-induced changes. Moreover, exercise can induce changes in microRNAs (miRNAs) or epigenetic modifications, which can alter gene expression profiles.

To gain more insights into the effects of resistance exercise, it is also important to investigate detraining effects. This exploratory sub-study focused on the long-term intervention, but it would be interesting to investigate the effects of an acute bouts of exercise before and after the exercise intervention. This approach would provide valuable information regarding the differences in response between the trained and untrained conditions within the same group of participants.

As a final point, our results indicate that resistance exercise has the ability to influence the immune response. Although it has been shown that older persons engaging regularly in physical exercise are less susceptible to (infectious) diseases [4,29,30,31,32], it remains speculative whether the exercise-induced changes in the immune response that we observed in our exploratory sub-study are accompanied by substantial clinical impacts (e.g., less infection in exercising women, less cancer over time, etc.). A longer follow-up of a larger study population would allow us to verify whether resistance training leads to clinical benefits.

## 5. Conclusions

This research is the first of its kind to investigate the alterations in different genes and pathways based on the type of intervention. These findings are crucial for future research in this field and can serve as a steppingstone for further studies that will explore and expand upon these results. The results of this exploratory sub-study demonstrate that varying intensities of resistance exercise significantly impact the expression of genes linked to inflammation, immunosenescence, and ageing in PBMCs. Notably, three months of resistance exercise at both moderate and high intensities induced changes in the inflammatory stress response of PBMCs. However, distinct genes and pathways were influenced by each intensity level. Both intervention groups, IST and SET, showed differences in the expression of anti- and pro-inflammatory genes. The IST group exhibited a greater number of affected genes, both pro- and anti-inflammatory, which may indicate a stronger response to infection and a more robust mechanism for clearing the infection. Different genes and pathways are altered according to the intervention type. Based on these results, we conclude that a balanced exercise program alternating between moderate and high levels may be optimal for immune adaptations in older adults.

## Figures and Tables

**Table 1:** Baseline characteristics of the included participants in the RNAseq experiment.

	IST (n = 4)	SET (n = 5)	CON (n = 5)	*p*-Value
Weight (kg)	62.62 ± 5.41	69.43 ± 12.52	64.17 ± 4.60	0.47
Height (m)	1.63 ± 0.05	1.59 ± 0.06	1.54 ± 0.06	0.11
BMI (kg/m^2^)	23.34 ± 1.86	28.50 ± 3.72	27.44 ± 3.93	0.11
Age (years)	70.09 ± 4.64	72.13 ± 5.39	69.45 ± 2.62	0.61
MMSE (score: 0–30)	30.00 ± 0.00	27.2 ± 2.95	29.20 ± 0.84	0.10
Health categories (n)				
A	0	1	0	0.62
B	4	3	5	
C	0	1	0	
Charlson Index	0.50 ± 1.00	0.00 ± 0.00	0.40 ± 0.89	0.58
White blood cells (%)	0.99 (0.98–0.99)	0.99 (0.96–0.99)	0.99 (0.98–0.99)	0.87
Monocytes (%)	8.38 (7.91–10.08) †	5.45 (4.70–6.88)	7.81 (5.58–8.00)	0.02
Eosinophils (%)	1.43 (0.60–1.98)	2.77 (0.79–4.01)	3.99 (1.81–5.38)	0.19
Basophils (%)	0.37 (0.27–0.50)	0.58 (0.39–0.80)	0.79 (0.56–1.41)	0.05
Lymphocytes (%)	26.05 (21.65–38.02)	29.40 (26.90–33.20)	28.7 (24.20–43.62)	0.73
Energy expenditure (kilocalories/week)	6392.5 ± 2447.56	3520.9 ± 2509.21	6339.5 ± 1770.16	0.12
YPAS-ADS (Score 0–177)	44.75 ± 9.18	32.00 ± 15.26	38.00 ± 27.29	0.63
Medication use (n)	4.25 (0–10)	1.20 (0–4)	4.40 (1–7)	0.39
Hypertension (n)	0	1	2	0.35
Diabetes (n)	0	0	0	1.00

Baseline characteristics of the included women in the RNAseq experiment. One-way ANOVA, Fischer’s exact test, or Kruskal–Wallis test. † Significantly different from SET group (Mann–Whitney U test, *p* < 0.05). Results are presented as medians and ranges (min–max) or means ± standard deviations. BMI: body mass index, CON: flexibility training, control group, IST: intensive strength training, MMSE: mini mental state examination, SET: strength endurance training, YPAS-ADS: activity dimensions summary score of the Yale Physical Activity Survey.

**Table 2:** Changes in muscle strength index (MSI) after the 3-month intervention. (mean ± SE; * significant change *p* < 0.01).

	IST	SET	CON
Exercise compliance (%)	69.96 ± 19.47	93.41 ± 18.86	80.98 ± 7.31
MSI baseline	24.3 ± 6.1 kg	34.5 ± 8.7 kg	37.6 ± 14.5 kg
MSI 3 months	33.1 ± 8.5 kg	45.7 ± 7.9 kg	34.2 ± 4.9 kg
*p* value	0.005 *	0.002 *	0.54

**Table 3:** Heatmap of the significantly enriched pathways across the intervention groups. VDR/RXR: vitamin D receptor (VDR)/retinoid X receptor (RXR), RIG1: retinoic acid-inducible gene I, TWEAK: tumor necrosis factor-related weak inducer of apoptosis, CD: cluster of differentiation, IL: interleukin, JAK/STAT: janus kinase (JAK)/signal transducer and activator of transcription (STAT), GPCR: G protein-coupled receptors, AMPK: 5′ adenosine monophosphate-activated protein kinase, GNRH: gonadotropin releasing hormone, DARPP32: dopamine- and cAMP-regulated phosphoprotein, Mr 32 kD, cAMP: cyclic adenosine 3′,5′-monophosphate, TSP1: thrombospondin, TH17:T helper 17 cell, TREM1: triggering receptor expressed on myeloid cells 1. IST: intensive strength training, SET: strength endurance training, CON: flexibility training (control group).

Canonical Pathways	IST	SET	CON
Apelin cardiac fibroblast signaling pathway	2.00	0.00	−2.00
Cytotoxic T lymphocyte-mediated apoptosis of target cells	−2.12	2.83	0.00
Intrinsic prothrombin activation pathway	2.45	1.34	0.00
VDR/RXR activation	2.33	−0.33	1.00
Apelin liver signaling pathway	2.24	0.82	−0.45
Role of RIG1-like receptors in antiviral innate immunity	2.24	−0.45	0.45
TWEAK signaling	2.24	−0.45	0.45
CD27 signaling in lymphocytes	2.12	0.00	−0.71
Leukocyte extravasation signaling	−3.15	−0.73	1.70
IL-9 signaling	0.30	2.89	0.91
IL-2 signaling	−1.73	2.31	−0.58
Tumoricidal function of hepatic natural killer cells	−1.34	2.24	−0.45
JAK/stat signaling	−0.23	2.07	0.23
Endocannabinoid neuronal synapse pathway	−1.00	−2.00	0.00
GPCR-mediated nutrient sensing in enteroendocrine cells	−0.30	−2.11	−0.30
Neuropathic pain signaling in dorsal horn neurons	−0.30	−2.11	−0.30
AMPK signaling	−1.00	−2.12	0.00
Apelin cardiomyocyte signaling pathway	0.00	−2.14	−0.54
GNRH signaling	−1.16	−2.31	−1.73
Unfolded protein response	1.16	−2.31	−0.58
Dopamine-DARPP32 feedback in cAMP signaling	−0.33	−2.33	−0.33
Cardiac hypertrophy signaling	−1.96	−2.40	0.22
Inhibition of angiogenesis by TSP1	0.33	−3.00	−0.33
Crosstalk between dendritic cells and natural killer cells	1.89	1.67	2.65
Neuroinflammation signaling pathway	0.86	−0.61	2.32
Systemic lupus erythematosus In B cell signaling pathway	0.58	1.57	2.31
Th17 activation pathway	−0.20	1.40	2.20
T cell exhaustion signaling pathway	−0.58	0.96	2.12
TREM1 signaling	0.19	0.37	2.04

Red: Significantly activated pathways after the three-month exercise intervention in LPS-stimulated PBMCs (z-score ≥ −2). Blue: Significantly blunted pathways after three-month exercise intervention in LPS-stimulated PBMCs (z-score ≤ 2). White: Pathways that were not significantly affected after the three-month resistance exercise intervention in LPS-stimulated PBMCs (−2 ≥ z-score ≤ 2).

## Data Availability

The datasets and/or data analyses used during the current study are available from the corresponding author upon reasonable request.

## Supplementary Materials

The following supporting information can be downloaded at: https://www.mdpi.com/article/10.3390/cells13171416/s1, Table S1: Overview of the training and LPS-induced effects on the expression of genes that were significantly altered in at least one of the intervention groups, and for which the exact immune-modulatory role regarding exercise has not yet been described. Table S2: Overview of the genes showing a significant fold-change regarding the exercise-induced effects. Table S3: Overview of the training-induced effects on the expression of genes that significantly changed in at least one of the intervention groups, Figure S1: Pathways affected by 3 months of resistance exercise.
