# Supplementary material for: Inflammation-Related Genes Are Differentially Expressed in Lipopolysaccharide-Stimulated Peripheral Blood Mononuclear Cells after 3 Months of Resistance Training in Older Women
Source: Cells. 2024 Aug 25;13(17):1416. doi: 10.3390/cells13171416 (PMC11394400; doi:10.3390/cells13171416)
Supplement: Supplementary file 1 [file cells-13-01416-s001.zip › SALIMANS_LPS-RNAseq_Cells_Supplementary Figure S1.pdf]

**Canonical Pathways**	**IST**	**SET**	**CON**
Apelin Cardiac Fibroblast Signaling Pathway	2,00	0,00	-2,00
Cytotoxic T Lymphocyte-mediated Apoptosis of Target Cells	-2,12	2,83	0,00
Intrinsic Prothrombin Activation Pathway	2,45	1,34	0,00
VDR/RXR Activation	2,33	-0,33	1,00
Apelin Liver Signaling Pathway	2,24	0,82	-0,45
Role of RIG1-like Receptors in Antiviral Innate Immunity	2,24	-0,45	0,45
TWEAK Signaling	2,24	-0,45	0,45
CD27 Signaling in Lymphocytes	2,12	0,00	-0,71
Leukocyte Extravasation Signaling	-3,15	-0,73	1,70
IL-9 Signaling	0,30	2,89	0,91
IL-2 Signaling	-1,73	2,31	-0,58
Tumoricidal Function of Hepatic Natural Killer Cells	-1,34	2,24	-0,45
JAK/Stat Signaling	-0,23	2,07	0,23
Endocannabinoid Neuronal Synapse Pathway	-1,00	-2,00	0,00
GPCR-Mediated Nutrient Sensing in Enteroendocrine Cells	-0,30	-2,11	-0,30
Neuropathic Pain Signaling In Dorsal Horn Neurons	-0,30	-2,11	-0,30
AMPK Signaling	-1,00	-2,12	0,00
Apelin Cardiomyocyte Signaling Pathway	0,00	-2,14	-0,54
GNRH Signaling	-1,16	-2,31	-1,73
Unfolded protein response	1,16	-2,31	-0,58
Dopamine-DARPP32 Feedback in cAMP Signaling	-0,33	-2,33	-0,33
Cardiac Hypertrophy Signaling	-1,96	-2,40	0,22
Inhibition of Angiogenesis by TSP1	0,33	-3,00	-0,33
Crosstalk between Dendritic Cells and Natural Killer Cells	1,89	1,67	2,65
Neuroinflammation Signaling Pathway	0,86	-0,61	2,32
Systemic Lupus Erythematosus In B Cell Signaling Pathway	0,58	1,57	2,31
Th17 Activation Pathway	-0,20	1,40	2,20
T Cell Exhaustion Signaling Pathway	-0,58	0,96	2,12
TREM1 Signaling	0,19	0,37	2,04

Pathways altered in two intervention groups:
IST and CON group
Apelin Cardiac Fibroblast Signaling Pathway

IST and SET group
Cytotoxic T Lymphocyte-mediated Apoptosis of Target Cells

Pathways altered in IST group
Intrinsic Prothrombin Activation Pathway
VDR/RXR Activation
Apelin Liver Signaling Pathway
Role of RIG1-like Receptors in Antiviral Innate Immunity
TWEAK Signaling
CD27 Signaling in Lymphocytes
Leukocyte Extravasation Signaling

Pathways altered in SET group
IL-9 Signalingjak
IL-2 Signaling
Tumoricidal Function of Hepatic Natural Killer Cells
JAK/Stat Signaling
Endocannabinoid Neuronal Synapse Pathway
GPCR-Mediated Nutrient Sensing in Enteroendocrine Cells
Neuropathic Pain Signaling In Dorsal Horn Neurons
AMPK Signaling
Apelin Cardiomyocyte Signaling Pathway
GNRH Signaling
Unfolded protein response
Dopamine-DARPP32 Feedback in cAMP Signaling
Cardiac Hypertrophy Signaling
Inhibition of Angiogenesis by TSP1

Pathways altered in CON group
Crosstalk between Dendritic Cells and Natural Killer Cells
Neuroinflammation Signaling Pathway
Systemic Lupus Erythematosus In B Cell Signaling Pathway
Th17 Activation Pathway
T Cell Exhaustion Signaling Pathway
TREM1 Signaling

Pathways altered in two intervention groups:

Apelin Cardiac Fibroblast Signaling Pathway

IST group (z-score= 2.00)

Apelin Cardiac Fibroblast Signaling Pathway : LPS_FC_T2vsT0 : Expr Log Ratio

Apelin inhibits the activation of cardiac fibroblasts and their differentiation into myofibroblasts, thereby prevents formation of cardiac fibrosies which lead to heart failure.

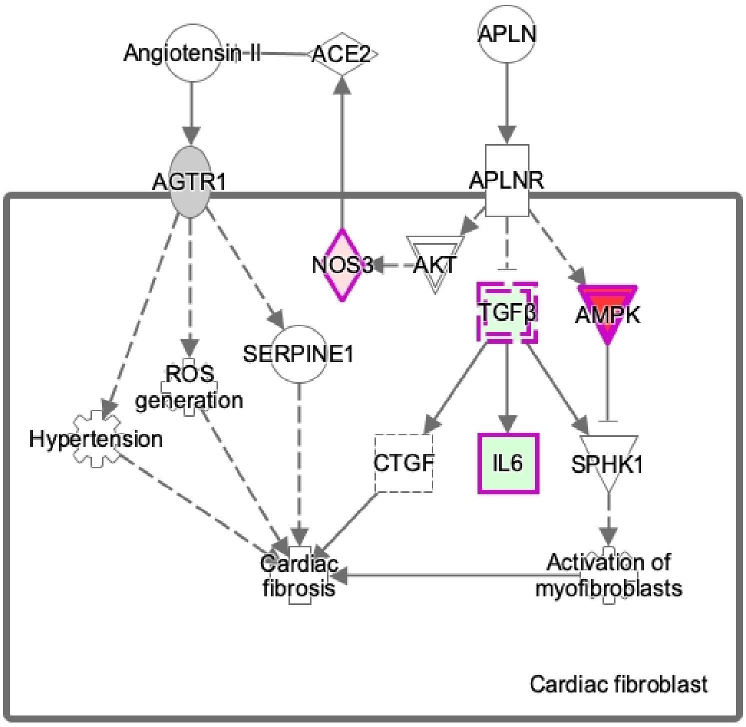

© 2000-2020 QIAGEN. All rights reserved.

Apelin Cardiac Fibroblast Signaling Pathway

CON group (z-score= -2.00)

Apelin Cardiac Fibroblast Signaling Pathway : LPS_FC_T2vsT0 : Expr Log Ratio

Apelin inhibits the activation of cardiac fibroblasts and their differentiation into myofibroblasts, thereby prevents formation of cardiac fibrosies which lead to heart failure.

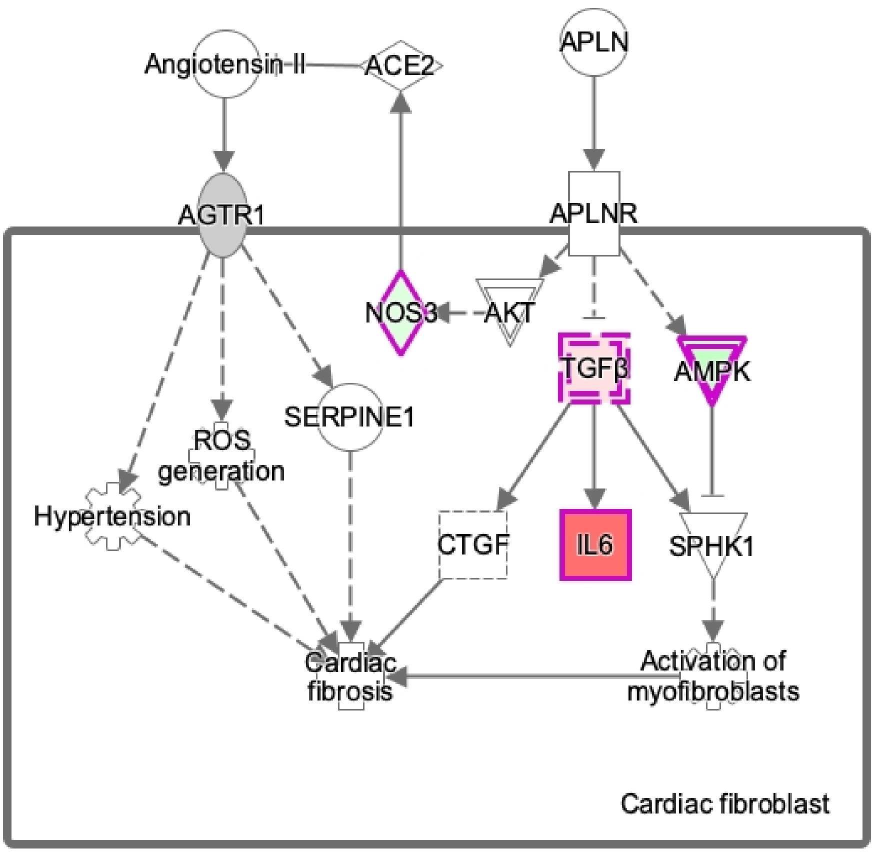

© 2000-2020 QIAGEN. All rights reserved.

Cytotoxic T Lymphocyte-mediated Apoptosis of Target Cells

IST group (z-score= -2.12)

Cytotoxic T Lymphocyte-mediated Apoptosis of Target Cells : LPS_FC_T2vsT0 : Expr Log Ratio

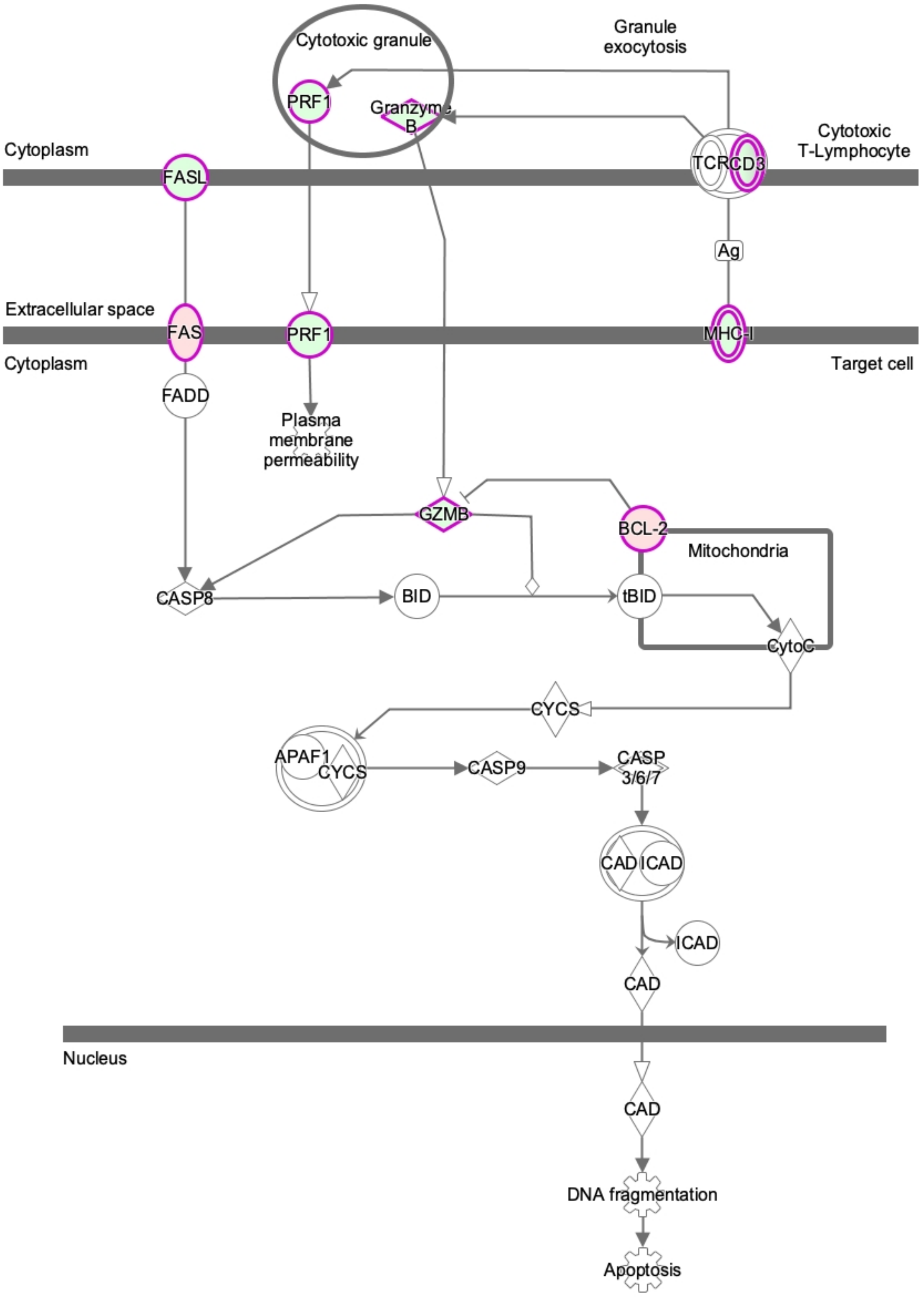

© 2000-2020 QIAGEN. All rights reserved.

Cytotoxic T Lymphocyte-mediated Apoptosis of Target Cells
SET group (z-score= 2.83)

Cytotoxic T Lymphocyte-mediated Apoptosis of Target Cells : LPS_FC_T2vsT0 : Expr Log Ratio

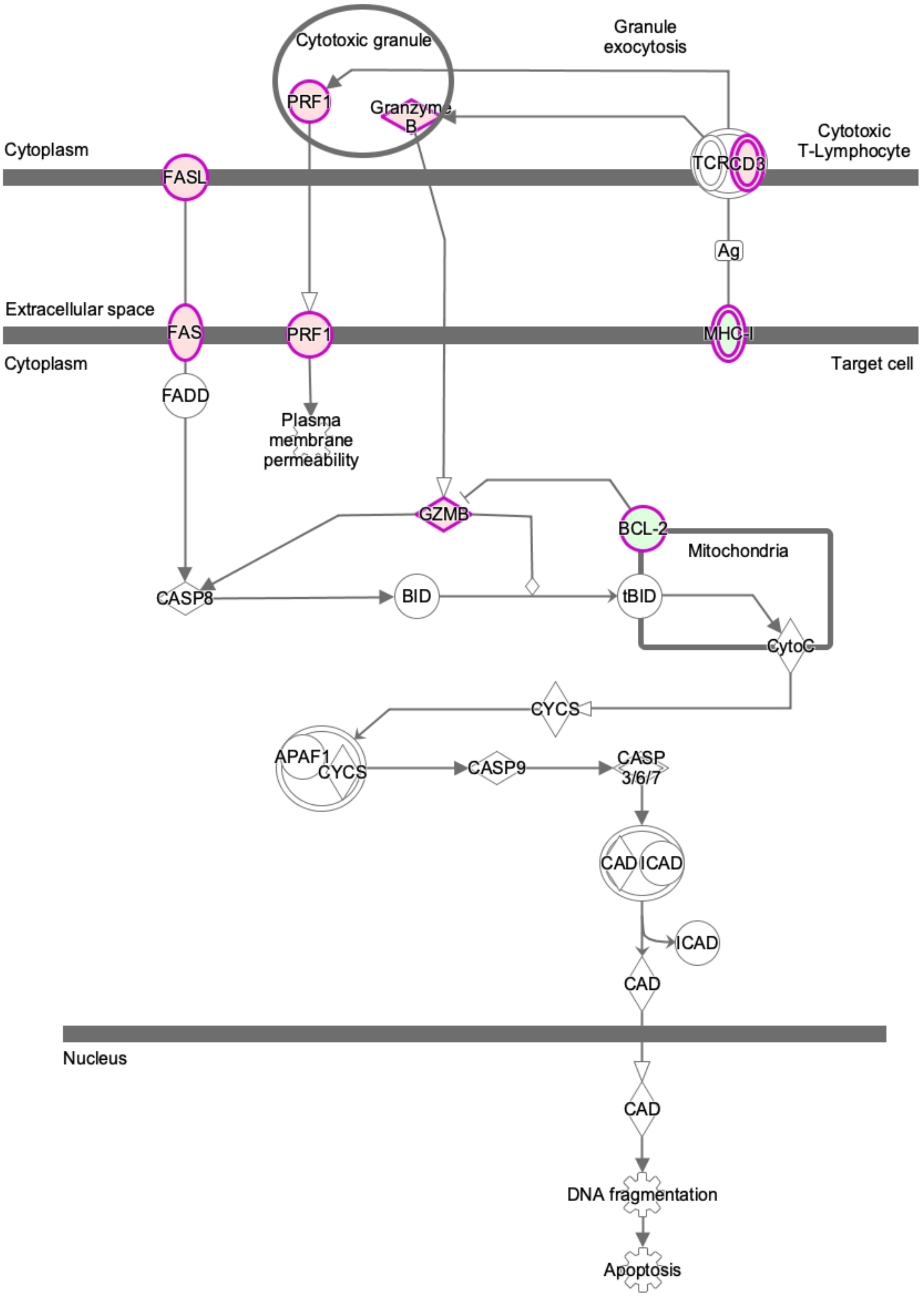

© 2000-2020 QIAGEN. All rights reserved.

Pathways altered in IST group

Intrinsic Prothrombin Activation Pathway *(z-score= 2.45)*

Intrinsic Prothrombin Activation Pathway : LPS_FC_T2vsT0 : Expr Log Ratio

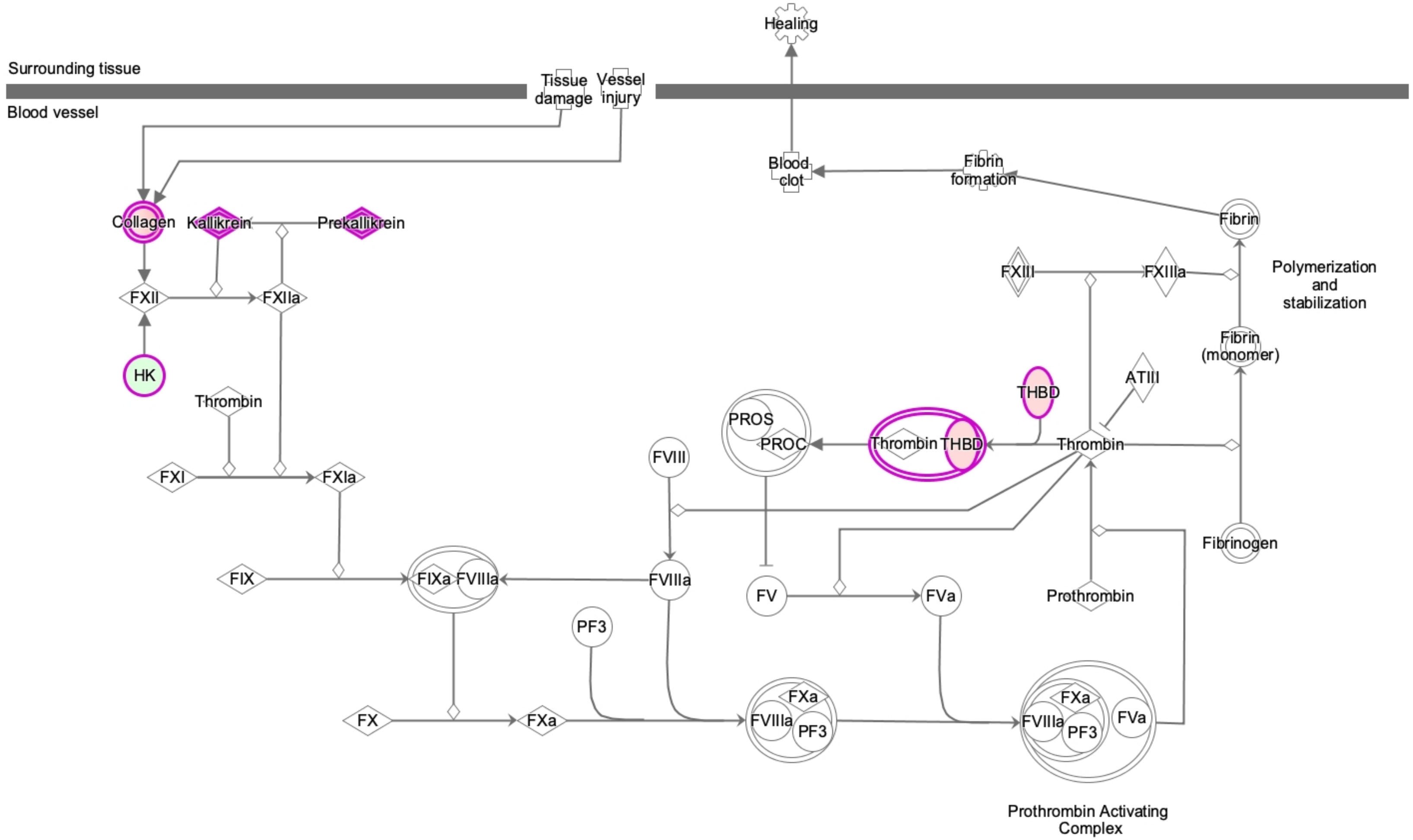

© 2000-2020 QIAGEN. All rights reserved.

VDR/RXR Activation *(z-score= 2.33)*

VDR/RXR Activation : LPS_FC_T2vsT0 : Expr Log Ratio

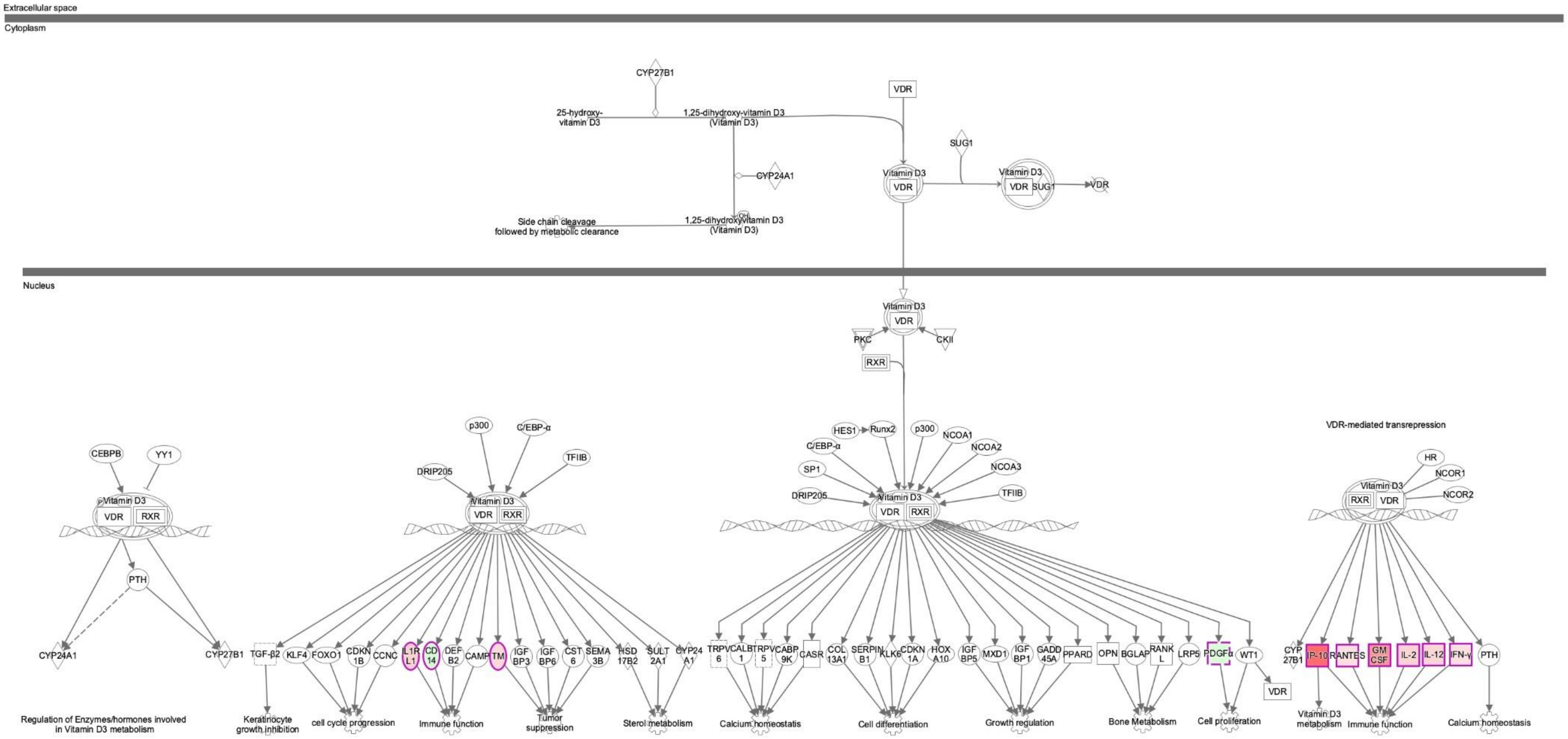

© 2000-2020 QIAGEN. All rights reserved.

Apelin Liver Signaling Pathway *(z-score= 2.24)*

Apelin Liver Signaling Pathway : LPS_FC_T2vsT0 : Expr Log Ratio

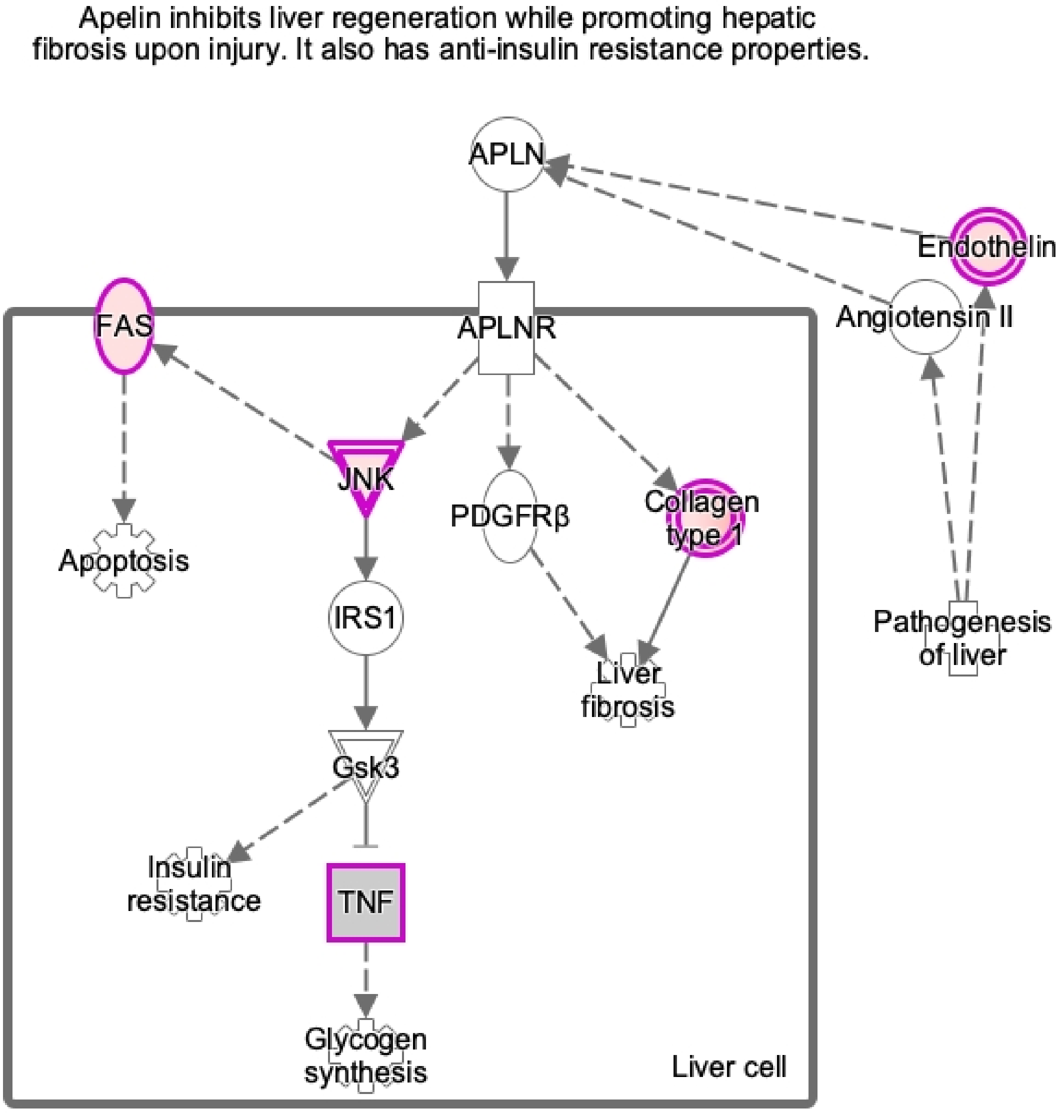

© 2000-2020 QIAGEN. All rights reserved.

Role of RIG1-like Receptors in Antiviral Innate Immunity ((*z-score*= 2.24)

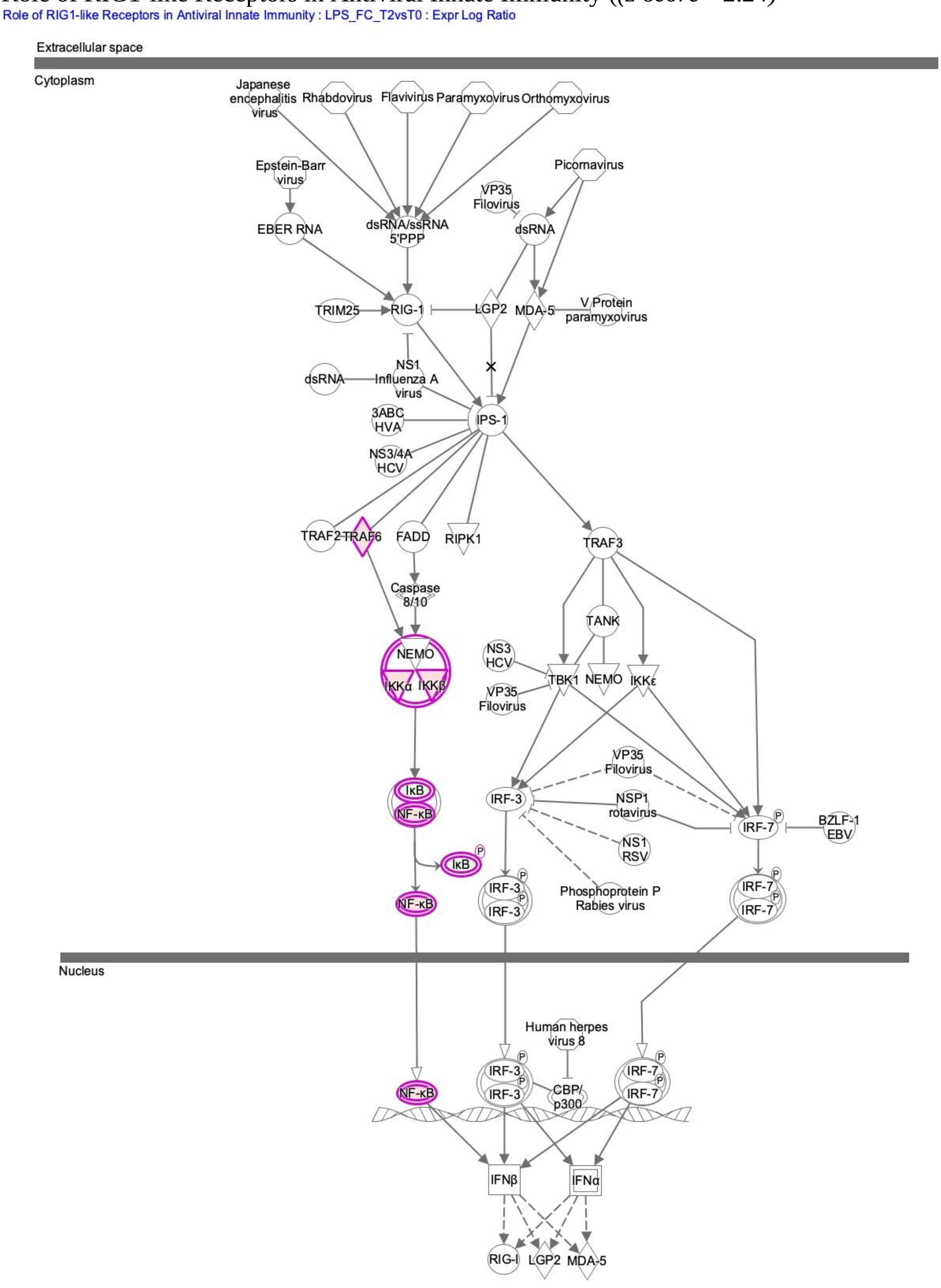

© 2000-2020 QIAGEN. All rights reserved.

TWEAK Signaling *(z-score= 2.24)*

TWEAK Signaling : LPS_FC_T2vsT0 : Expr Log Ratio

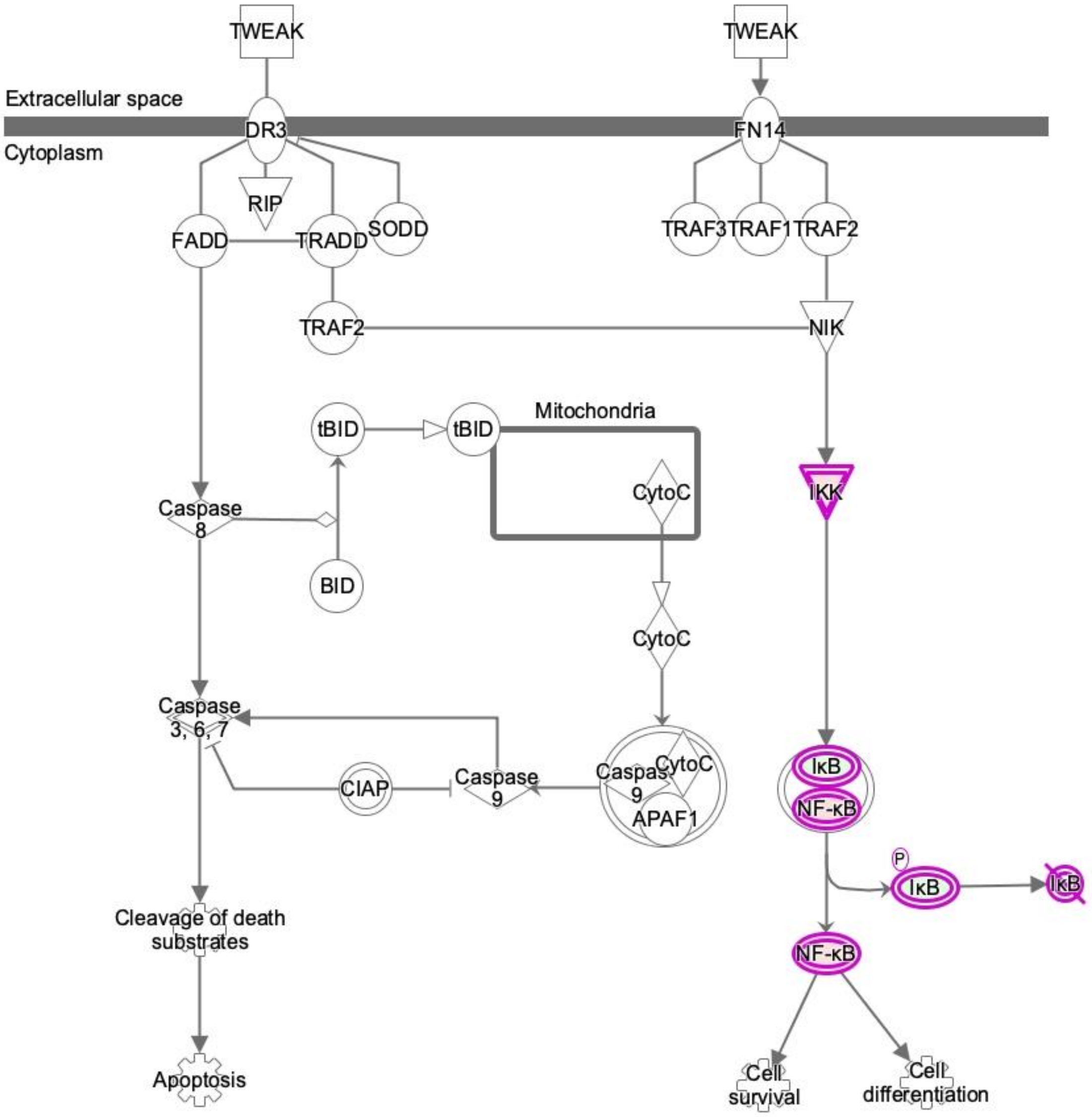

© 2000-2020 QIAGEN. All rights reserved.

CD27 Signaling in Lymphocytes *(z-score= 2.12)*

CD27 Signaling in Lymphocytes : LPS_FC_T2vsT0 : Expr Log Ratio

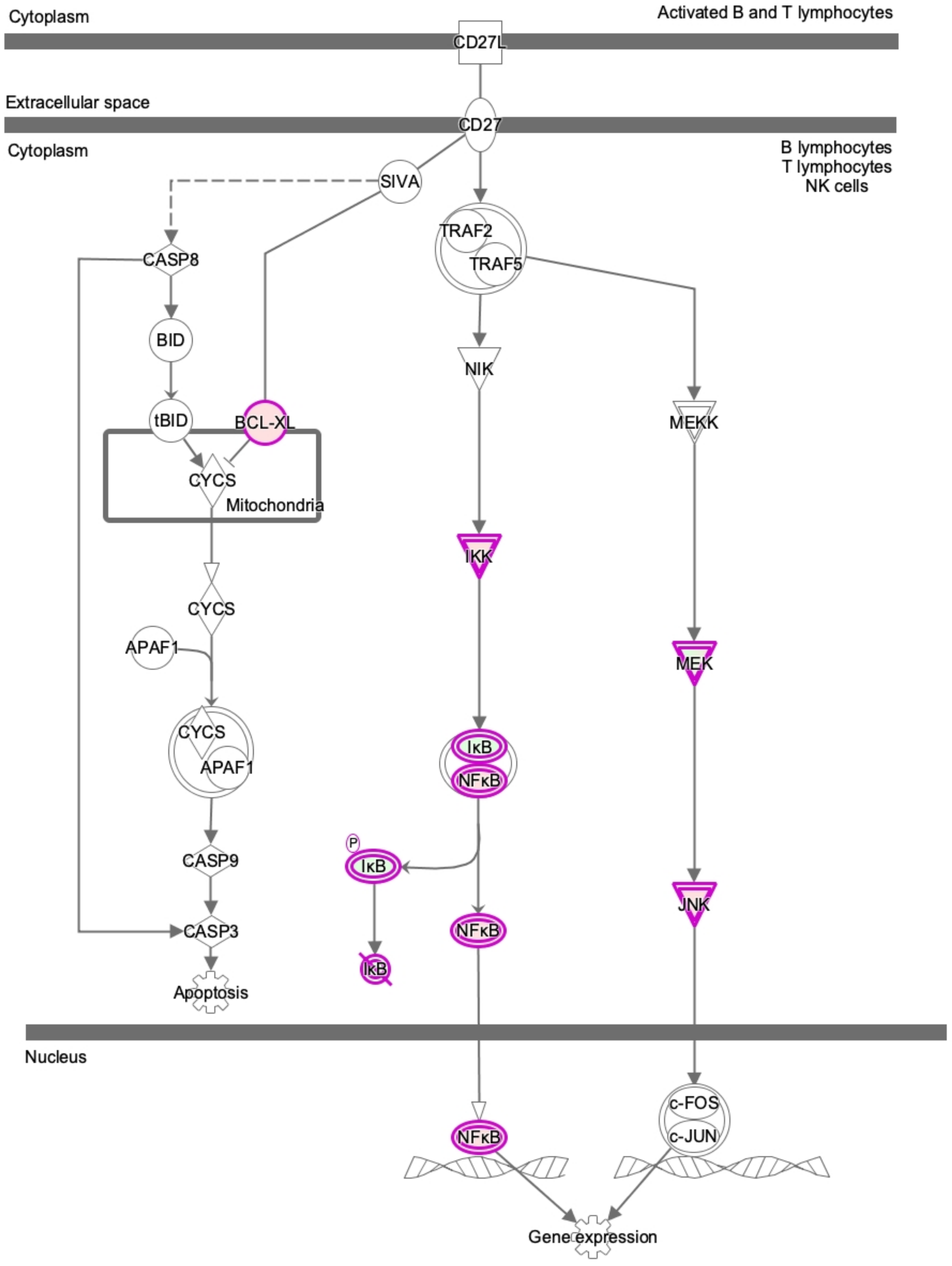

© 2000-2020 QIAGEN. All rights reserved.

Leukocyte Extravasation Signaling *(z-score= -3.15)*

Leukocyte Extravasation Signaling : LPS_FC_T2vsT0 : Expr Log Ratio

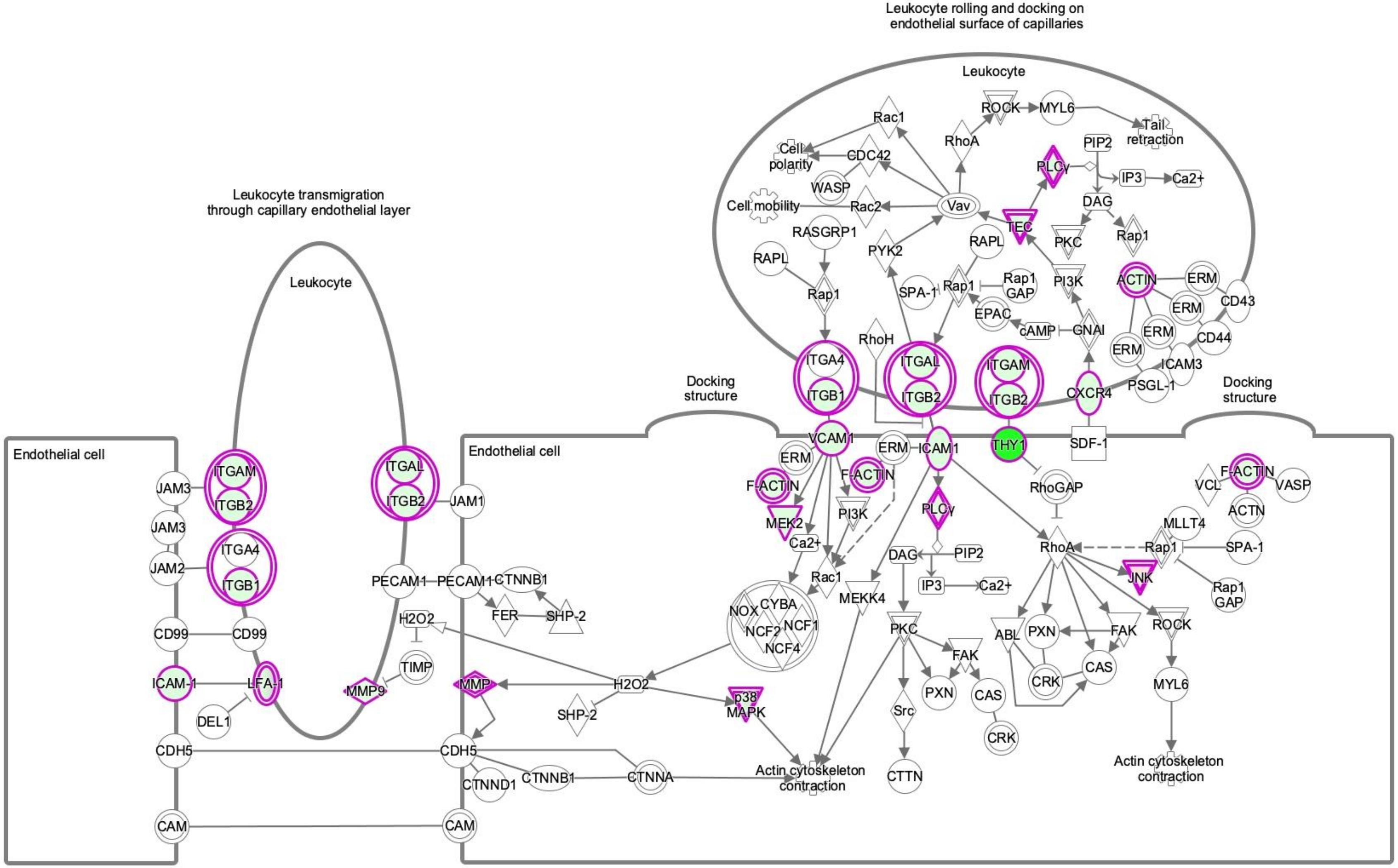

© 2000-2020 QIAGEN. All rights reserved.

Pathways altered in SET group

IL-9 Signaling ((*z-score= 2.89)*

IL-9 Signaling : LPS_FC_T2vsT0 : Expr Log Ratio

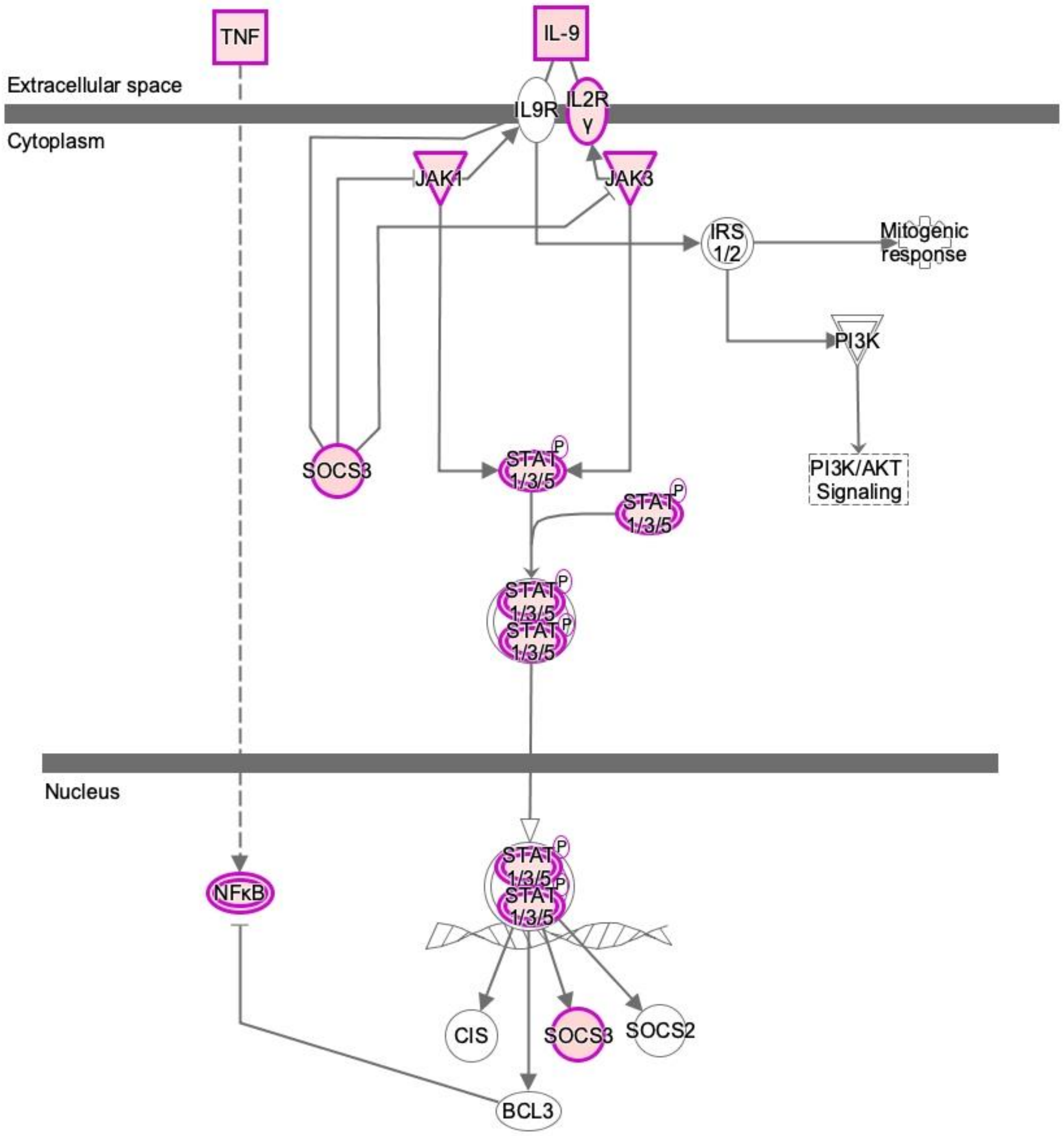

© 2000-2020 QIAGEN. All rights reserved.

IL-2 Signaling ((*z-score*= *2.31)*

IL-2 Signaling : LPS_FC_T2vsT0 : Expr Log Ratio

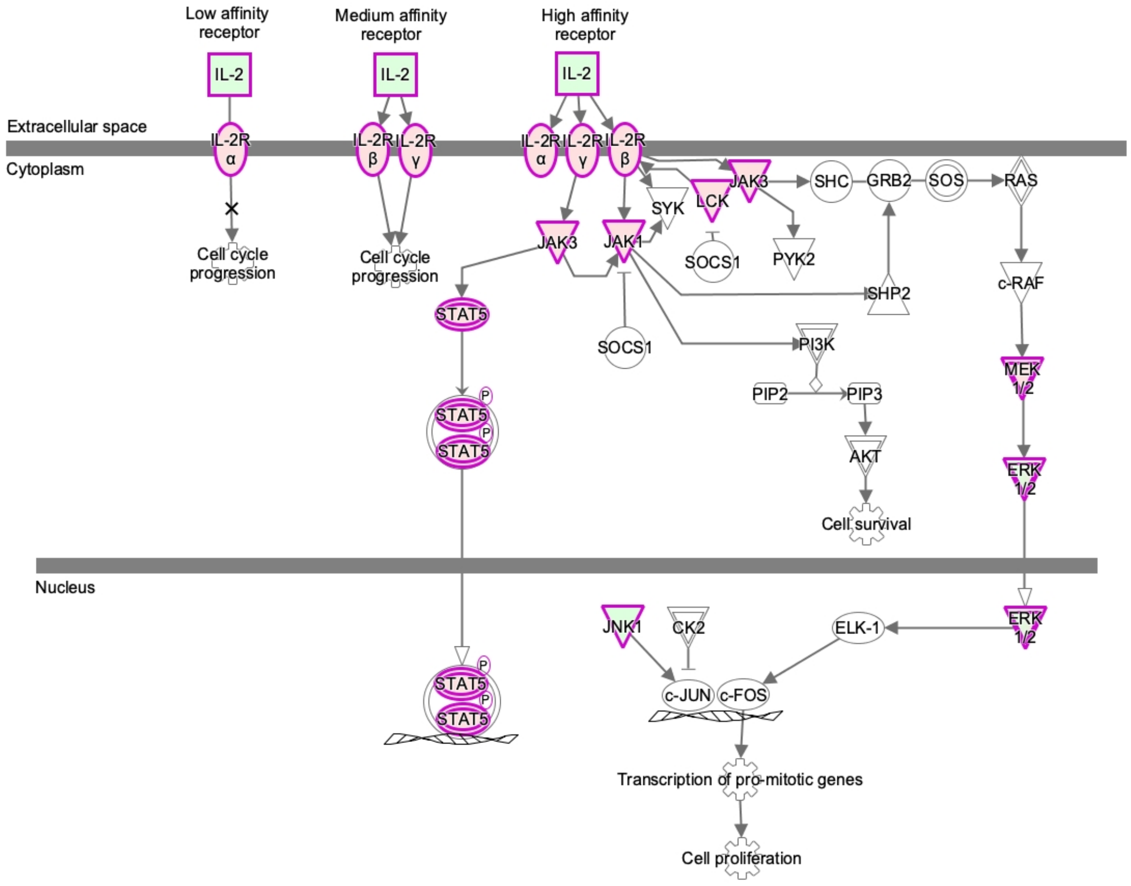

© 2000-2020 QIAGEN. All rights reserved.

Tumoricidal Function of Hepatic Natural Killer Cells *(z-score= 2.24)*

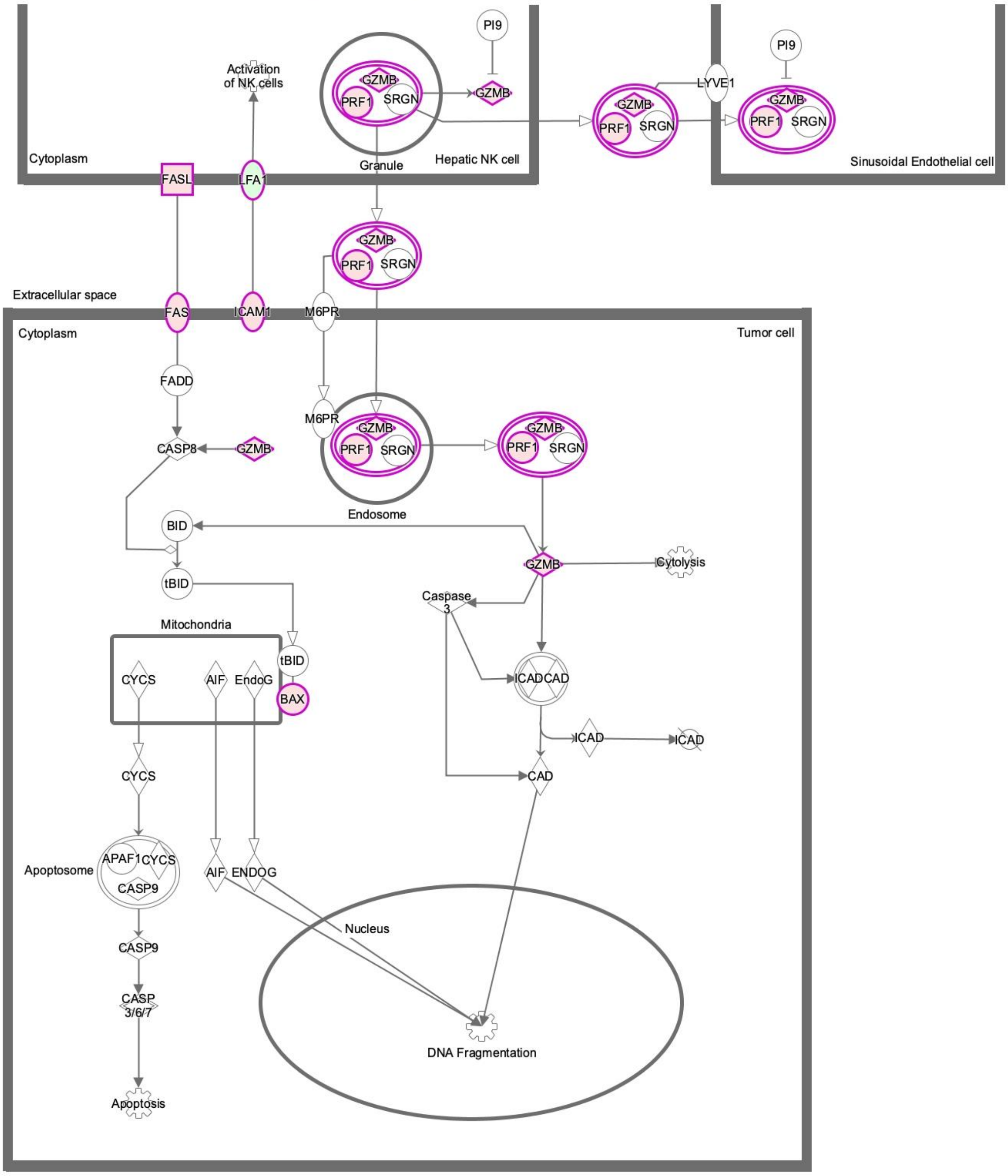

© 2000-2020 QIAGEN. All rights reserved.

JAK/Stat Signaling *(z-score= 2.07)*

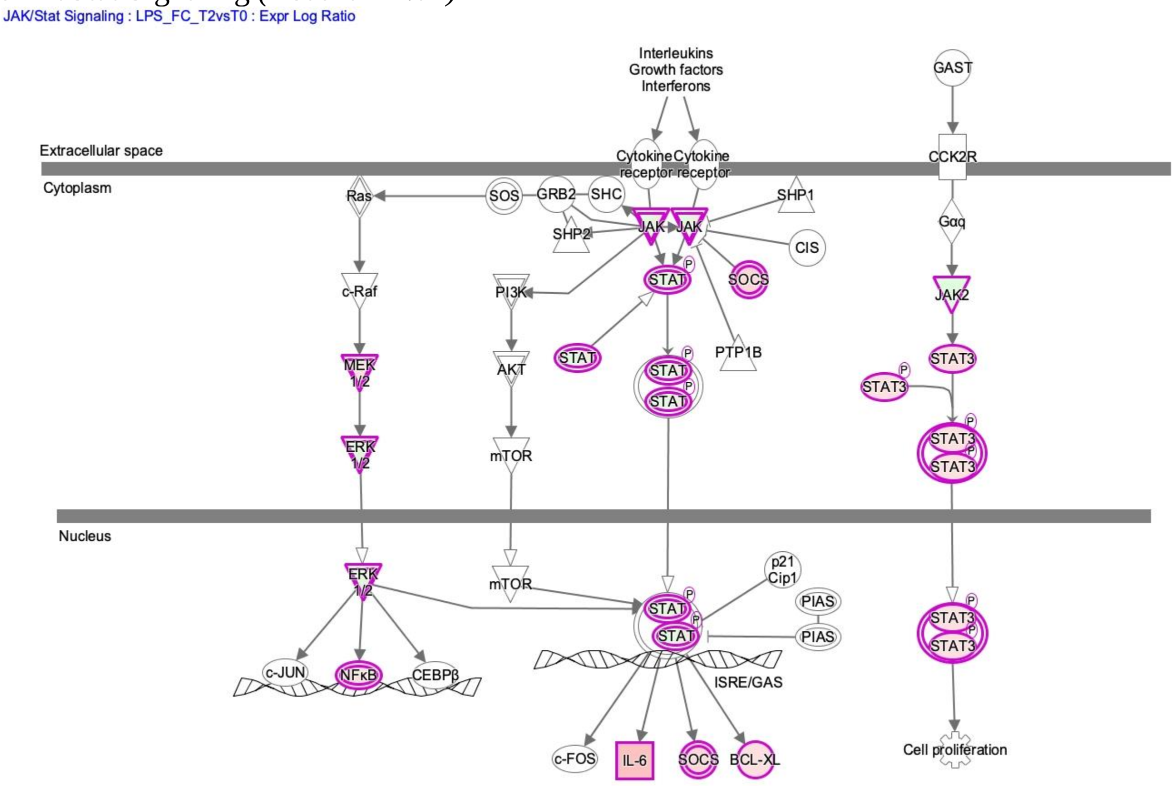

© 2000-2020 QIAGEN. All rights reserved.

Endocannabinoid Neuronal Synapse Pathway *(z-score= -2.00)*

Endocannabinoid Neuronal Synapse Pathway : LPS_FC_T2vsT0 : Expr Log Ratio

Endocannabinoids regulate synaptic function through retrograde signaling, autocrine signaling, and also indirectly by activating astrocytic receptors.

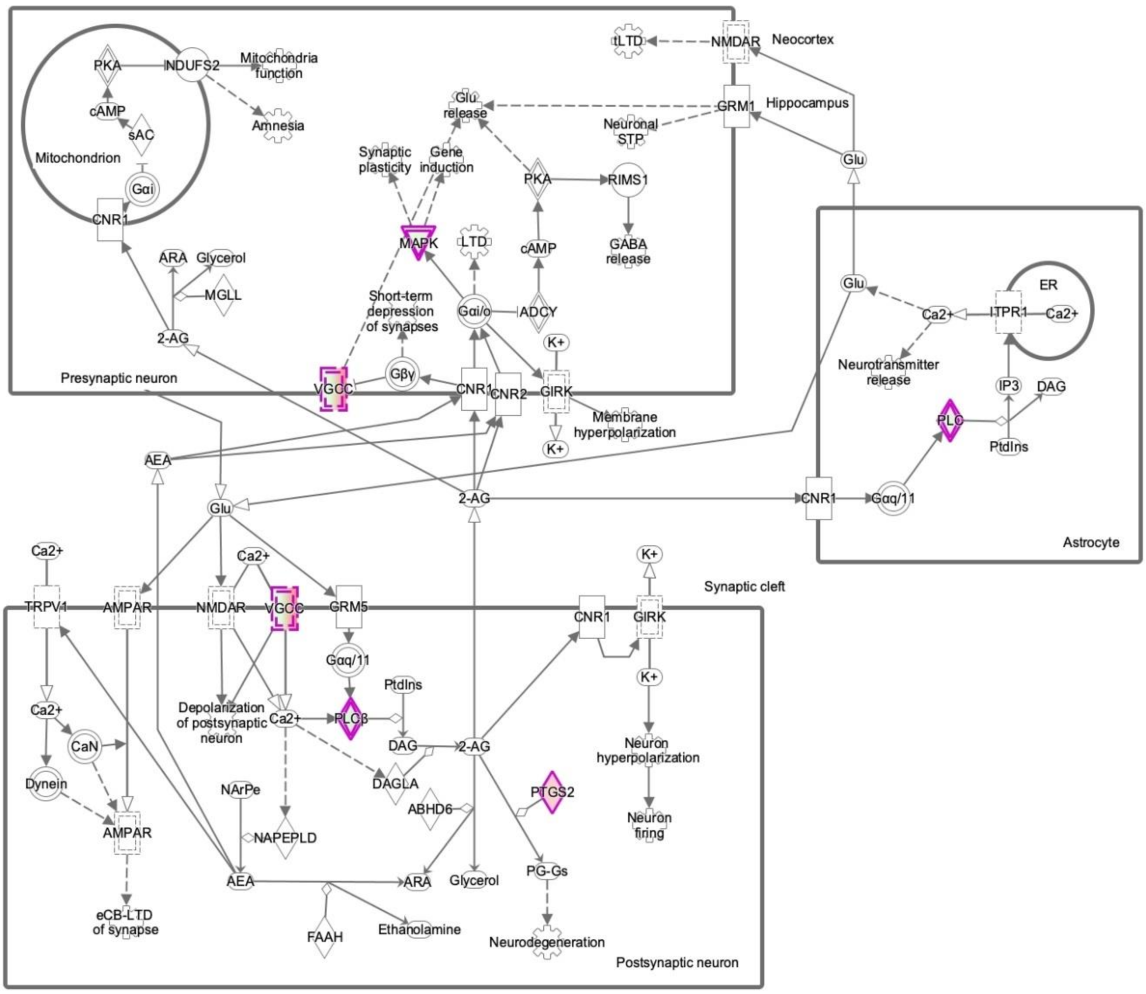

© 2000-2020 QIAGEN. All rights reserved.

GPCR-Mediated Nutrient Sensing in Enteroendocrine Cells *(z-score= -2.11)*

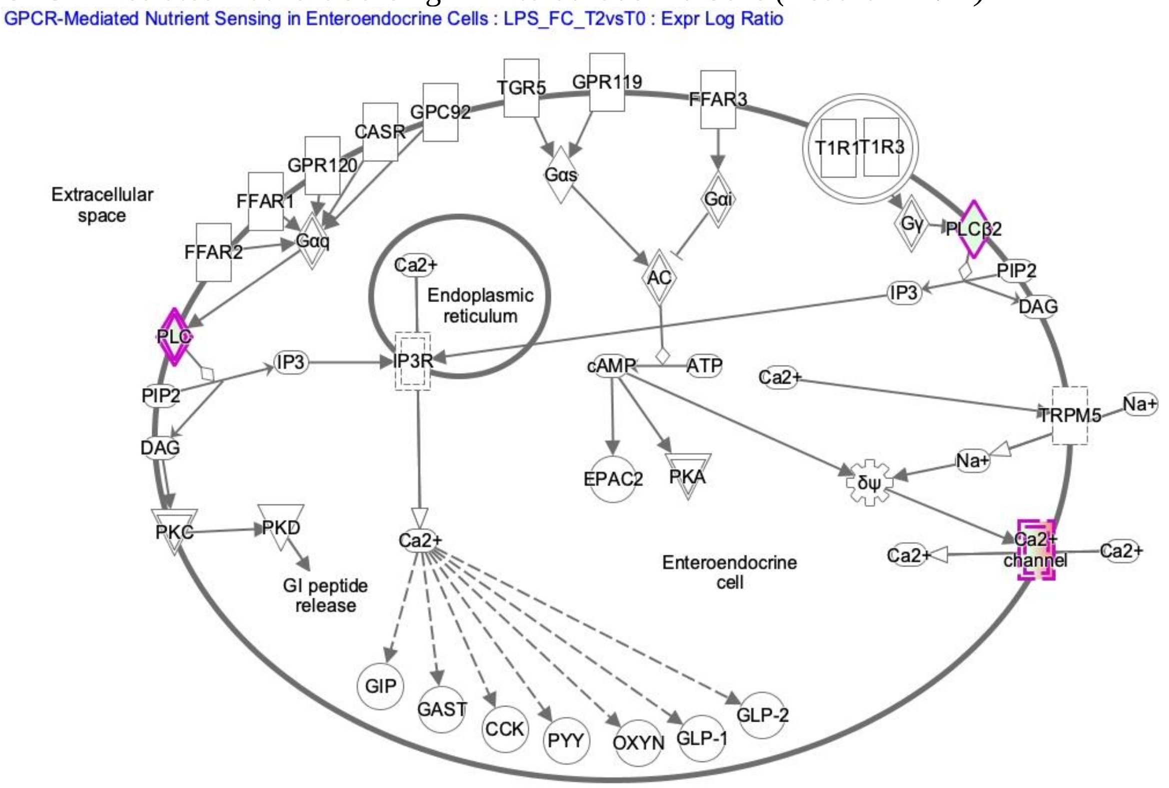

© 2000-2020 QIAGEN. All rights reserved.

Neuropathic Pain Signaling In Dorsal Horn Neurons (*z-score= -2.11)*

Neuropathic Pain Signaling In Dorsal Horn Neurons : LPS_FC_T2vsT0 : Expr Log Ratio

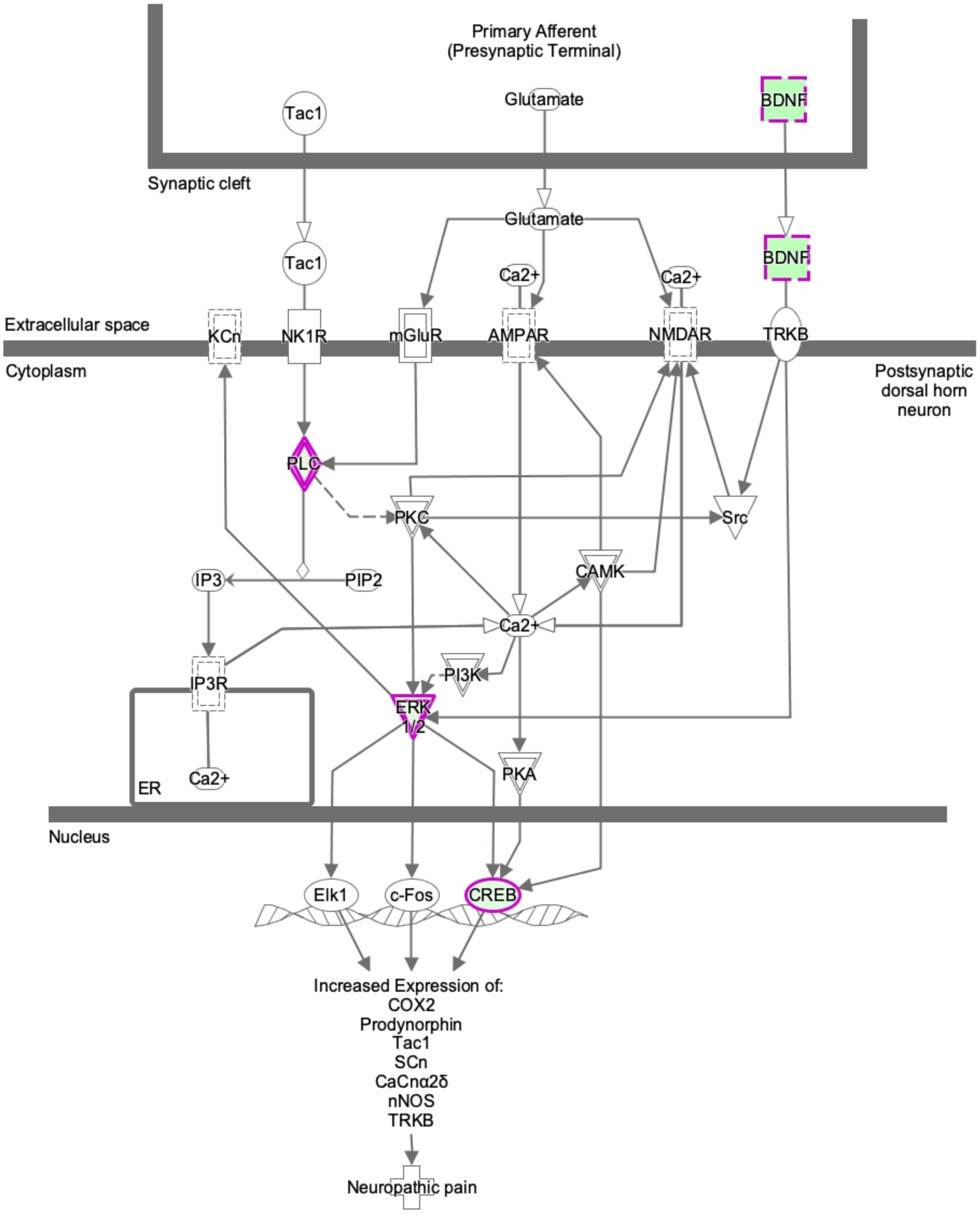

© 2000-2020 QIAGEN. All rights reserved.

AMPK Signaling ((*z-score*= *-2.12)*

AMPK Signaling : LPS_FC_T2vsT0 : Expr Log Ratio

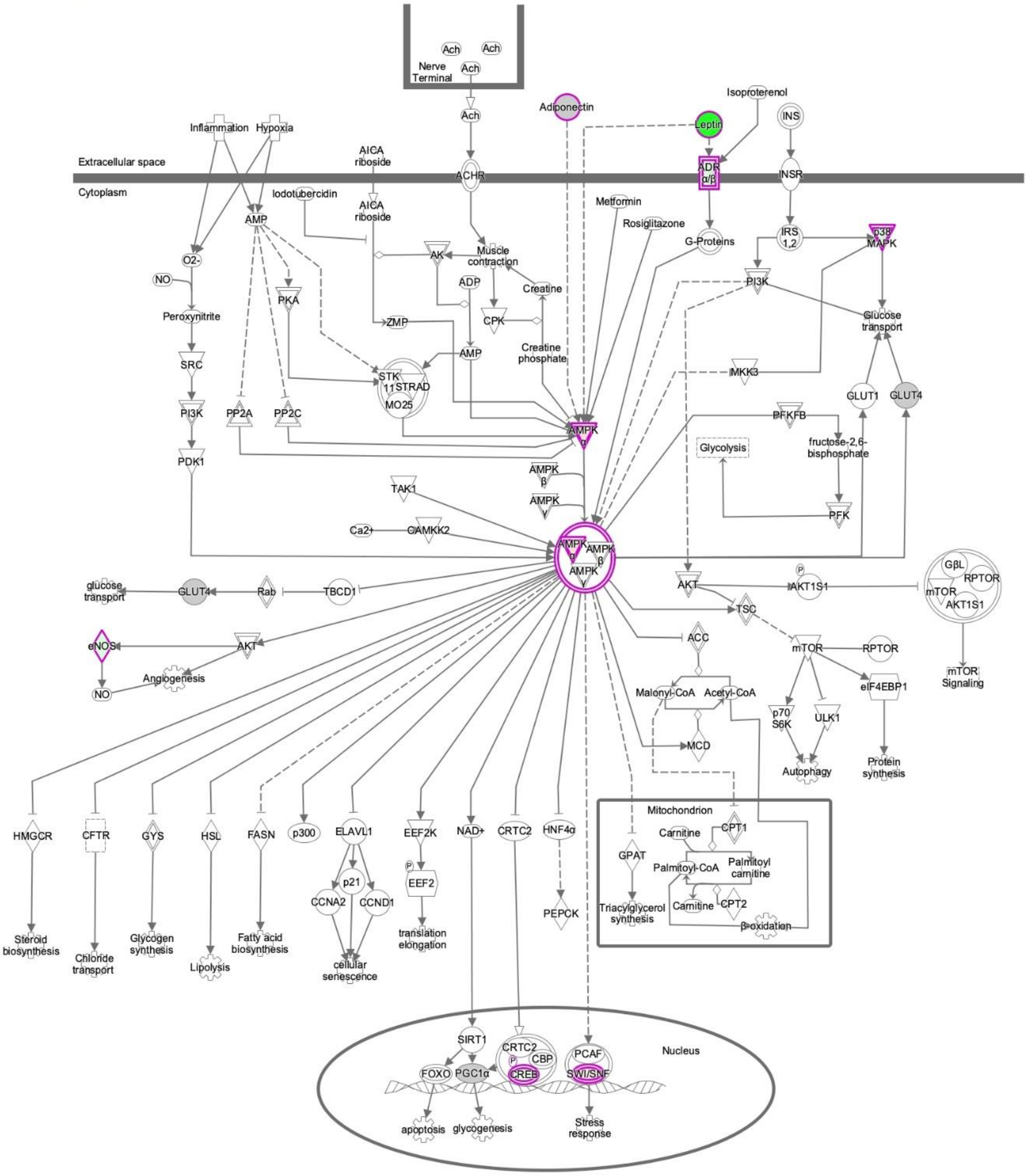

© 2000-2020 QIAGEN. All rights reserved.

Apelin Cardiomyocyte Signaling Pathway *(z-score= -2.14)*

Apelin Cardiomyocyte Signaling Pathway : LPS_FC_T2vsT0 : Expr Log Ratio

Apelin is an important regulator of cardiac contractility in cardiomyocytes
and also protects the heart against cardiac hypertrophy and ischemia-reperfusion injury.

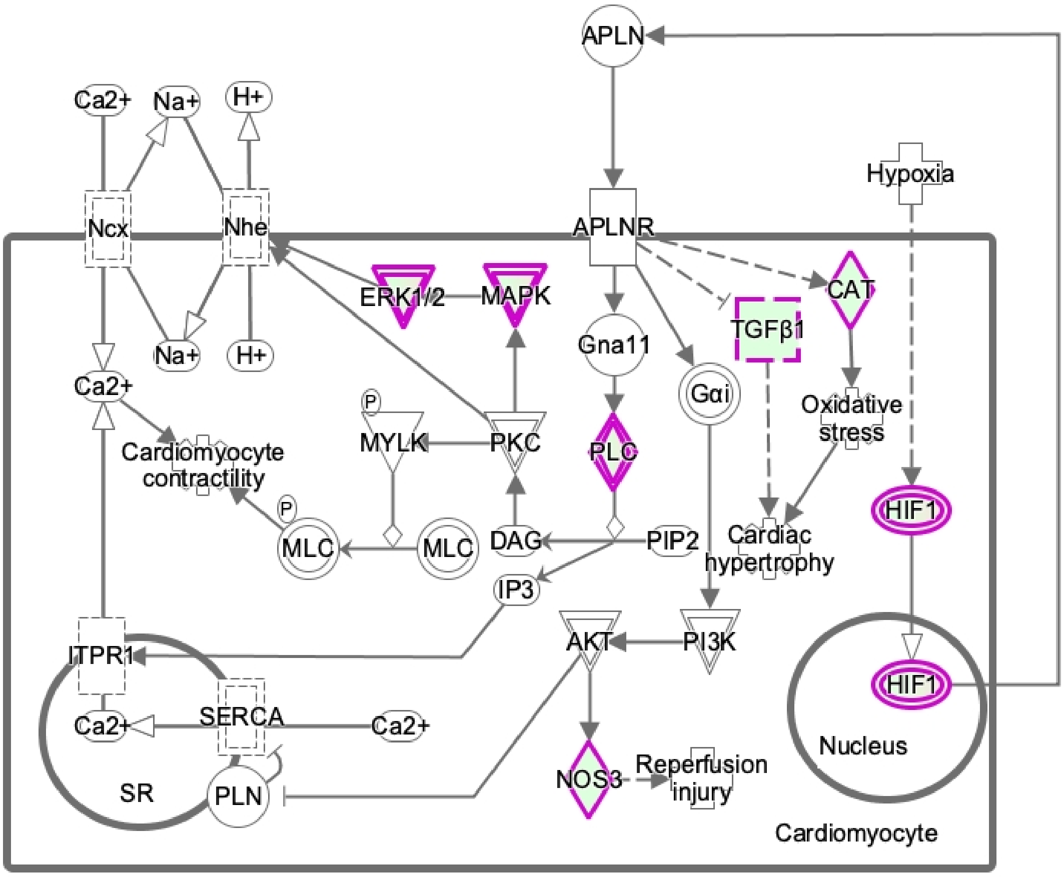

© 2000-2020 QIAGEN. All rights reserved.

GNRH Signaling *(z-score= -2.31)*

GNRH Signaling : LPS_FC_T2vsT0 : Expr Log Ratio

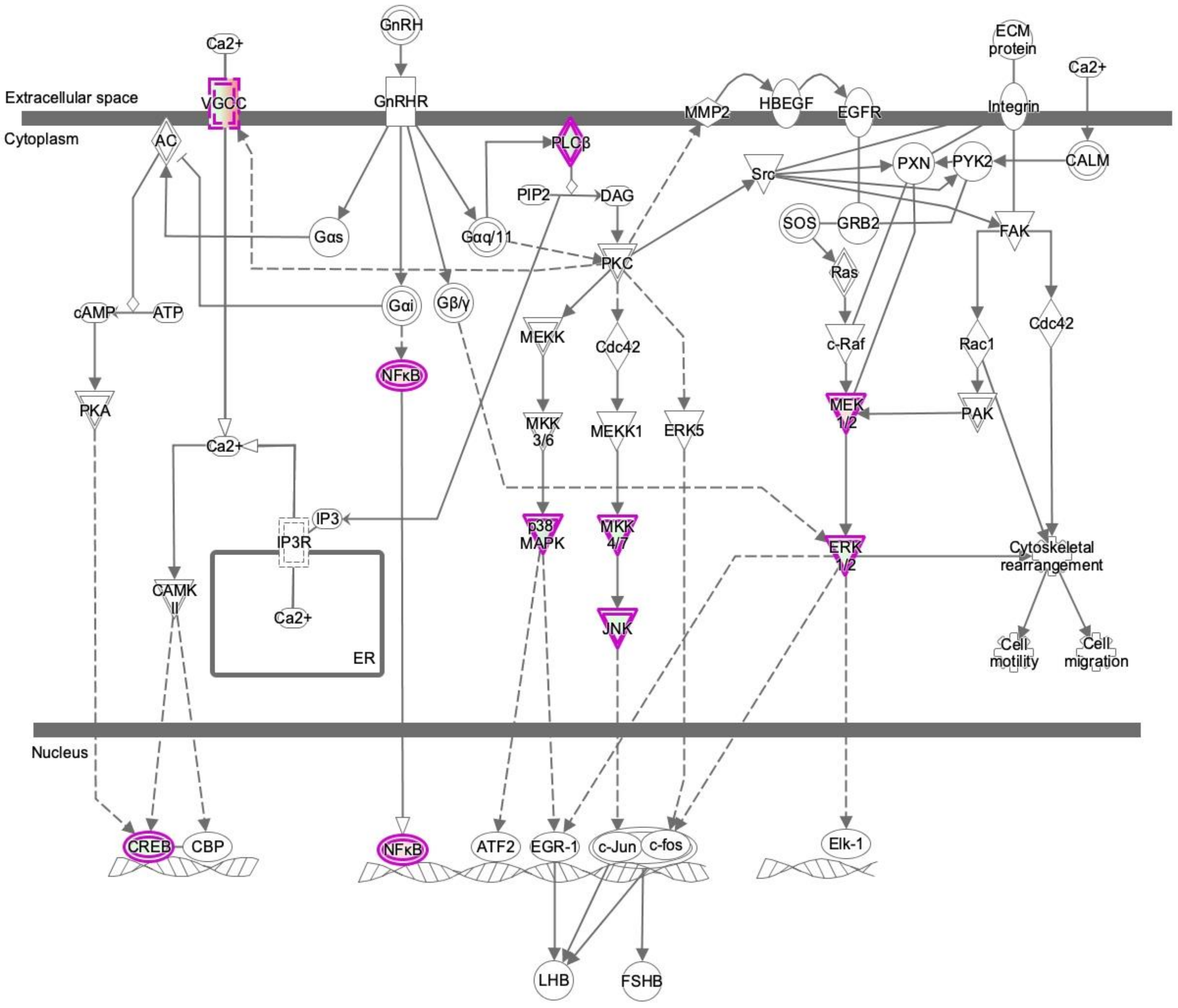

© 2000-2020 QIAGEN. All rights reserved.

Unfolded protein response *(z-score= -2.31)*

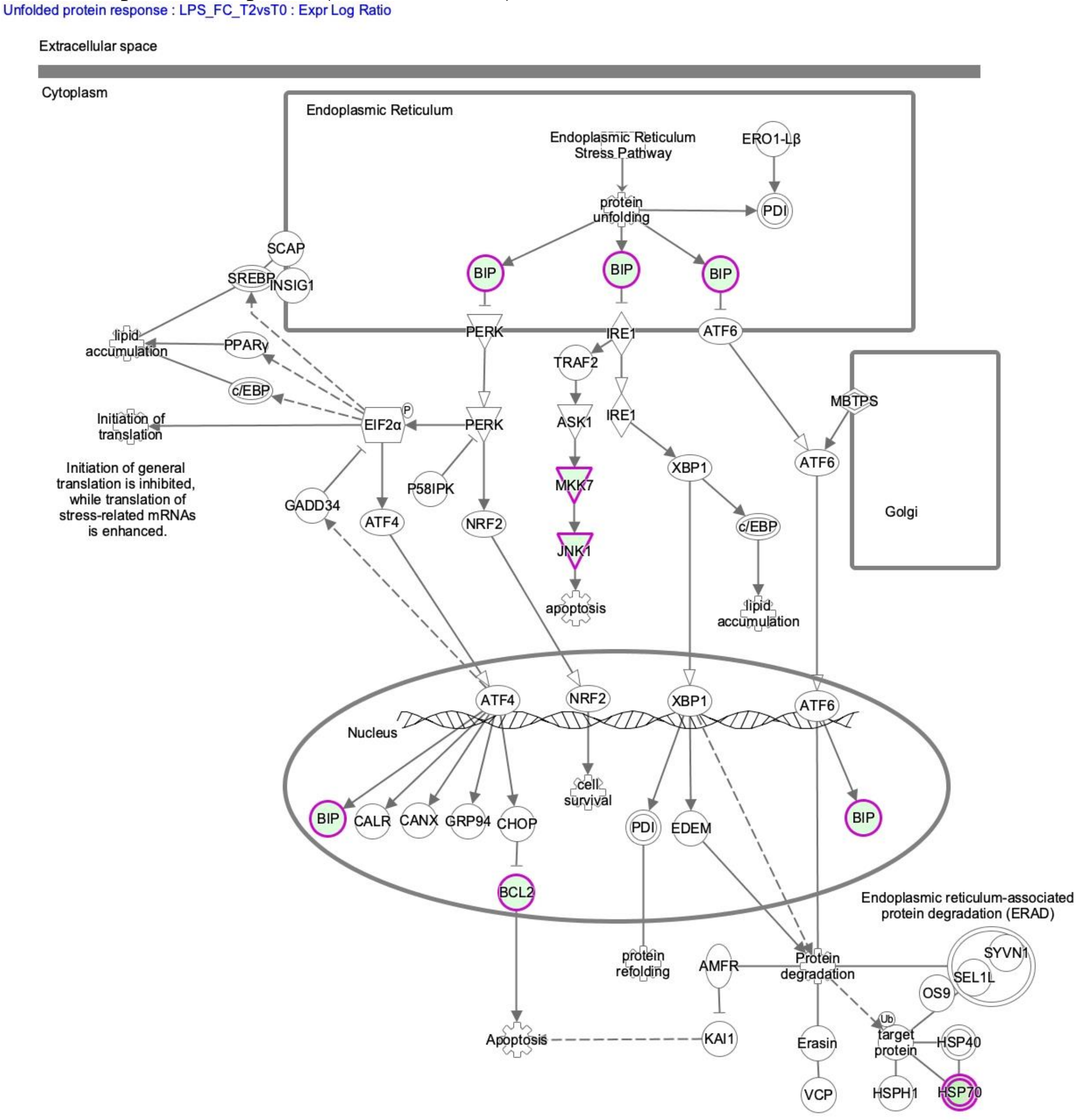

© 2000-2020 QIAGEN. All rights reserved.

Dopamine-DARPP32 Feedback in cAMP Signaling *(z-score= -2.33)*

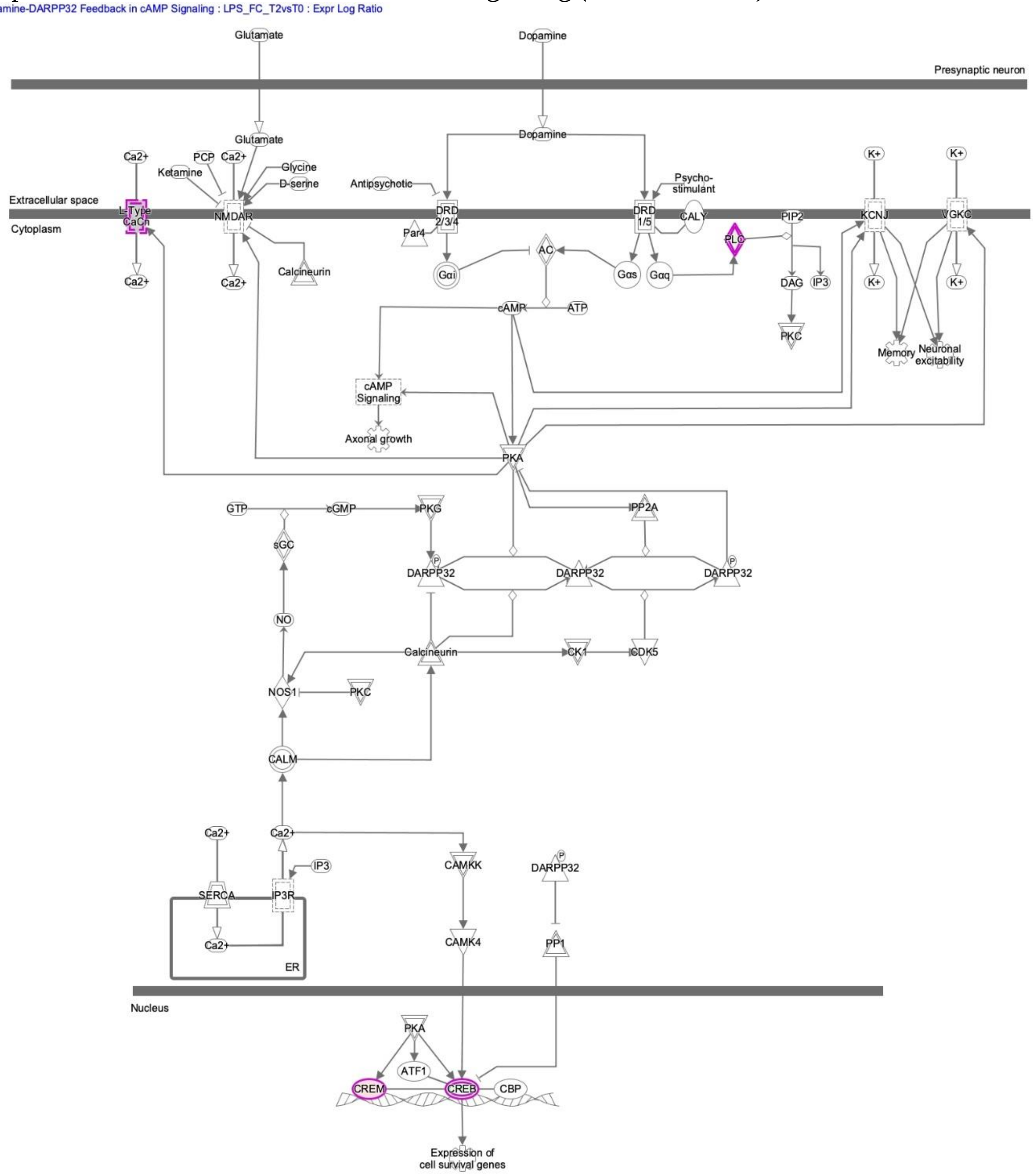

© 2000-2020 QIAGEN. All rights reserved.

Cardiac Hypertrophy Signaling *(z-score= -2.40)*

Cardiac Hypertrophy Signaling : LPS_FC_T2vsT0 : Expr Log Ratio

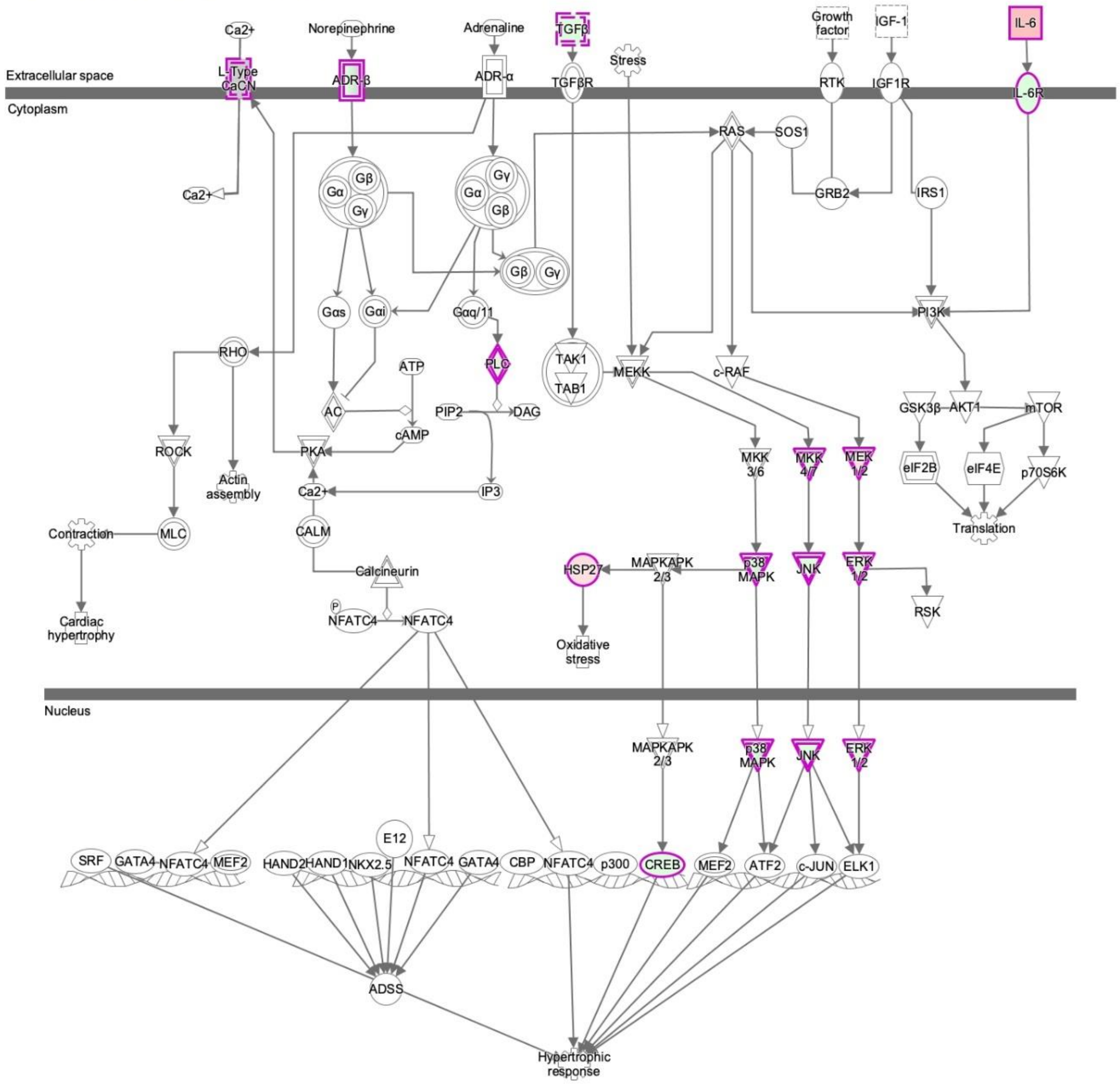

© 2000-2020 QIAGEN. All rights reserved.

Inhibition of Angiogenesis by TSP1 *(z-score= -3.00)*

Inhibition of Angiogenesis by TSP1 : LPS_FC_T2vsT0 : Expr Log Ratio

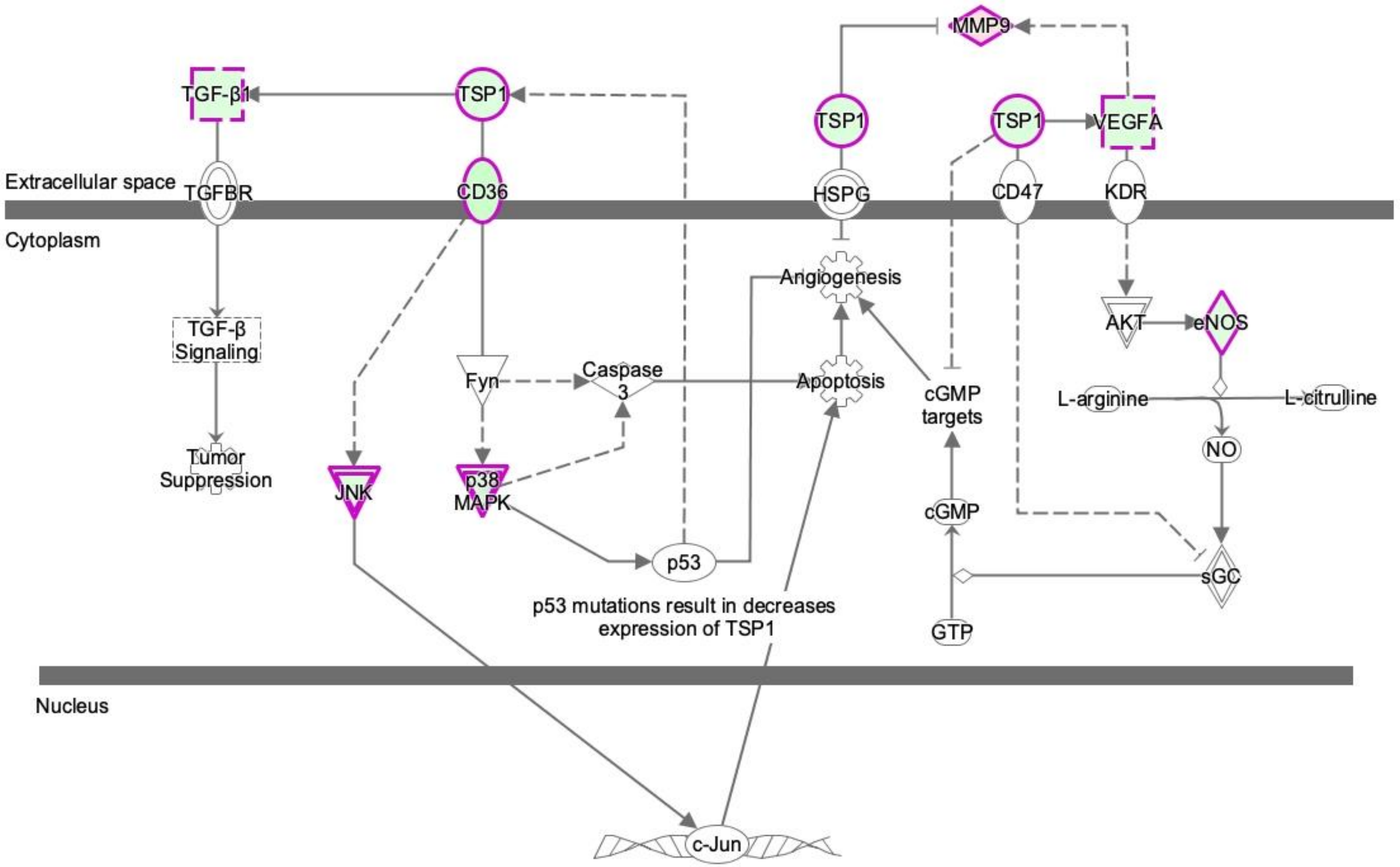

© 2000-2020 QIAGEN. All rights reserved.

Pathways altered in CON group

Crosstalk between Dendritic Cells and Natural Killer Cells *(z-score= 2.65)*

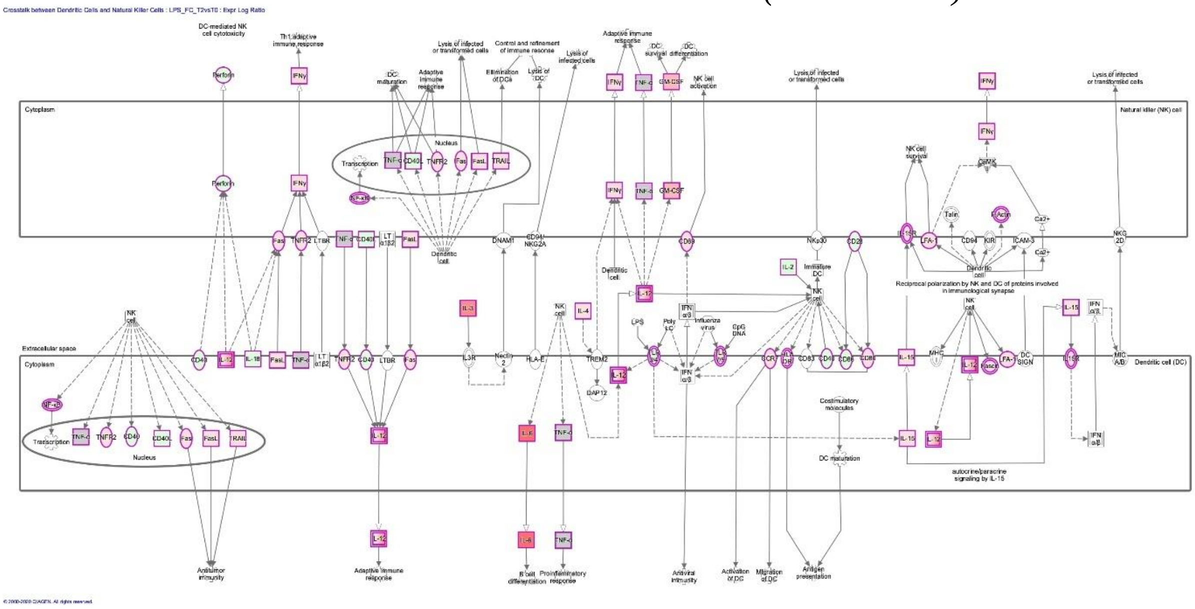

Neuroinflammation Signaling Pathway *(z-score= 2.32)*

Neuroinflammation Signaling Pathway : LPS_FC_T2vsT0 : Expr Log Ratio

Neuroinflammation involves numerous cell types, acts to clear neuronal damage, and plays a key role in maintaining the homeostasis of CNS. Homeostasis can be lost through various regulatory failures, or when humoral immune components cross the blood-brain barrier, causing chronic inflammation with excessive cell and tissue damage, which is associated with neurodegenerative diseases.

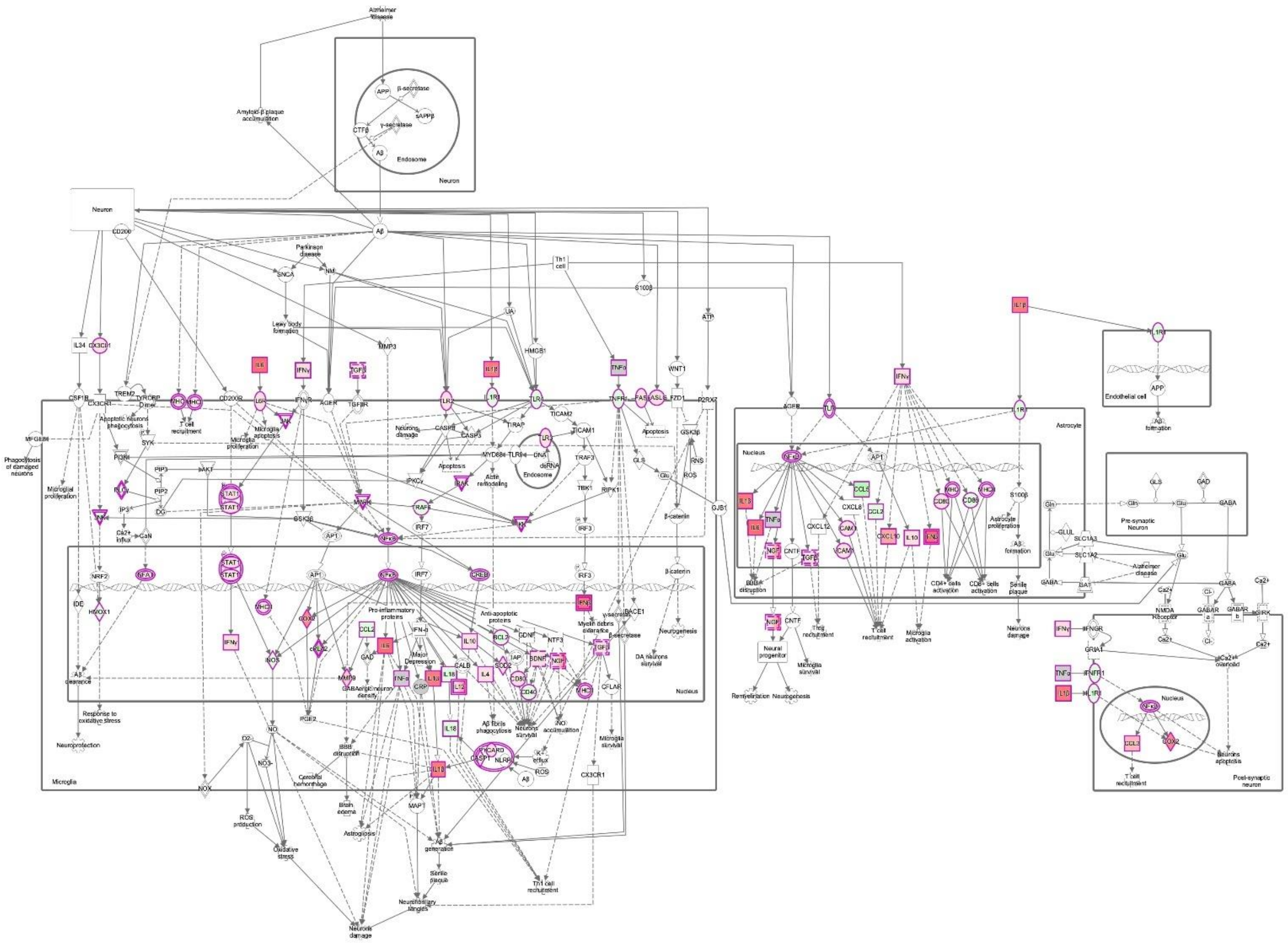

© 2000-2020 QIAGEN. All rights reserved.

Systemic Lupus Erythematosus In B Cell Signaling Pathway *(z-score= 2.31)*

Systemic Lupus Erythematosus In B Cell Signaling Pathway : LPS_FC_T2vsT0 : Expr Log Ratio

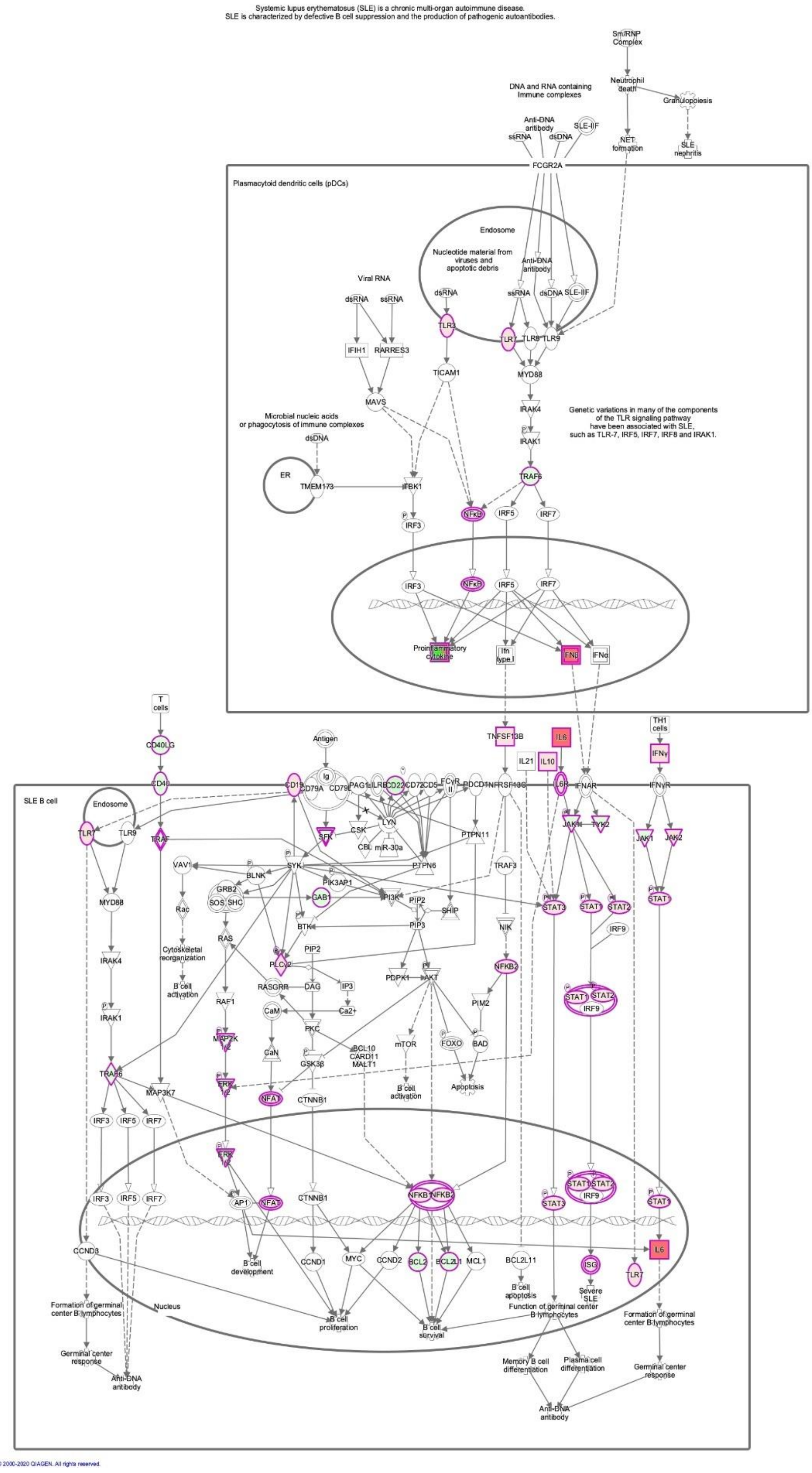

© 2000-2020 QIAGEN. All rights reserved.

Th17 Activation Pathway *(z-score= 2.20)*

Th17 Activation Pathway : LPS_FC_T2vsT0 : Expr Log Ratio

TH17 cells are characterized by the production of IL-17A, IL-17F and IL-22 cytokines which promote the clearance of extracellular bacteria and fungi in the gastrointestinal tract, airway, lungs, and skin.
Th17 cells can be associated with the pathogenesis of several autoimmune and inflammatory diseases.

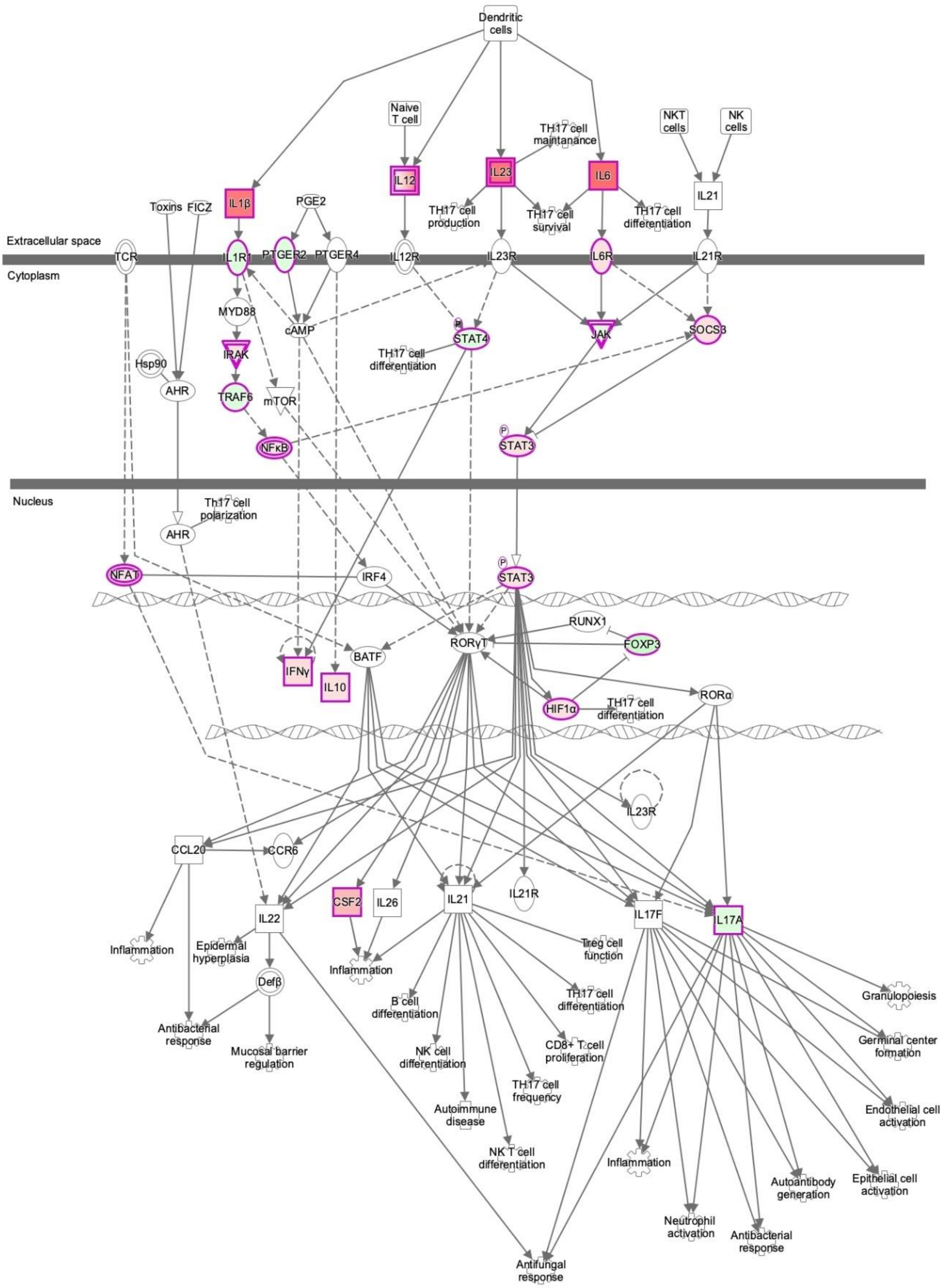

© 2000-2020 QIAGEN. All rights reserved.

T Cell Exhaustion Signaling Pathway *(z-score= 2.12)*

T Cell Exhaustion Signaling Pathway : LPS_FC_T2vsT0 : Expr Log Ratio

T cell exhaustion is a state of dysfunction that commonly occurs during chronic infections and cancer.
T cell exhaustion is characterized by progressive loss of effector functions, reduced proliferative capacity, and failure of memory T cell differentiation.

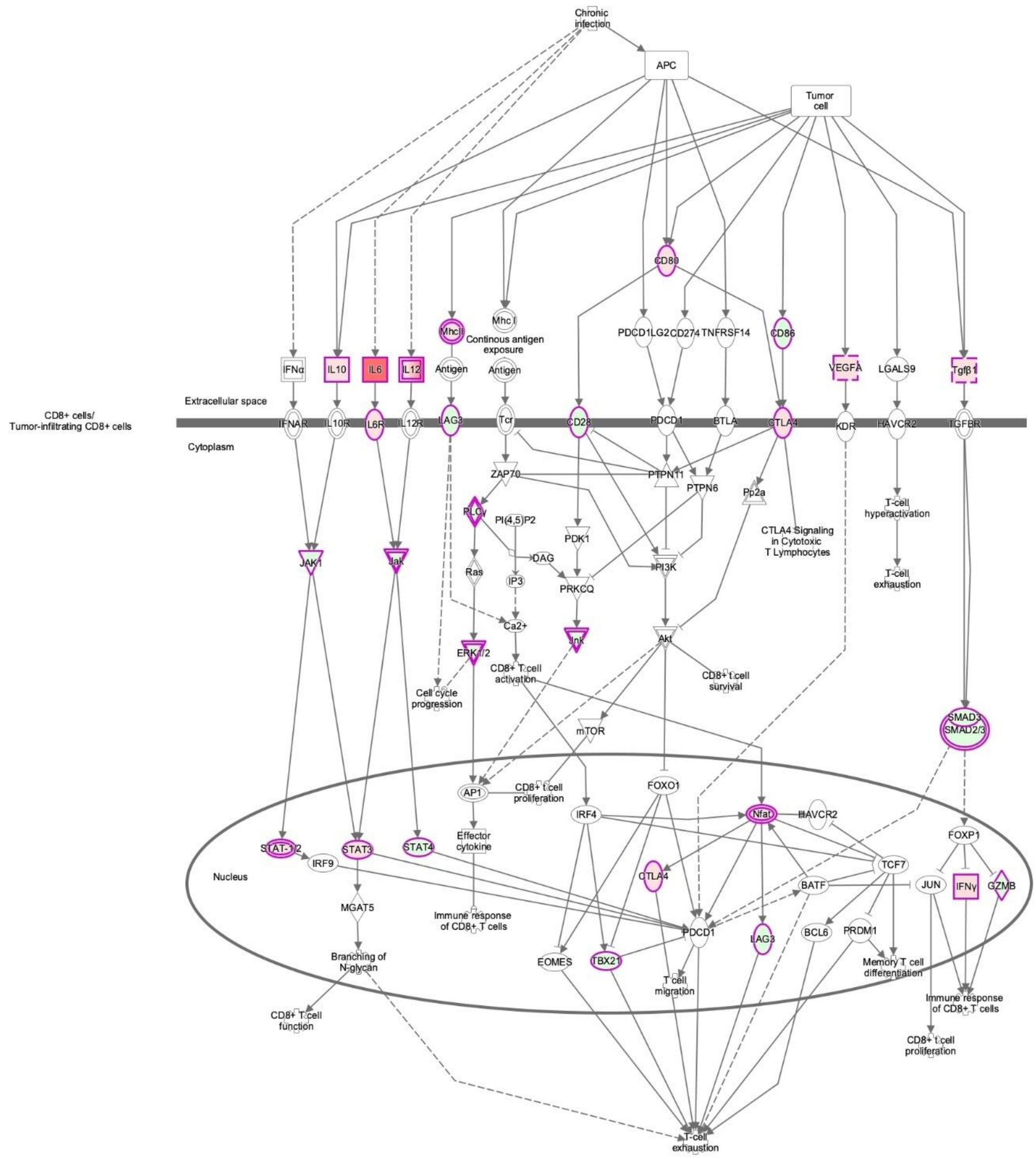

© 2000-2020 QIAGEN. All rights reserved.

TREM1 Signaling *(z-score= 2.04)*

[TREM-1: intracellular signaling pathways and interaction with pattern recognition receptors - PubMed (nih.gov)]

TREM1 Signaling : LPS_FC_T2vsT0 : Expr Log Ratio

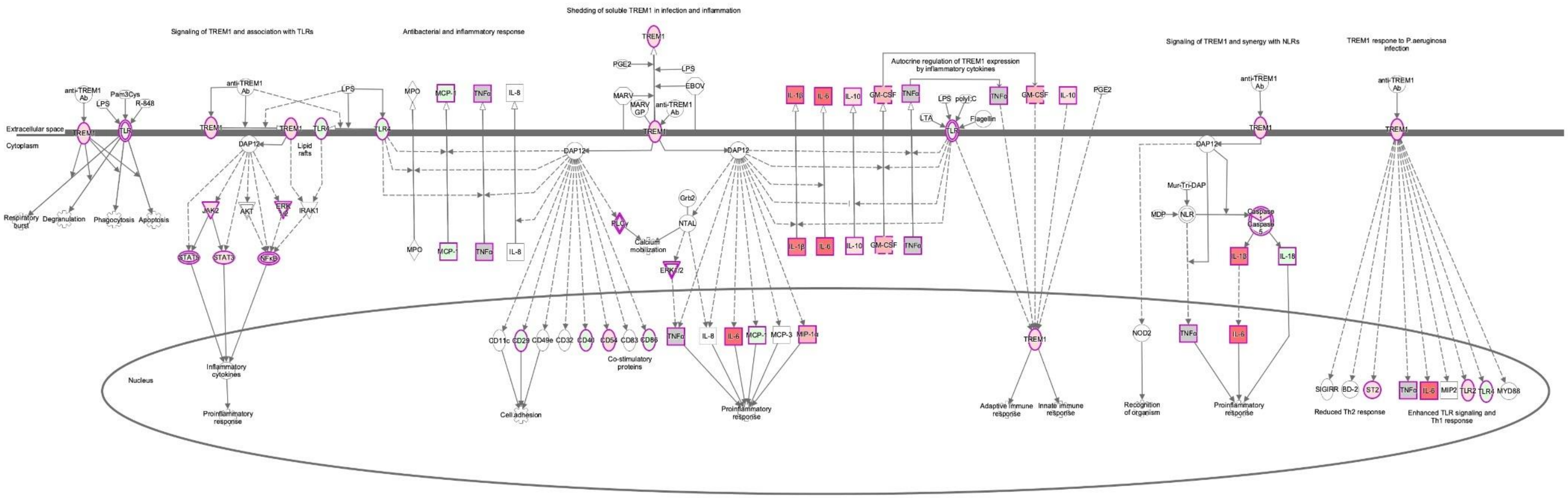

© 2000-2020 QIAGEN. All rights reserved.
