# Supplementary material for: Inflammation-Related Genes Are Differentially Expressed in Lipopolysaccharide-Stimulated Peripheral Blood Mononuclear Cells after 3 Months of Resistance Training in Older Women
Source: Cells. 2024 Aug 25;13(17):1416. doi: 10.3390/cells13171416 (PMC11394400; doi:10.3390/cells13171416)
Supplement: Supplementary file 1 [file cells-13-01416-s001.zip › SALIMANS_LPS-RNAseq_Cells_Supplementary Table S1.pdf]

Supplementary Table S1 Overview of the training and LPS-induced effects on the expression of genes that were significantly altered in at least one of the intervention groups, and for which the exact immune-modulatory role regarding exercise has not yet been described. The columns 'Baseline' and '3 months' show the LPS-induced effects on the gene expression before intervention (Baseline) and after intervention (3 months). '3 months/Baseline': Showing the exercise-induced effects on the gene expression. A. Orange: pro-inflammatory genes, B. Green: anti-inflammatory genes, C. Grey: genes of which the exact immune-modulatory role regarding exercise has not yet been described. Dark red: exercise-induced increase in upregulation following LPS stimulation, Pink: exercise-induced increase in downregulation following LPS stimulation, Light blue: exercise-induced decrease in upregulation following LPS stimulation, Dark blue: exercise-induced decrease in downregulation following LPS stimulation, White: Genes expression was not altered after the three months intervention in LPS stimulated PBMCs (FC=1), Purple: Exercise-induced change in gene expression from upregulation to downregulation following LPS stimulation, Orange: Exercise-induced change in gene expression from downregulation to upregulation following LPS stimulation, Black text: showing a significant clinically relevant foldchange (FC≤0.67 or FC≥1.5). Grey text: showing a tendency towards clinically relevant foldchange (1<FC<1.5 or 0.67<FC<1), which the expression was significantly changed in at least one of the intervention groups. Dark red: exercise-induced increase in upregulation following LPS stimulation, Pink: exercise-induced increase in downregulation following LPS stimulation, Light blue: exercise-induced decrease in upregulation following LPS stimulation, Dark blue: exercise-induced decrease in downregulation following LPS stimulation, White with grey text: Genes expression was not altered after the three months intervention in LPS stimulated PBMCs (FC=1), Purple: Exercise-induced change in gene expression from upregulation to downregulation following LPS stimulation, Orange: Exercise-induced change in gene expression from downregulation to upregulation following LPS stimulation, Black text: showing a significant clinically relevant foldchange (FC≤0.67 or FC≥1.5). White text: showing a tendency towards clinically relevant foldchange (1<FC<1.5 or 0.67<FC<1). IST: Intensive Strength Training, SET: Strength Endurance Training, CON: Flexibility training (Control group).

		IST				SET				CON		
**Genes**	**Name**	**Baseline**	**3 Months**	**3 months/ Baseline**		**Baseline**	**3 Months**	**3 months/ Baseline**		**Baseline**	**3 Months**	**3 months/ Baseline**
A.												
AREG	*Amphiregulin*	1,23	4,21	3,41*		1,88	1,67	0,89		1,70	2,04	1,20
ASPN	*Asporin*	0,41	0,86	2,11*		1,00	0,76	0,76		0,67	0,77	1,15
BDKRB1	*Bradykinin receptor B1*	0,49	1,79	3,67*		0,73	0,93	1,27		0,05	0,31	6,79*
BDKRB2	*Bradykinin receptor B2*	0,63	0,30	0,48*		0,37	0,38	1,03		0,45	0,33	0,74
BMX	*BMX non-receptor tyrosine kinase*	0,85	0,62	0,72		0,49	0,30	0,61*		0,52	0,50	0,96
CCL19	*C-C motif chemokine ligand 19*	4,44	2,33	0,52*		9,61	11,19	1,16		5,82	22,66	3,89*
CCL3	*C-C motif chemokine ligand 3*	3,44	3,83	1,11		9,52	11,85	1,25		7,06	15,51	2,20*
CCL5	*C-C motif chemokine ligand 5*	1,02	1,10	1,08		0,94	0,89	0,94		1,21	0,62	0,51*
CD14	*Cluster of Differentiation 14*	1,92	1,24	0,65*		1,25	1,62	1,29		1,69	1,96	1,16
CD22	*Cluster of Differentiation 22*	0,89	1,04	1,17		1,11	1,18	1,07		1,38	0,90	0,65*
CD36	*Cluster of differentiation 36*	0,04	0,10	2,25*		0,10	0,05	0,57*		0,04	0,03	0,77
CD38	*Cluster of Differentiation 38*	0,96	1,85	1,93*		1,29	1,89	1,47		1,19	1,60	1,35
CD68	*Cluster of Differentiation 68*	0,53	0,81	1,54*		0,67	0,74	1,11		0,88	0,74	0,84
CLEC5A	*C-type lectin domain containing 5A*	1,00	2,91	2,91*		0,65	1,00	1,55*		1,10	0,41	0,37*

		IST			SET			CON		
Genes	Name	Baseline	3 Months	3 months/ Baseline	Baseline	3 Months	3 months/ Baseline	Baseline	3 Months	3 months/ Baseline
COL3A1	*Collagen type III alpha 1 chain*	0,20	0,68	3,37*	1,11	1,22	1,10	0,66	0,61	0,93
COL4A1	*Collagen type IV alpha 1 chain*	0,30	0,55	1,81*	0,34	0,87	2,60*	0,76	0,90	1,17
COL4A5	*Collagen type IV alpha 5 chain*	1,32	0,90	0,68	3,24	0,91	0,28*	0,99	1,06	1,07
CPA2	*Carboxypeptidase A2*	0,18	0,46	2,47*	1,05	0,28	0,27*	0,27	0,46	1,70*
CRP	*C-reactive protein*	1,00	1,81	1,81*	3,35	1,00	0,30*	0,74	1,00	1,36
CSF2	*Colony Stimulating Factor 2*	4,27	21,17	4,95*	30,26	32,78	1,08	9,70	21,10	2,17*
CSF3	*Colony Stimulating Factor 3*	9,32	4,52	0,48*	45,47	26,92	7,19*	36,29	684,67	18,86*
CX3CL1	*C-X3-C motif chemokine ligand 1*	0,80	0,87	1,08	1,43	0,81	0,56*	0,48	0,50	1,05
CXCL10	*C-X-C motif chemokine ligand 10*	0,01	0,07	8,94*	0,02	0,05	2,87*	0,00	0,01	2,66*
CXCL11	*C-X-C motif chemokine ligand 11*	0,01	0,27	23,82*	0,04	0,11	3,08*	0,01	0,05	7,26*
CXCR1	*C-X-C Motif Chemokine Receptor 1*	0,40	0,46	1,14	0,30	0,03	0,11*	0,26	0,29	1,11
CYR61	*Cysteine rich angiogenic inducer 61*	0,49	1,16	2,36*	1,08	1,52	1,41	1,05	1,18	1,13
DEFA3	*Defensin alpha 3*	0,54	0,50	0,93	0,36	0,48	1,34	0,45	0,67	1,51*
DNM1P46	*Dynamin 1 pseudogene 46*	1,35	2,57	1,90*	3,17	1,33	0,42*	0,74	0,32	0,44*
EDN1	*Endothelin 1*	1,14	1,97	1,72*	2,22	3,41	1,53*	2,35	2,10	0,89
EDNRB	*Endothelin receptor type B*	0,14	0,10	0,67*	0,14	0,03	0,23*	0,07	0,08	1,07
EPHB2	*EPH receptor B2*	0,08	0,03	0,40*	0,03	0,03	0,95	0,04	0,03	0,76
EREG	*Epiregulin*	4,53	3,22	0,71	4,65	4,55	0,98	7,06	12,06	1,71*
FCAR	*Fc fragment of IgA receptor*	2,04	2,19	1,07	1,20	3,53	2,96*	2,67	2,49	0,93
HIF1A	*Hypoxia inducible factor 1 subunit alpha*	3,00	1,96	0,65*	2,65	2,66	1,00	3,63	3,70	1,02
HIF3A	*Hypoxia inducible factor 3 alpha subunit*	0,95	2,49	2,62*	0,40	0,06	0,15*	1,08	0,21	0,19*
HLA-DRA	*Major histocompatibility complex, class II, DR alpha*	0,37	0,58	1,56*	0,31	0,33	1,08	0,08	0,11	1,29
HLA-DRB5	*Major histocompatibility complex, class II, DR beta 5*	0,22	0,26	1,16	0,63	0,35	0,56*	0,11	0,12	1,08
HTR3B	*5-Hydroxytryptamine Receptor 3B*	0,34	0,39	1,15	1,08	0,65	0,60*	0,66	0,16	0,24*
IFNG	*Interferon gamma*	1,43	1,94	1,36	1,33	2,68	2,02*	0,58	0,85	1,46
IL12A	*Interleukin 12A*	0,83	1,53	1,84*	1,39	1,22	0,88	0,97	1,04	1,08
IL12B	*Interleukin 12 beta*	4,46	12,40	2,78*	9,19	14,22	1,55*	5,77	26,68	4,63*
IL17A	*Interleukin 17A*	0,99	0,38	0,38*	0,38	0,72	1,89*	0,78	0,71	0,91

Genes	Name	IST			SET			CON		
		Baseline	3 Months	3 months/ Baseline	Baseline	3 Months	3 months/ Baseline	Baseline	3 Months	3 months/ Baseline
IL18	*Interleukin 18*	0,20	0,32	1,59*	0,21	0,16	0,73	0,25	0,25	0,96
IL1A	*Interleukin 1 alpha*	4,62	4,09	0,88	12,01	14,46	1,20	9,17	51,71	5,64*
IL1B	*Interleukin 1 beta*	6,08	3,56	0,59*	12,83	11,90	0,93	10,55	46,61	4,42*
IL1RL1	*Cluster of differentiation 160*	0,94	1,86	1,98*	1,53	1,23	0,81	1,31	1,32	1,01
IL2RA	*Interleukin 2 receptor subunit alpha*	4,14	2,28	0,55*	7,18	8,52	1,19	7,78	7,75	1,00
IL3	*Interleukin 3*	0,57	0,32	0,56*	0,17	0,35	2,14*	0,06	0,22	3,57*
IL6	*Interleukin 6*	8,87	5,92	0,67*	25,48	45,87	1,80*	19,80	99,99	5,05*
ITGAM	*Integrin subunit alpha M*	0,56	0,53	0,94	0,35	0,38	1,08	0,39	0,61	1,57*
JAK2	*Janus kinase 2*	0,47	0,76	1,61*	0,72	0,65	0,90	0,46	0,50	1,11
KLK15	*Kallikrein related peptidase 15*	5,28	0,53	0,10*	0,34	1,09	3,20*	0,15	1,50	9,87*
KLK2	*Kallikrein Related Peptidase 2*	0,75	1,14	1,52*	1,38	2,23	1,61*	1,03	0,82	0,80
KLK3	*Kallikrein Related Peptidase 3*	1,00	1,10	1,10	1,00	1,00	1,00	0,22	1,00	4,61*
KNG1	*Kininogen 1*	0,79	0,61	0,77	1,24	0,89	0,72	1,02	0,66	0,64*
LEP	*Leptin*	0,21	0,10	0,45*	0,18	0,01	0,08*	0,41	0,06	0,15*
LRP2	*LDL receptor related protein 2*	0,48	3,79	7,83*	1,38	0,77	0,56*	1,97	1,42	0,72
LTB4R2	*Leukotriene B4 receptor*	1,78	1,00	0,56*	3,10	1,00	0,32*	1,00	1,00	1,00
LTB4R2_A	*Leukotriene B4 receptor 2a*	0,58	2,25	3,90*	0,41	2,37	5,72*	0,26	0,54	2,11*
LTC4S	*Leukotriene C4 Synthase*	1,00	0,67	0,67*	2,14	1,00	0,47*	0,74	1,00	1,36
MMP9	*Matrix metallopeptidase 9*	1,27	0,94	0,74	0,43	0,52	1,20	0,67	1,22	1,81*
NCAM1	*Neural cell adhesion molecule 1*	2,56	2,18	0,85	0,56	0,35	0,62*	1,38	2,54	1,85*
NCR1	*Natural cytotoxicity triggering receptor 1*	0,30	1,00	3,38*	1,94	1,00	0,52*	3,03	1,00	0,33*
NOS2	*Nitric oxide synthase 2*	0,76	0,71	0,94	0,60	1,42	2,34*	0,69	0,66	0,97
PDE4C	*Phosphodiesterase 4C*	1,41	1,01	0,71	1,37	1,28	0,93	1,50	3,13	2,09*
PGF	*Placental growth factor*	0,39	0,73	1,89*	0,42	0,56	1,33	0,46	0,56	1,23
PGK1	*Phosphoglycerate kinase 1*	0,40	1,09	2,71*	12,41	1,99	0,16*	1,70	0,35	0,21*
PLA2G1B	*Phospholipase A2 group IB*	2,19	0,65	0,30*	0,24	0,18	0,75	1,01	1,70	1,69*
PLA2G2A	*Phospholipase A2 group V*	1,89	4,33	2,29*	0,26	1,76	6,71*	1,00	1,00	1,00
PLA2G2D	*Phospholipase A2 Group IID*	0,40	0,43	1,07	0,57	0,21	0,37*	0,20	0,69	3,43*
PLA2G5	*Phospholipase A2 group V*	0,56	1,39	2,50*	0,60	0,84	1,40	3,44	1,22	0,36*

		IST			SET			CON		
**Genes**	**Name**	**Baseline**	**3 Months**	**3 months/ Baseline**	**Baseline**	**3 Months**	**3 months/ Baseline**	**Baseline**	**3 Months**	**3 months/ Baseline**
PRKAA2	*Protein kinase AMP-activated catalytic subunit alpha 2*	0,09	1,89	21,43*	1,14	1,59	1,40	0,93	0,55	0,59*
PTGER3	*Prostaglandin E receptor 3*	0,32	0,48	1,48	0,36	0,21	0,58*	0,25	0,76	3,01*
PTGS2	*Prostaglandin-endoperoxide synthase 2*	11,82	4,00	0,34*	76,96	122,88	1,60*	64,53	244,02	3,78*
S100A8	*S100 calcium binding protein A8*	2,28	1,28	0,56*	1,43	1,93	1,34	1,52	2,27	1,49
S100A9	*S100 calcium binding protein A9*	2,68	1,61	0,60*	2,27	2,92	1,29	2,11	3,00	1,42
SELE	*Selectin 1*	0,63	0,74	1,16	0,92	0,59	0,65*	0,57	0,91	1,59*
SLC2A4	*Solute carrier family 2 member 4*	2,14	1,34	0,63*	2,66	2,14	0,80	1,53	1,05	0,69
STAT1	*Signal transducer and activator of transcription 1*	0,49	0,84	1,70*	0,68	0,82	1,21	0,37	0,41	1,11
STAT2	*Signal transducer and activator of transcription 2*	0,69	1,07	1,55*	0,74	0,85	1,14	0,65	0,70	1,08
TERC	*Telomerase RNA component*	0,51	0,68	1,32	0,72	0,35	0,49*	0,79	0,42	0,53*
THY1	*Thy-1 cell surface antigen*	43,71	1,69	0,04*	0,14	0,87	6,34*	0,12	0,48	4,10*
TLR4	*Toll like receptor 4*	0,33	0,53	1,58*	0,38	0,39	1,01	0,35	0,33	0,95
TLR7	*Toll like receptor 7*	0,12	0,33	2,79*	0,30	0,20	0,69	0,10	0,12	1,21
TNFRSF1B	*TNF receptor superfamily member 1B*	3,83	2,41	0,63*	3,80	3,72	0,98	4,42	4,54	1,03
TNFSF10	*Tumor Necrosis Factor (Ligand) Superfamily, Member 10*	0,43	1,04	2,42*	0,74	0,86	1,16	0,52	0,75	1,44
TNFSF13B	*TNF superfamily member 13b*	0,42	0,79	1,90*	0,51	0,57	1,12	0,37	0,46	1,24
TREM1	*Colony Stimulating Factor 3 Receptor*	3,87	2,25	0,58*	3,87	3,55	0,92	4,73	7,05	1,49
VEGFA	*Vascular endothelial growth factor A*	4,60	2,08	0,45*	3,67	3,57	0,97	7,10	7,96	1,12
VEGFC	*Vascular endothelial growth factor C*	0,70	0,82	1,17	0,40	0,87	2,19*	0,93	1,08	1,16
B										
ABCA1	*ATP binding cassette subfamily A member 1*	2,25	1,30	0,58*	1,50	1,68	1,12	2,99	3,56	1,19
ADIPOQ	*Adiponectin, C1Q and collagen domain containing*	2,52	1,00	0,40*	1,00	1,00	1,00	1,00	1,00	1,00
AGTR1	*Angiotensin II receptor type 1*	0,79	0,51	0,64*	1,32	0,49	0,37*	0,60	2,19	3,63*
ARNT2	*Aryl hydrocarbon receptor nuclear translocator 2*	4,64	1,77	0,38*	3,47	10,03	2,89*	6,84	5,09	0,75
BDNF	*Brain derived neurotrophic factor*	0,62	1,19	1,92*	0,95	0,44	0,46*	0,91	1,12	1,23
C1QTNF9	*C1q and tumor necrosis factor related protein 9*	0,63	0,90	1,43	3,44	1,21	0,35*	0,97	0,46	0,47*
CA4	*Carbonic anhydrase 4*	0,36	0,56	1,57*	0,18	0,05	0,29*	0,17	0,12	0,71
CSF1	*Colony Stimulating Factor 1*	0,85	1,32	1,55*	0,60	0,31	0,51*	0,33	1,27	3,82*
FCGR3B	*Fc Fragment Of IgG Receptor IIIb*	0,26	0,31	1,18	0,23	0,03	0,11*	0,12	0,12	0,97

		IST			SET			CON		
**Genes**	**Name**	**Baseline**	**3 Months**	**3 months/ Baseline**	**Baseline**	**3 Months**	**3 months/ Baseline**	**Baseline**	**3 Months**	**3 months/ Baseline**
FGF21	*Fibroblast growth factor 21*	0,65	1,00	1,53*	3,35	0,89	0,27*	1,00	0,17	0,17*
FGF6	*Fibroblast growth factor 6*	2,01	1,19	0,60*	3,90	0,69	0,18*	3,15	5,83	1,85*
FN1	*Fibronectin 1*	0,01	0,02	2,13*	0,01	0,01	0,75	0,00	0,00	1,63*
FOXP3	*Forkhead box P3*	0,52	1,40	2,70*	0,94	0,80	0,86	1,20	0,73	0,61*
FRZB	*Frizzled-related protein*	1,34	0,90	0,67*	0,33	0,66	1,96*	1,67	3,21	1,92*
HRH2	*Histamine receptor H2*	2,63	1,25	0,48*	1,72	2,19	1,27	1,82	2,35	1,29
HRH3	*Histamine receptor H3*	1,58	0,78	0,50*	1,90	0,61	0,32*	1,07	0,92	0,86
HSPA12A	*Heat shock protein family A (Hsp70) member 12 A*	1,00	1,00	1,00	1,00	1,00	1,00	0,32	1,00	3,09*
HSPA1A	*Heat shock 70kDa protein 1A*	1,41	0,89	1,00	0,74	0,94	1,27	1,34	1,48	1,10
HSPA1B	*Heat shock 70kDa protein 1B*	1,85	1,19	0,64*	0,93	0,90	0,97	0,79	0,83	1,05
HSPA1L	*Heat shock 70 kDa protein 1L*	1,78	4,21	2,37*	2,14	0,86	0,40*	1,64	1,00	0,61*
IGF2	*Insulin growth factor*	1,00	1,00	1,00	1,00	0,30	0,30*	0,74	1,00	1,36
IL10	*Interleukin 10*	4,73	2,59	0,55*	6,82	6,11	0,89	7,91	8,96	1,13
IL13	*Interleukin 13*	2,51	2,56	1,02	2,41	3,97	1,65*	2,26	2,79	1,23
IL1R2	*Interleukin 1 receptor type 2*	0,34	0,19	0,55*	0,09	0,21	2,37*	0,19	0,10	0,51*
IL1RAPL2	*Interleukin 1 receptor accessory protein like 2*	0,65	1,00	1,53*	1,00	1,00	1,00	1,64	1,00	0,61*
IL2	*Interleukin 2*	0,56	1,09	1,93*	0,57	0,52	0,92	0,57	0,42	0,72
IL4	*Interleukin 4*	0,89	1,39	1,57*	1,57	1,48	0,95	1,05	1,28	1,22
IL9	*Interleukin 9*	10,05	1,57	0,16*	6,13	8,79	1,44	1,58	1,56	0,99
LYZ	*Lysozyme*	0,25	0,29	1,18	0,11	0,09	0,80	0,08	0,15	1,75*
MC2R	*Melanocortin 2 receptor*	1,00	1,00		1,00	2,97	2,97*	1,64	0,41	0,25*
MXRA5	*Matrix remodeling associated 5*	1,38	6,09	4,42*	0,24	0,14	0,59*	3,84	0,48	0,12*
ORM1	*Orosomucoid 1*	0,99	0,65	0,66*	0,77	11,88	15,37*	1,34	1,06	0,79
PTGIS	*Prostaglandin I2 synthase*	3,00	0,36	0,12*	10,53	0,61	0,06*	0,38	0,81	2,15*
RFC2	*Replication factor C subunit 2*	1,00	0,32	0,32*	1,00	0,56	0,56*	1,00	1,00	1,00
RNASE2	*Ribonuclease A family member 2*	1,02	0,75	0,73	1,06	0,63	0,60*	1,16	1,10	0,95
SOCS3	*Suppressor of cytokine signaling 3*	4,45	2,48	0,56*	4,61	6,78	1,47	6,97	9,89	1,42
THBD	*Thrombomodulin precursor*	2,63	4,56	1,73*	4,14	3,46	0,84	5,05	6,10	1,21
TNIP3	*TNFAIP3 interacting protein 3*	7,17	2,52	0,35*	10,76	17,33	1,61*	13,12	26,08	1,99*

Genes	Name	IST			SET			CON		
		Baseline	3 Months	3 months/ Baseline	Baseline	3 Months	3 months/ Baseline	Baseline	3 Months	3 months/ Baseline
C										
ADRB1	*Adrenoceptor beta 1*	0,71	0,62	0,87	0,56	0,42	0,76	0,47	0,72	1,54*
CACNA2D1	*Calcium voltage-gated channel auxiliary subunit alpha2delta 1*	1,96	3,40	1,74*	0,93	3,17	3,41*	2,65	2,48	0,94
CDA	*Cytidine deaminase*	1,28	0,81	0,63*	0,78	0,83	1,07	1,03	1,31	1,28
CDH1	*Cadherin 1*	1,87	2,47	1,32	2,50	2,71	1,09	2,94	4,49	1,53*
CES1	*Carboxylesterase 1*	1,41	1,12	0,80	1,07	1,27	1,19	1,52	2,35	1,55*
COL4A2	*Collagen type IV alpha 2 chain*	0,85	1,00	1,18	0,55	0,37	0,66*	0,73	0,41	0,57*
CYP1A2	*Cytochrome P450 family 1 subfamily A member 2*	1,87	1,34	0,71	1,66	3,01	1,81*	2,58	1,77	0,68
CYP7A1	*Cytochrome P450 family 7 subfamily A member 1*	0,99	2,03	2,04*	0,46	1,74	3,82*	0,74	0,96	1,29
GJB6	*Gap junction protein beta 6*	1,93	1,22	0,63*	1,73	2,73	1,58*	2,67	2,97	1,12
HPGD	*15-hydroxyprostaglandin dehydrogenase*	0,63	1,11	1,76*	1,05	0,80	0,76	1,04	1,01	0,97
MARC1	*Mitochondrial amidoxime reducing component*	0,53	0,48	0,91	0,66	0,37	0,57*	0,43	0,58	1,36
MYH6	*Myosin heavy chain 6*	1,41	1,08	0,77	1,37	0,98	0,71	5,05	0,80	0,16*
OLIG2	*Oligodendrocyte lineage transcription factor 2*	0,82	1,29	1,58*	0,54	1,17	2,17*	0,63	1,67	2,65*
PARD6G	*Par-6 family cell polarity regulator gamma*	2,03	1,33	0,65*	1,18	1,34	1,14	1,53	1,58	1,03
PDGFA	*Platelet derived growth factor subunit A*	4,83	3,31	0,69	4,80	7,31	1,52*	5,39	8,73	1,62*
PDGFC	*Platelet derived growth factor C*	0,22	0,41	1,88*	0,34	0,30	0,88	0,42	0,33	0,77
PPARGC1A	*Peroxisome proliferator-activated receptor gamma coactivator 1-alpha*	0,31	0,22	0,71	0,12	0,07	0,57*	0,28	0,23	0,83
PRM1	*Protamine 1*	1,38	0,91	0,66*	0,86	1,75	2,03*	2,18	1,57	0,72
PTGDS	*Prostaglandin D2 synthase*	0,35	0,56	1,59*	0,64	0,37	0,57*	0,48	0,43	0,91
PTGFR	*Prostaglandin F receptor*	2,64	0,60	0,23*	0,98	7,04	7,22*	3,55	3,91	1,10
RPL3L	*Ribosomal protein L3 like*	0,84	1,43	1,70*	0,54	0,38	0,71	0,65	0,73	1,13
RTEL1	*Regulator of telomere elongation helicase 1*	1,03	0,99	0,96	0,89	0,92	1,03	1,41	0,71	0,50*
TFRC	*Transferrin receptor*	1,93	1,29	0,67*	1,37	1,51	1,10	2,36	2,33	0,99
