# Supplementary material for: Inflammation-Related Genes Are Differentially Expressed in Lipopolysaccharide-Stimulated Peripheral Blood Mononuclear Cells after 3 Months of Resistance Training in Older Women
Source: Cells. 2024 Aug 25;13(17):1416. doi: 10.3390/cells13171416 (PMC11394400; doi:10.3390/cells13171416)
Supplement: Supplementary file 1 [file cells-13-01416-s001.zip › SALIMANS_LPS-RNAseq_Cells_Supplementary Table S2.pdf]

Supplementary Table S2 Overview of the genes which showing a significant fold-change regarding the exercise-induced effects. (FC≤0.67 or FC≥1.5). Dark red (Effects after three months exercise intervention > Effects baseline UP): The number of genes showing an exercise-induced increase in upregulation following LPS stimulation, Pink (Effects after three months exercise intervention > Effects baseline DOWN): The number of genes showing an exercise-induced increase in downregulation following LPS stimulation, Light blue (Effects after three months exercise intervention < Effects baseline UP): The number of genes showing an exercise-induced decrease in upregulation following LPS stimulation, Dark blue (Effects after three months exercise intervention < Effects baseline DOWN): The number of genes showing an exercise-induced decrease in downregulation following LPS stimulation, Purple (UP to DOWN): The number of genes showing a exercise-induced change in gene expression from upregulation to downregulation following LPS stimulation, Orange (DOWN to UP): The number of genes showing an exercise-induced change in gene expression from downregulation to upregulation following LPS stimulation.
Orange genes: pro-inflammatory, Green genes: anti-inflammatory, Grey genes: genes of which the exact immune-modulatory role regarding exercise has not yet been described. Genes presented in light green showing the same effect in the three groups. Genes presented in light purple showing the same effect between IST and SET. Genes presented in grey showing the same effect between IST and FT. Genes presented in Blue showing the same effect in SET and FT. The full gene abbreviations are presented in table 1. IST: Intensive Strength Training, SET: Strength Endurance Training, FT: Flexibility training (Control group).

	IST		SET		FT
Genes	3 months/ baseline	Genes	3 months/ baseline	Genes	3 months/ baseline
IL12B	2,78*	IL12B	1,55*	IL12B	4,63*
LEP	0,45*	LEP	0,08*	LEP	0,15*
CXCL11	23,82*	CXCL11	3,08*	CXCL11	7,26*
CXCL10	8,94*	CXCL10	2,87*	CXCL10	2,66*
CSF2	4,95*			CSF2	2,17*
CPA2	2,47*			CPA2	1,70*
EDN1	1,72*	EDN1	1,53*		
LTB4R2_A	3,90*	LTB4R2_A	5,72*		
COL4A1	1,81*	COL4A1	2,60*		
EDNRB	0,67*	EDNRB	0,23*		
LTB4R2	0,56*	LTB4R2	0,32*		

	IST		SET		FT
Genes	3 months/ baseline	Genes	3 months/ baseline	Genes	3 months/ baseline
CSF1	1,55*			CSF1	3,82*
FN1	2,13*			FN1	1,63*
IL1R2	0,55*			IL1R2	0,51*
RFC2	0,32*	RFC2	0,56*		
HRH3	0,50*	PTGIS	0,06*		
PTGIS	0,12*	HRH3	0,32*		

	IST		SET		FT
Genes	3 months/ baseline	Genes	3 months/ baseline	Genes	3 months/ baseline
OLIG2	1,58*	OLIG2	2,17*	OLIG2	2,65*
CYP7A1	2,04*	CYP7A1	3,82*		
		PDGFA	1,52*	PDGFA	1,62*
		COL4A2	0,66*	COL4A2	0,57*

	IST		SET		FT
Genes	3 months/ baseline	Genes	3 months/ baseline	Genes	3 months/ baseline
AREG	3,41*	FCAR	2,96*	IL1A	5,64*
PLA2G2A	2,29*	IFNG	2,02*	IL1B	4,42*
DNM1P46	1,90*	KLK2	1,61*	CCL19	3,89*

	IST		SET		FT
Genes	3 months/ baseline	Genes	3 months/ baseline	Genes	3 months/ baseline
MXRA5	4,42*	ARNT2	2,89*	FRZB	1,92*
HSPA1L	2,37*	IL13	1,65*	FGF6	1,85*
THBD	1,73*	ORM1	15,37*	AGTR1	3,63*

	IST		SET		FT
Genes	3 months/ baseline	Genes	3 months/ baseline	Genes	3 months/ baseline
CACNA2D1	1,74*	CYP1A2	1,81*	CES1	1,55*
HPGD	1,76*	GJB6	1,58*	CDH1	1,53*
RPL3L	1,70*	PTGFR	7,22*	ADRB1	1,54*

PRKAA2	21,43*
LRP2	7,83*
BDKRB1	3,67*
CLEC5A	2,91*
PGK1	2,71*
HIF3A	2,62*
PLA2G5	2,50*
TNFSF10	2,42*
CYR61	2,36*
IL1RL1	1,98*
CD38	1,93*
IL12A	1,84*
CRP	1,81*
STAT2	1,55*
KLK2	1,52*
NCR1	3,38*
COL3A1	3,37*
TLR7	2,79*
CD36	2,25*
ASPN	2,11*
TNFSF13B	1,90*
PGF	1,89*
STAT1	1,70*
JAK2	1,61*
IL18	1,59*
TLR4	1,58*
HLA-DRA	1,56*
CD68	1,54*
IL6	0,67*
CD14	0,65*
HIF1A	0,65*
TNFRSF1B	0,63*
SLC2A4	0,63*
S100A9	0,60*
IL1B	0,59*
TREM1	0,58*
S100A8	0,56*
IL2RA	0,55*
CCL19	0,52*
CSF3	0,48*
VEGFA	0,45*
PTGS2	0,34*

PLA2G2A	6,71*
NOS2	2,34*
VEGFC	2,19*
IL17A	1,89*
CLEC5A	1,55*
DNM1P46	0,42*
PGK1	0,16*
SELE	0,65*
NCAM1	0,62*
BMX	0,61*
PTGER3	0,58*
CD36	0,57*
HLA-DRB5	0,56*
PLA2G2D	0,37*
HIF3A	0,15*
CXCR1	0,11*
HTR3B	0,60*
LRP2	0,56*
CX3CL1	0,56*
LTC4S	0,47*
CRP	0,30*
COL4A5	0,28*
CPA2	0,27*

CCL3	2,20*
PDE4C	2,09*
NCAM1	1,85*
EREG	1,71*
PLA2G1B	1,69*
MMP9	1,81*
BDKRB1	6,79*
KLK3	4,61*
PLA2G2D	3,43*
PTGER3	3,01*
LTB4R2_A	2,11*
SELE	1,59*
ITGAM	1,57*
DEFA3	1,51*
PLA2G5	0,36*
PRKAA2	0,59*
DNM1P46	0,44*
HTR3B	0,24*
CD22	0,65*
KNG1	0,64*
CCL5	0,51*
CLEC5A	0,37*
PGK1	0,21*
HIF3A	0,19*

FOXP3	2,70*
IL2	1,93*
BDNF	1,92*
IL4	1,57*
CA4	1,57*
IL1RAPL2	1,53*
FGF21	1,53*
HSPA1B	0,64*
FGF6	0,60*
ABCA1	0,58*
SOCS3	0,56*
IL10	0,55*
HRH2	0,48*
ARNT2	0,38*
TNIP3	0,35*
IL9	0,16*
ORM1	0,66*
AGTR1	0,64*
FRZB	0,67*
HSPA1A	0,63*
ADIPOQ	0,40*

MC2R	2,97*
IL1R2	2,37*
FRZB	1,96*
C1QTNF9	0,35*
MXRA5	0,59*
CSF1	0,51*
BDNF	0,46*
IGF2	0,30*
CA4	0,29*
FCGR3B	0,11*
RNASE2	0,60*
AGTR1	0,37*
FGF21	0,27*
FGF6	0,18*

HSPA12A	3,09*
PTGIS	2,15*
LYZ	1,75*
C1QTNF9	0,47*
FGF21	0,17*
FOXP3	0,61*
IL1RAPL2	0,61*
MC2R	0,25*
MXRA5	0,12*

PDGFC	1,88*
PTGDS	1,59*
TFRC	0,67*
PARD6G	0,65*
GJB6	0,63*
PRM1	0,66*
CDA	0,63*
PTGFR	0,23*

CACNA2D1	3,41*
PRM1	2,03*
PTGDS	0,57*
MARC1	0,57*
PPARGC1A	0,57*

RTEL1	0,50*
MYH6	0,16*

THY1	0,04*
EPHB2	0,40*
IL3	0,56*
IL17A	0,38*
LTC4S	0,67*
BDKRB2	0,48*
PLA2G1B	0,30*
KLK15	0,10*
