# Supplementary material for: Inflammation-Related Genes Are Differentially Expressed in Lipopolysaccharide-Stimulated Peripheral Blood Mononuclear Cells after 3 Months of Resistance Training in Older Women
Source: Cells. 2024 Aug 25;13(17):1416. doi: 10.3390/cells13171416 (PMC11394400; doi:10.3390/cells13171416)
Supplement: Supplementary file 1 [file cells-13-01416-s001.zip › SALIMANS_LPS-RNAseq_Cells_Supplementary Table S3.pdf]

Supplementary Table S3 Overview of the training-induced effects on the expression of genes that significantly changed in at least one of the intervention groups. A. Orange: pro-inflammatory genes, B. Green: anti-inflammatory genes, C. Grey: genes of which the exact immune-modulatory role regarding exercise has not yet been described. Dark red: training-induced increase in upregulation following LPS stimulation, Pink: training-induced increase in downregulation following LPS stimulation, Light blue: training-induced decrease in upregulation following LPS stimulation, Dark blue: training-induced decrease in downregulation following LPS stimulation, Purple: Training-induced change in gene expression from upregulation to downregulation following LPS stimulation, Orange: Training-induced change in gene expression from downregulation to upregulation following LPS stimulation, White with black text: Genes expression was not altered after the three months intervention in LPS-challenged PBMCs (FC=1), White text: showing a significant foldchange (FC≤0.67 or FC≥1.5). White text: showing a tendency towards clinically relevant foldchange (1<FC<1.5 or 0.67<FC<1).*

		IST	SET	CON
**Genes**	**Name**	**T2/T0**	**T2/T0**	**T2/T0**
**A.**				
IL12B	*Interleukin 12 beta*	2,78*	1,55*	4,63*
EDN1	*Endothelin 1*	1,72*	1,53*	0,89
CSF2	*Colony Stimulating Factor 2*	4,95*	1,08	2,17*
DNM1P46	*Dynamin 1 pseudogene 46*	1,90*	0,42*	0,44*
AREG	*Amphiregulin*	3,41*	0,89	1,20
PLA2G2A	*Phospholipase A2 group V*	2,29*	6,71*	1,00
IFNG	*Interferon gamma*	1,36	2,02*	1,46
FCAR	*Fc fragment of IgA receptor*	1,07	2,96*	0,93
CCL3	*C-C motif chemokine ligand 3*	1,11	1,25	2,20*
CCL5	*C-C motif chemokine ligand 5*	1,08	0,94	0,51*
COL3A1	*Collagen type III alpha 1 chain*	3,37*	1,10	0,93
CXCL10	*C-X-C motif chemokine ligand 10*	8,94*	2,87*	2,66*
CXCL11	*C-X-C motif chemokine ligand 11*	23,82*	3,08*	7,26*
COL4A1	*Collagen type IV alpha 1 chain*	1,81*	2,60*	1,17
HLA-DRA	*Major histocompatibility complex, class II, DR alpha*	1,56*	1,08	1,29
PGF	*Placental growth factor*	1,89*	1,33	1,23
STAT1	*Signal transducer and activator of transcription 1*	1,70*	1,21	1,11
TNFSF13B	*TNF superfamily member 13b*	1,90*	1,12	1,24
CD68	*Cluster of Differentiation 68*	1,54*	1,11	0,84
TLR4	*Toll like receptor 4*	1,58*	1,01	0,95
CD36	*Cluster of differentiation 36*	2,25*	0,57*	0,77
ASPN	*Asporin*	2,11*	0,76	1,15
JAK2	*Janus kinase 2*	1,61*	0,90	1,11
TLR7	*Toll like receptor 7*	2,79*	0,69	1,21
IL18	*Interleukin 18*	1,59*	0,73	0,96
CPA2	*Carboxypeptidase A2*	2,47*	0,27*	1,70*
NCR1	*Natural cytotoxicity triggering receptor 1*	3,38*	0,52*	0,33*
VEGFC	*Vascular endothelial growth factor C*	1,17	2,19*	1,16
PLA2G2D	*Phospholipase A2 Group IID*	1,07	0,37*	3,43*
PTGER3	*Prostaglandin E receptor 3*	1,48	0,58*	3,01*
SELE	*Selectin 1*	1,16	0,65*	1,59*
CXCR1	*C-X-C Motif Chemokine Receptor 1*	1,14	0,11*	1,11
HLA-DRB5	*Major histocompatibility complex, class II, DR beta 5*	1,16	0,56*	1,08
TERC	*Telomerase RNA component*	1,32	0,49*	0,53*
CX3CL1	*C-X3-C motif chemokine ligand 1*	1,08	0,56*	1,05
HTR3B	*5-Hydroxytryptamine Receptor 3B*	1,15	0,60*	0,24*
CSF3	*Colony Stimulating Factor 3*	0,48*	7,19*	18,86*
IL6	*Interleukin 6*	0,67*	1,80*	5,05*
PTGS2	*Prostaglandin-endoperoxide synthase 2*	0,34*	1,60*	3,78*
HIF1A	*Hypoxia inducible factor 1 subunit alpha*	0,65*	1,00	1,02
CCL19	*C-C motif chemokine ligand 19*	0,52*	1,16	3,89*
CD14	*Cluster of Differentiation 14*	0,65*	1,29	1,16
S100A8	*S100 calcium binding protein A8*	0,56*	1,34	1,49
S100A9	*S100 calcium binding protein A9*	0,60*	1,29	1,42
IL2RA	*Interleukin 2 receptor subunit alpha*	0,55*	1,19	1,00
THY1	*Thy-1 cell surface antigen*	0,04*	6,34*	4,10*
IL1B	*Interleukin 1 beta*	0,59*	0,93	4,42*
TNFRSF1B	*TNF receptor superfamily member 1B*	0,63*	0,98	1,03
TREM1	*Colony Stimulating Factor 3 Receptor*	0,58*	0,92	1,49
VEGFA	*Vascular endothelial growth factor A*	0,45*	0,97	1,12
SLC2A4	*Solute carrier family 2 member 4*	0,63*	0,80	0,69
IL1A	*Interleukin 1 alpha*	0,88	1,20	5,64*
EREG	*Epiregulin*	0,71	0,98	1,71*

PDE4C	*Phosphodiesterase 4C*	0,71	0,93	2,09*
NCAM1	*Neural cell adhesion molecule 1*	0,85	0,62*	1,85*
LEP	*Leptin*	0,45*	0,08*	0,15*
EPHB2	*EPH receptor B2*	0,40*	0,95	0,76
IL3	*Interleukin 3*	0,56*	2,14*	3,57*
IL17A	*Interleukin 17A*	0,38*	1,89*	0,91
BDKRB2	*Bradykinin receptor B2*	0,48*	1,03	0,74
LTC4S	*Leukotriene C4 Synthase*	0,67*	0,47*	1,36
BMX	*BMX non-receptor tyrosine kinase*	0,72	0,61*	0,96
EDNRB	*Endothelin receptor type B*	0,67*	0,23*	1,07
DEFA3	*Defensin alpha 3*	0,93	1,34	1,51*
ITGAM	*Integrin subunit alpha M*	0,94	1,08	1,57*
KNG1	*Kininogen 1*	0,77	0,72	0,64*
NOS2	*Nitric oxide synthase 2*	0,94	2,34*	0,97
LTB4R2	*Leukotriene B4 receptor*	0,56*	0,32*	1,00
PLA2G1B	*Phospholipase A2 group IB*	0,30*	0,75	1,69*
KLK15	*Kallikrein related peptidase 15*	0,10*	3,20*	9,87*
MMP9	*Matrix metallopeptidase 9*	0,74	1,20	1,81*
COL4A5	*Collagen type IV alpha 5 chain*	0,68	0,28*	1,07
LTB4R2_A	*Leukotriene B4 receptor 2a*	3,90*	5,72*	2,11*
KLK2	*Kallikrein Related Peptidase 2*	1,52*	1,61*	0,80
CD38	*Cluster of Differentiation 38*	1,93*	1,47	1,35
CYR61	*Cysteine rich angiogenic inducer 61*	2,36*	1,41	1,13
PRKAA2	*Protein kinase AMP-activated catalytic subunit alpha 2*	21,43*	1,40	0,59*
CLEC5A	*C-type lectin domain containing 5A*	2,91*	1,55*	0,37*
BDKRB1	*Bradykinin receptor B1*	3,67*	1,27	6,79*
STAT2	*Signal transducer and activator of transcription 2*	1,55*	1,14	1,08
TNFSF10	*Tumor Necrosis Factor (Ligand) Superfamily, Member 10*	2,42*	1,16	1,44
PLA2G5	*Phospholipase A2 group V*	2,50*	1,40	0,36*
PGK1	*Phosphoglycerate kinase 1*	2,71*	0,16*	0,21*
IL1RL1	*Cluster of differentiation 160*	1,98*	0,81	1,01
IL12A	*Interleukin 12A*	1,84*	0,88	1,08
HIF3A	*Hypoxia inducible factor 3 alpha subunit*	2,62*	0,15*	0,19*
CRP	*C-reactive protein*	1,81*	0,30*	1,36
LRP2	*LDL receptor related protein 2*	7,83*	0,56*	0,72
CD22	*Cluster of Differentiation 22*	1,17	1,07	0,65*
KLK3	*Kallikrein Related Peptidase 3*	1,10	1,00	4,61*
**B.**				
THBD	*Thrombomodulin precursor*	1,73*	0,84	1,21
MXRA5	*Matrix remodeling associated 5*	4,42*	0,59*	0,12*
HSPA1L	*Heat shock 70 kDa protein 1L*	2,37*	0,40*	0,61*
IL13	*Interleukin 13*	1,02	1,65*	1,23
CA4	*Carbonic anhydrase 4*	1,57*	0,29*	0,71
FN1	*Fibronectin 1*	2,13*	0,75	1,63*
FGF21	*Fibroblast growth factor 21*	1,53*	0,27*	0,17*
IL1RAPL2	*Interleukin 1 receptor accessory protein like 2*	1,53*	1,00	0,61*
C1QTNF9	*C1q and tumor necrosis factor related protein 9*	1,43	0,35*	0,47*
FCGR3B	*Fc Fragment Of IgG Receptor IIIb*	1,18	0,11*	0,97
LYZ	*Lysozyme*	1,18	0,80	1,75*
TNIP3	*TNFAIP3 interacting protein 3*	0,35*	1,61*	1,99*
ARNT2	*Aryl hydrocarbon receptor nuclear translocator 2*	0,38*	2,89*	0,75
ABCA1	*ATP binding cassette subfamily A member 1*	0,58*	1,12	1,19
HRH2	*Histamine receptor H2*	0,48*	1,27	1,29
SOCS3	*Suppressor of cytokine signaling 3*	0,56*	1,47	1,42
IL9	*Interleukin 9*	0,16*	1,44	0,99
IL10	*Interleukin 10*	0,55*	0,89	1,13
HSPA1B	*Heat shock 70kDa protein 1B*	0,64*	0,97	1,05
FGF6	*Fibroblast growth factor 6*	0,60*	0,18*	1,85*
IL1R2	*Interleukin 1 receptor type 2*	0,55*	2,37*	0,51*
RFC2	*Replication factor C subunit 2*	0,32*	0,56*	1,00
AGTR1	*Angiotensin II receptor type 1*	0,64*	0,37*	3,63*
ORM1	*Orosomucoid 1*	0,66*	15,37*	0,79
FRZB	*Frizzled-related protein*	0,67*	1,96*	1,92*
HSPA1A	*Heat shock 70kDa protein 1A*	0,63*	1,27	1,10
PTGIS	*Prostaglandin I2 synthase*	0,12*	0,06*	2,15*
HRH3	*Histamine receptor H3*	0,50*	0,32*	0,86
ADIPOQ	*Adiponectin, C1Q and collagen domain containing*	0,40*	1,00	1,00

RNASE2	*Ribonuclease A family member 2*	0,73	0,60*	0,95
IL4	*Interleukin 4*	1,57*	0,95	1,22
CSF1	*Colony Stimulating Factor 1*	1,55*	0,51*	3,82*
BDNF	*Brain derived neurotrophic factor*	1,92*	0,46*	1,23
IL2	*Interleukin 2*	1,93*	0,92	0,72
FOXP3	*Forkhead box P3*	2,70*	0,86	0,61*
IGF2	*Insulin growth factor*	1,00	0,30*	1,36
MC2R	*Melanocortin 2 receptor*	1,00	2,97*	0,25*
HSPA12A	*Heat shock protein family A (Hsp70) member 12 A*	1,00	1,00	3,09*
**C.**				
CACNA2D1	*Calcium voltage-gated channel auxiliary subunit alpha2delta 1*	1,74*	3,41*	0,94
CDH1	*Cadherin 1*	1,32	1,09	1,53*
PTGDS	*Prostaglandin D2 synthase*	1,59*	0,57*	0,91
PDGFC	*Platelet derived growth factor C*	1,88*	0,88	0,77
GJB6	*Gap junction protein beta 6*	0,63*	1,58*	1,12
PARD6G	*Par-6 family cell polarity regulator gamma*	0,65*	1,14	1,03
TFRC	*Transferrin receptor*	0,67*	1,10	0,99
PDGFA	*Platelet derived growth factor subunit A*	0,69	1,52*	1,62*
CYP1A2	*Cytochrome P450 family 1 subfamily A member 2*	0,71	1,81*	0,68
CES1	*Carboxylesterase 1*	0,80	1,19	1,55*
MYH6	*Myosin heavy chain 6*	0,77	0,71	0,16*
MARC1	*Mitochondrial amidoxime reducing component*	0,91	0,57*	1,36
PPARGC1A	*Peroxisome proliferator-activated receptor gamma coactivator 1-alpha coactivator 1 alpha*	0,71	0,57*	0,83
ADRB1	*Adrenoceptor beta 1*	0,87	0,76	1,54*
CDA	*Cytidine deaminase*	0,63*	1,07	1,28
PTGFR	*Prostaglandin F receptor*	0,23*	7,22*	1,10
PRM1	*Protamine 1*	0,66*	2,03*	0,72
RTEL1	*Regulator of telomere elongation helicase 1*	0,96	1,03	0,50*
RPL3L	*Ribosomal protein L3 like*	1,70*	0,71	1,13
HPGD	*15-hydroxyprostaglandin dehydrogenase*	1,76*	0,76	0,97
CYP7A1	*Cytochrome P450 family 7 subfamily A member 1*	2,04*	3,82*	1,29
COL4A2	*Collagen type IV alpha 2 chain*	1,18	0,66*	0,57*
OLIG2	*Oligodendrocyte lineage transcription factor 2*	1,58*	2,17*	2,65*
